# Design and evaluation of selective butyrylcholinesterase inhibitors based on *Cinchona* alkaloid scaffold

**DOI:** 10.1371/journal.pone.0205193

**Published:** 2018-10-05

**Authors:** Anita Bosak, Alma Ramić, Tamara Šmidlehner, Tomica Hrenar, Ines Primožič, Zrinka Kovarik

**Affiliations:** 1 Institute for Medical Research and Occupational Health, Ksaverska cesta 2, Zagreb, Croatia; 2 Faculty of Science, Horvatovac 102A, University of Zagreb, Zagreb, Croatia; Weizmann Institute of Science, ISRAEL

## Abstract

This paper describes the synthesis and anticholinesterase potency of *Cinchona*-based alkaloids; ten quaternary derivatives of cinchonines and their corresponding *pseudo*-enantiomeric cinchonidines. The quaternization of quinuclidine moiety of each compound was carried out with groups diverse in their size: methyl, benzyl and differently *meta-* and *para-*substituted benzyl groups. All of the prepared compounds reversibly inhibited human butyrylcholinesterase and acetylcholinesterase with *K*_i_ constants within nanomolar to micromolar range. Five cinchonidine derivatives displayed 95–510 times higher inhibition selectivity to butyrylcholinesterase over acetylcholinesterase and four were potent butyrylcholinesterase inhibitors with *K*_i_ constants up to 100 nM, of which *N*-*para*-bromobenzyl cinchonidinium bromide can be considered a lead for further modifications and optimizations for possible use in the treatment of neurodegenerative diseases.

## Introduction

Vertebrates possess two cholinesterases, acetylcholinesterase (AChE; EC 3.1.1.7) and butyrylcholinesterase (BChE; EC 3.1.1.8) that are responsible in the organism for hydrolysing the neurotransmitter acetylcholine. By degradation of acetylcholine, AChE fulfills its physiological role allowing maintence of optimal neurotransmission. This role is shared by AChE with the related enzyme BChE that does not possess a known physiological substrate, but does have a role in the bioconversion of several xenobiotics, in the metabolism of lipoproteins and, in cases when the activity of AChE is low or inhibited, serves as AChE backup enzyme [1]. BChE and AChE share almost the same backbone structure with a more than 50% identical amino acid sequence and an active site located in a 20 Å deep gorge [2–4]. The active site of AChE and BChE is divided into two sub-sites; the peripheral anionic site (PAS) located at the entrance and the catalytic site located at the bottom of the gorge. Although both enzymes have the same composition of the catalytic triad and an oxyanion hole, some aromatic amino acids from the AChE active site are substituted in BChE with aliphatic ones, resulting in a different selectivity in interactions with substrates and inhibitors, as well as a different stereoselectivity [5–8]. Both AChE and BChE are stereoselective in the interaction with various esters such as phosphonates, acetate derivatives of quinuclidin-3-ols, and carbamates [5, 6, 9–11].

Both AChE and BChE are crucial in the treatment of neurodegenerative disorders such as myasthenia gravis, Alzheimer and Parkinson’s disease, since so far the most successful approach in treating these disorders has been the use of cholinesterase inhibitors that target primary AChE [12, 13]. Over the past decades, rivastigmine and the alkaloid galantamine, which inhibit both AChE and BChE, and the synthetic donepezil whose primary target is AChE, have been launched on the market [14]. Recent studies on the impact of brain BChE on the symptoms and progression of cognitive impairments promoted BChE as an important target in future Alzheimer disease pharmacotherapy [15]. So far, many compounds with various structural scaffolds have been determined to selectively inhibit BChE [5, 6, 9, 16–19].

**Fig 1:**
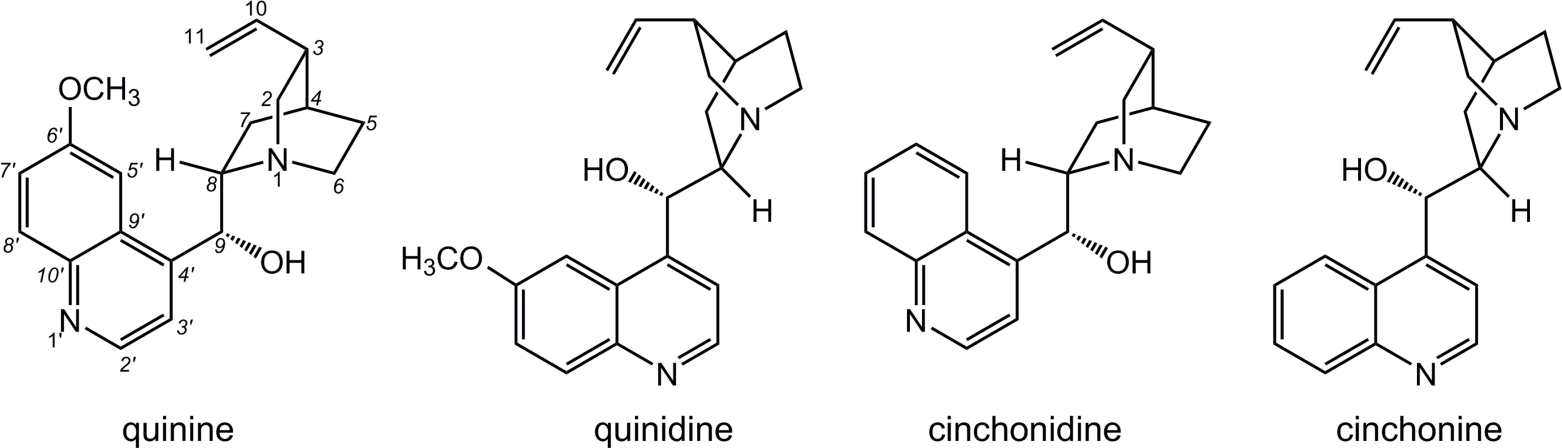
*Cinchona* alkaloids.

The bark of *Cinchona* trees is the source of a variety of alkaloids, among which the best known are quinine, quinidine, cinchonine and cinchonidine (Fig 1). These alkaloids are very useful in organic chemistry as organocatalysts for asymmetric synthesis and have been investigated for that purpose for more than 35 years. Their derivatives are known as the most preferred inducers of chirality, which successfully catalyse numerous classes of organic reactions with a high degree of stereoselectivity [20]. Furthermore, these alkaloids are bioactive and are used in treating malaria and fever, while some also possess analgesic, anti-inflammatory and antiarrhythmic properties [21]. Recently, some cinchonine and cinchonidines were proven to be up to 100 times more potent inhibitors for equine BChE than human AChE, while anthracene/benzyl modified cinchonidine has been identified as selective BChE inhibitors with a BChE/AChE selectivity ratio of 250 [22, 23]. In addition, a high affinity for binding to the active site of BChE was determined for some *Cinchona* oxime compounds studied as reactivators of OP-inhibited human BChE [24].

**Fig 2:**
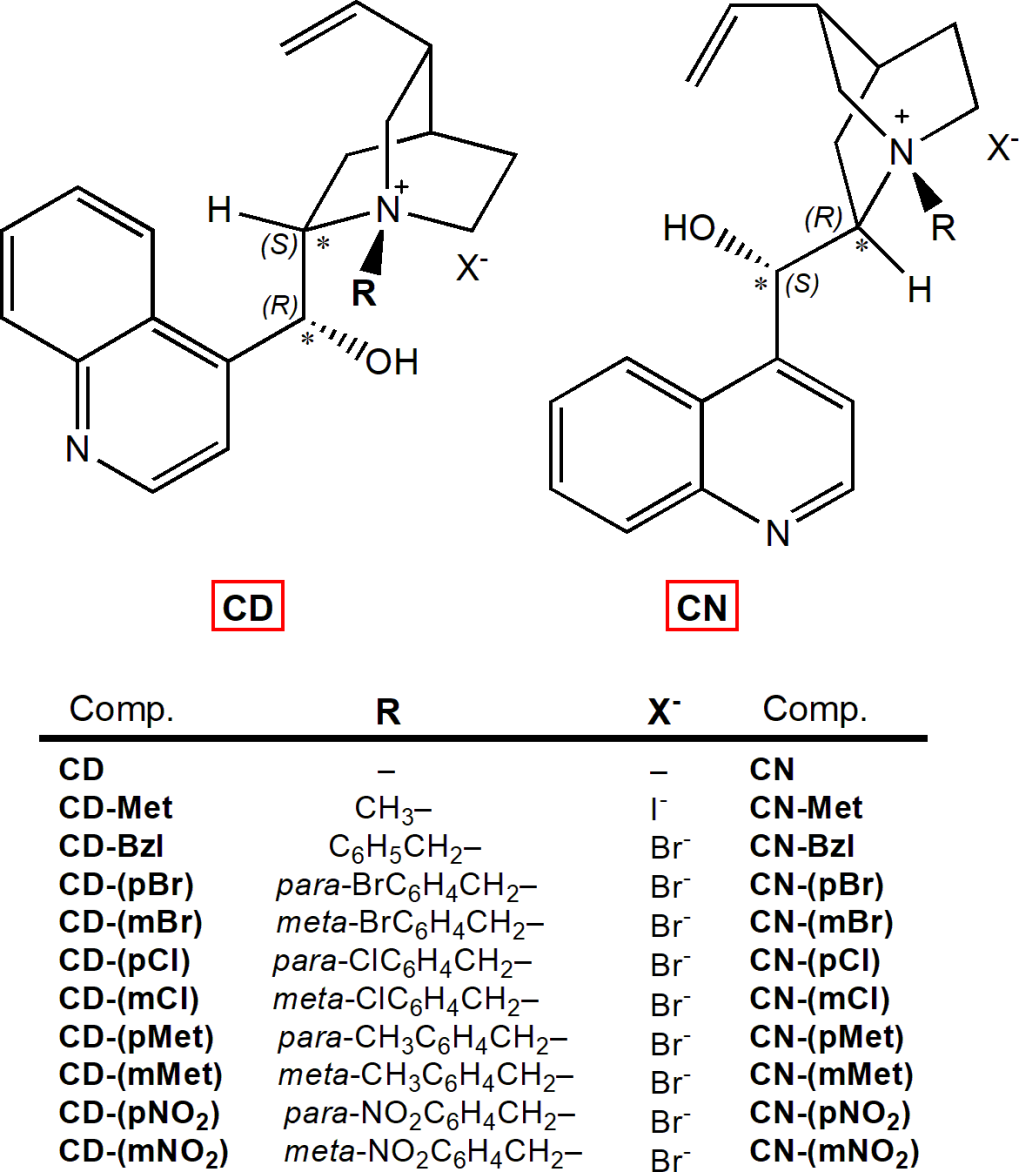
Structures of cinchonidine (CD) and cinchonine (CN) compounds. Absolute configurations, opposite in *pseudo*-enantiomers at positions 8 and 9, are marked with an asterisk and noted; (8S,9R) in cinchonidine and (8R,9S) in cinchonine.

In this study, we synthesised a series of 20 compounds; ten synthetic quaternary derivatives of cinchonidines and ten of their corresponding *pseudo*-enantiomers cinchonines (Fig 2). Six compounds were synthesised for the first time. Quaternization of quinuclidine moiety was carried out with groups diverse in size: methyl, benzyl and differently *meta-* and *para-*substituted benzyl groups. The aim of the work was to determine their inhibition potency toward human BChE and AChE, and evaluate their inhibition selectivity, which was expected also according to recent studies on quinine and quinidine derivatives [22–24]. Therefore, we determined the stereoselectivity of cholinesterases toward *pseudo*-enantiomeric pairs of cinchonines and cinchonidines. Furthermore, the *in vitro*-determined affinity of the studied compounds, their inhibition selectivity as well as stereoselectivity of cholinesterases, were interpreted and defined by molecular modelling.

## Material and methods

### Chemicals

All of the chemicals, reagents and solvents for the preparation of cinchonines and their corresponding *pseudo*-enantiomeric cinchonidines were purchased from commercial sources and used without further purification.

Acetylthiocholine (ATCh) and 5,5´-dithiobis(2-nitrobenzoic acid) (DTNB) were purchased from Sigma Chemical Co., USA. ATCh was dissolved in water and DTNB in 0.1 M sodium phosphate buffer (pH 7.4). Generally, cinchonines and cinchonidines were dissolved in water, and for some a small quantity of HCl was added (final concentration up to 0.5% v/v). The exceptions were compounds CN-Met, CD-Bzl, CN-Bzl and CD-(pCH3) dissolved in phosphate buffer 0.1 M, pH 7.4, and compounds CD-(pCl) and CN-(pCl) dissolved in DMSO. All further dilutions were made in water.

### Enzymes

Purified human BChE and recombinant human AChE were kindly provided by Dr. F. Nachon (Département de Toxicologie, Armed Forces Biomedical Research Institute, France). The concentration of enzymes (BChE: 5.6 μM; AChE: 0.20 μM) was determined as described previously [10]. The concentrated stocks of enzymes were diluted in a phosphate sodium buffer 0.1 M (pH 7.4) containing 0.1% BSA.

### Synthesis

The compounds were synthesized following standard procedures for the Menshutkin reaction [25–28] starting from commercially available cinchonidine or cinchonine (≥98.0%, Sigma-Aldrich, St. Louis, MO, USA). *Cinchona* alkaloid (1 mmol) and appropriate halide (1.05 mmol for *para*- or 1.2 mmol for *meta*-substituted benzyl bromide) in toluene were heated to reflux and end of reaction was detected with thin layer chromatography (CHCl_3_: MeOH = 9:1). After cooling to 22 °C, the precipitated product was isolated by filtration and recrystallized from methanol/diethyl ether or acetonitrile/diethyl ether. The reactions were monitored and the purity of products was checked by thin-layer chromatography plates coated with silica gel (Sigma-Aldrich, St. Louis, MO, USA). TLC plates were visualized by UV irradiation (254 nm) or by iodine fumes. Melting points were determined on a Melting Point B-540 apparatus (Büchi, Germany) and are uncorrected. Optical rotations were measured on an Optical Activity AA-10 automatic polarimeter (Optical Activity Limited, Ramsey, Cambridgeshire, UK) at 22 °C. High resolution mass spectra (HRMS) were obtained on a 4800 plus MALDI TOF/TOF instrument (Applied Biosystems Inc, Foster City, CA, USA). CHN analysis was performed on Perkin Elmer 2400 Series II CHNS analyser and all compounds were found to be of ≥99% purity. ^1^H and ^13^C NMR spectra were recorded on a Varian XL-GEM 600 spectrometer at 22 °C and Bruker Avance III HD 400 MHz/54 mm Ascend spectrometer (Bruker Optics Inc, Billerica, MA, USA). Chemical shifts are given in ppm downfield from TMS as internal standard and coupling constants (*J*) in Hz. Splitting patterns were designated as s (singlet), d (doublet), dd (doublet of doublets), ddd (doublet of doublet of doublets), t (triplet), q (quartet) or m (multiplet). Benzene hydrogen and carbon atoms are marked with a double apostrophe.

All of the compounds were characterized by standard analytical spectroscopic methods (NMR, IR, MS) and elemental analysis. The data for the novel compounds are presented below, while the data for all of the other compounds are given in the S1 File.

#### *N*-(4-methylbenzyl) cinchoninium bromide, CN-(pMet)

White solid. Yield: 80% (recrystallized from methanol/diethyl ether). m.p. 231.2–232 °C; ${⦋\alpha ⦌}_{D}^{24}=+186{}^{\circ}\;(c\;0.1,MeOH)$; IR (cm^-1^): 3433 (O-H), 3120 (C-H_Ar_), 1511 (C = N), 1068 (C-N); HRMS (ES+) calc. for C_27_H_31_N_2_O: 399.2436 found 399.2439; Anal. Calc. for [C_27_H_31_BrN_2_O]: C, 67.64; H, 6.52; N, 5.84 found C, 67.65; H, 6.54; N, 5.89; ^1^H NMR (600 MHz, DMSO-*d*_6_) δ ppm 1.01–1.08 (1 H, m, H7b) 1.71–1.80 (2 H, m, H5) 1.87 (1 H, m, H4) 2.29 (1 H, t, *J* = 11.4 Hz, H7a) 2.41 (3 H, s, CH_3_) 2.65 (1 H, q, *J* = 8.56 Hz, H3) 2.91–2.99 (1 H, m, H2b) 3.47 (1 H, t, *J* = 11.4 Hz, H6b) 3.88–3.97 (2 H, m, H8, H2a) 4.17–4.24 (1 H, m, H6a) 4.92 (1 H, d, *J* = 12.5 Hz, H11b) 5.06–5.10 (1 H, m, H11a) 5.19–5.27 (2 H, m, CH_2_) 6.01 (1 H, ddd, *J* = 17.1, 10.5, 7.0 Hz, H10) 6.52 (1 H, s, H9) 6.81 (1 H, d, *J* = 3.7 Hz, OH) 7.39 (2 H, m, *J* = 7.4 Hz, H3’, H6’) 7.62–7.67 (2 H, m, H3”, H5”) 7.72–7.77 (1 H, m, H7’) 7.82–7.88 (2 H, m, H2”, H6”) 8.12 (1 H, d, *J* = 8.1 Hz, H5’) 8.32–8.38 (1 H, m, H8’) 8.99 (1 H, d, *J* = 4.4 Hz, H2’); ^13^C NMR (151 MHz, DMSO-*d*_6_) δ ppm 20.6 (C7) 20.8 (CH_3_) 22.9 (C5) 26.3 (C4) 36.6 (C3) 53.6 (C6) 55.9 (C2) 62.1 (CH_2_) 64.7 (C9) 67.0 (C8) 117.0 (C11) 120.0 (C3’) 123.8 (C5’) 124.3 (C4”) 124.8 (C1”) 127.2 (C6’) 129.4 (C7’) 129.5 (C2”, C6”) 129.7 (C8’) 133.6 (C3”, C5”) 137.1 (C10) 139.8 (C4”) 145.0 (C10’) 147.6 (C4’) 150.1 (C2’).

#### *N*-(3-bromobenzyl) cinchonidinium bromide, CD-(mBr)

White solid. Yield: 87% (recrystallized from ethanol/diethyl ether). m.p. 181.3–182.0 °C; ${⦋\alpha ⦌}_{D}^{24}=-181{}^{\circ}\;(c\;0.1,MeOH)$; IR(cm^-1^): 3140 (O-H), 3453 (C-H_Ar_), 1509 (C = N), 1060 (C-N); HRMS (ES+) calc. for C_26_H_28_BrN_2_O: 463.1385 found 463.1389; Anal. Calc. for [C_26_H_28_Br_2_N_2_O]: C, 62.47; H, 5.65; N, 5.60 found C, 62.44; H, 5.66; N, 5.63; ^1^H NMR (600 MHz, DMSO-*d*_6_) δ ppm 1.26–1.32 (1 H, m, H7b) 1.82 (1 H, t, *J* = 10.3 Hz, H5b) 1.98–2.02 (1 H, m, H4) 2.03–2.09 (1 H, m, H5a) 2.10–2.15 (1 H, m, H7a) 2.69 (1 H, m, H3) 3.27 (1 H, td, *J* = 11.6, 4.8 Hz, H6b) 3.44 (1 H, qd, *J* = 7.1, 5.2 Hz, H2b) 3.76 (1 H, d, *J* = 12.5 Hz, H2a) 3.89 (1 H, t, *J* = 8.8 Hz, H8) 4.29 (1 H, t, *J* = 10.3 Hz, H6a) 4.96 (1 H, d, *J* = 10.3 Hz, H11a) 5.04 (1 H, d, *J* = 12.5 Hz, H11b) 5.14–5.20 (2 H, m, CH_2_) 5.68 (1 H, ddd, *J* = 17.2, 10.6, 6.6 Hz, H10) 6.52–6.55 (1 H, m, H9) 6.72 (1 H, d, *J* = 4.4 Hz, OH) 7.55 (1 H, t, *J* = 8.1 Hz, H3’) 7.74–7.78 (2 H, m, H6”, H5”) 7.78–7.82 (3 H, m, H2”, H4”, H6’) 7.85 (1 H, t, *J* = 7.7 Hz, H7’) 8.11 (1 H, d, *J* = 8.1 Hz, H5’) 8.29 (1 H, d, *J* = 8.8 Hz, H8’) 8.99 (1 H, d, *J* = 4.4 Hz, H2’); ^13^C NMR (151 MHz, DMSO-*d*_6_) δ ppm 20.9 (C7) 24.2 (C5) 25.8 (C4) 36.8 (C3) 50.7 (C6) 59.2 (C2) 61.8 (CH_2_) 64.0 (C9) 67.8 (C8) 116.3 (C11) 120.0 (C3’) 122.00 (C9’) 123.6 (C5’) 124.2 (C3”) 127.2 (C6’) 129.4 (C7’) 129.9 (C8’) 130.5 (C1”) 131.0 (C5”) 132.9 (C6”) 133.0 (C4”) 136.1 (C2”) 138.1 (C10) 145.1 (C10’) 147.6 (C4’) 150.2 (C2’).

#### *N*-(3-methylbenzyl) cinchonidinium bromide, CD-(mMet)

White solid. Yield: 48% (recrystallized from acetonitrile/diethyl ether). m.p. 182–183 °C; ${⦋\alpha ⦌}_{D}^{24}=-203{}^{\circ}\;(c\;0.1,MeOH)$; IR (cm^-1^): 3423 (O-H), 3137 (C-H_Ar_), 1502 (C = N), 1054 (C-N), HRMS (ES+) calc. for C_27_H_31_N_2_O: 399.2436 found 399.2442; Anal. Calc. for [C_27_H_31_BrN_2_O]: C, 67.64; H, 6.52; N, 5.84 found C, 67.60; H, 6.54; N, 5.82; ^1^H NMR (300 MHz, DMSO-*d*_6_) δ ppm 1.24–1.36 (1 H, m, H7b) 1.83 (1 H, t, *J* = 9.0 Hz, H5b) 1.97–2.04 (1 H, m, H4) 2.05–2.19 (2 H, m, H5a, H7a) 2.40 (3 H, s, CH_3_) 2.70 (1 H, m, H3) 3.20–3.30 (1 H, m, H6b) 3.71–3.80 (1 H, m, H2a) 3.93 (1 H, t, *J* = 8.7 Hz, H8) 4.23–4.35 (1 H, m, H6a) 4.92–5.03 (2 H, m, H11) 5.13–5.21 (2 H, m, CH_2_) 5.69 (1 H, ddd, *J* = 17.2, 10.6, 6.4 Hz, H10) 6.55 (1 H, d, *J* = 3.8 Hz, H9) 6.73 (1 H, d, *J* = 4.5 Hz, OH) 7.36–7.41 (1 H, m, H3’) 7.46 (1 H, t, *J* = 7.7 Hz, H7’) 7.52–7.57 (2 H, m, H4”, H2”) 7.71–7.78 (1 H, m, H6’) 7.78–7.89 (2 H, m, H6”, H5”) 8.11 (1 H, dd, *J* = 8.5, 0.9 Hz, H5’) 8.31 (1 H, d, *J* = 7.9 Hz, H8’) 8.98 (1 H, d, *J* = 4.5 Hz, H2’); ^13^C NMR (75 MHz, DMSO-*d*_6_) δ ppm 21.4 (CH_3_) 21.4 (C7) 24.7 (C5) 26.4 (C4) 37.4 (C3) 51.1 (C6) 59.8 (C2) 63.2 (CH2) 64.5 (C9) 68.0 (C8) 116.7 (C11) 120.5 (C3’) 124.2 (C5’) 124.8 (C9’) 127.7 (C6’) 128.3 (C1”) 129.3 (C7’) 129.9 (C8’) 130.3 (C3”, C5”) 131.2 (C6”) 131.3 (C4”) 134.7 (C2”) 138.7 (C10) 145.7 (C10’) 148.1 (C4’) 150.6 (C2’).

#### *N*-(3-methylbenzyl) cinchoninium bromide, CN-(mMet)

White solid. Yield: 47% (recrystallized from acetonitrile/diethyl ether); m.p. 225.1–241.5 °C; ${⦋\alpha ⦌}_{D}^{24}=+161{}^{\circ}\;(c\;0.1,MeOH)$; IR (cm^-1^): 3425 (O-H), 3139 (C-H_Ar_), 1503 (C = N), 1054 (C-N), HRMS (ES+) calc. for C_27_H_31_N_2_O: 399.2436 found 399.2431; Anal. Calc. for [C_27_H_31_BrN_2_O]: C, 67.64; H, 6.52; N, 5.84 found C, 67.68; H, 6.51; N, 5.86; ^1^H NMR (300 MHz, DMSO-*d*_6_) δ ppm 0.98–1.10 (1 H, m, H7b) 1.69–1.83 (2 H, m, H5) 1.87 (1 H, m, H4) 2.29 (1 H, t, *J* = 11.5 Hz, H7a) 2.42 (3 H, s, CH_3_) 2.59–2.73 (1 H, m, H3) 2.91–3.03 (1 H, m, H2b) 3.51 (1 H, t, *J* = 11.1 Hz, H6b) 3.83–4.03 (2 H, m, H2a, H8) 4.22 (1 H, t, *J* = 9.8 Hz, H6a) 4.90 (1 H, d, *J* = 12.4 Hz, H11b) 5.07 (1 H, d, *J* = 12.1 Hz, H11a) 5.19–5.31 (2 H, m, CH_2_) 6.01 (1 H, ddd, *J* = 17.7, 10.2, 6.8 Hz, H10) 6.52 (1 H, s, H9) 6.80 (1 H, d, *J* = 3.8 Hz, OH) 7.36–7.42 (1 H, m, H3’) 7.46 (1 H, t, *J* = 7.4 Hz, H7’) 7.52–7.59 (2 H, m, H5”, H6”) 7.71–7.78 (1 H, m, H6’) 7.80–7.89 (2 H, m, H2”, H4”) 8.11 (1 H, d, *J* = 7.9 Hz, H5’) 8.33 (1 H, d, *J* = 8.3 Hz, H8’) 8.99 (1 H, d, *J* = 4.1 Hz, H2’); ^13^C NMR (75 MHz, DMSO-*d*_6_) δ ppm 21.1 (CH_3_) 21.4 (C7) 23.4 (C5) 26.8 (C4) 37.1 (C3) 54.3 (C6) 56.5 (C2) 62.8 (CH_2_) 65.2 (C9) 67.6 (C8) 117.5 (C11) 120.5 (C3’) 124.3 (C5’) 124.8 (C9’) 127.7 (C6’) 128.2 (C1”) 129.3 (C7’) 129.9 (C8’) 130.3 (C6”) 131.2 (C5”) 131.3 (C4”) 134.7 (C2”) 137.6 (C10) 138.7 (C3”) 145.5 (C10’) 148.1 (C4’) 150.6 (C2’).

#### *N*-(3-chlorobenzyl) cinchonidinium chloride, CD-(mCl)

White solid. Yield: 27% (recrystallized from ethanol/diethyl ether). m.p. 217.4–217.7 °C; ${⦋\alpha ⦌}_{D}^{24}=-258{}^{\circ}\;(c\;0.1,MeOH)$; IR(cm^-1^): 3415 (O-H), 3085 (C-H_Ar_), 1511 (C = N), 1100 (C-N); HRMS (ES+) calc. for C_26_H_28_ClN_2_O: 419.1890 found 419.1884; Anal. Calc. for [C_26_H_28_Cl_2_N_2_O]: C, 68.57; H, 6.20; N, 6.15 found C, 68.55; H, 6.21; N, 6.18; ^1^H NMR (600 MHz, DMSO-*d*_6_) δ ppm 1.25–1.32 (1 H, m, H7b) 1.80 (1 H, t, *J* = 9.2 Hz, H5b) 1.97–2.02 (1 H, m, H4) 2.03–2.09 (1 H, m, H5a) 2.12 (1 H, dd, *J* = 13.2, 8.1 Hz, H7a) 2.67 (1 H, m, H3) 3.23 (1 H, td, *J* = 11.7, 4.4 Hz, H6b) 3.32–3.37 (1 H, m, H2b) 3.67–3.75 (1 H, m, H2a) 3.90 (1 H, t, *J* = 8.8 Hz, H8) 4.24 (1 H, t, *J* = 10.6 Hz, H6a) 4.95 (1 H, d, *J* = 11.0 Hz, H11a) 4.99 (1 H, d, *J* = 12.5 Hz, H11b) 5.11–5.19 (2 H, m, CH_2_) 5.67 (1 H, ddd, *J* = 17.2, 10.6, 6.6 Hz, H10) 6.53 (1 H, s, H9) 6.72 (1 H, d, *J* = 3.7 Hz, OH) 7.69 (2 H, d, *J* = 8.1 Hz, H6’, H3’) 7.73–7.77 (1 H, m, H4”) 7.78–7.82 (3 H, m, H2”, H6”, H5” 7.85 (1 H, t, *J* = 8.1 Hz, H7’) 8.11 (1 H, d, *J* = 7.3 Hz, H5’) 8.28 (1 H, d, *J* = 8.1 Hz, H8’) 8.98 (1 H, d, *J* = 4.4 Hz, H2’); ^13^C NMR (101 MHz, DMSO-*d*_6_) δ ppm 21.6 (C7) 24.7 (C5) 26.3 (C4) 37.3 (C3) 50.9 (C6) 59.8 (C2) 61.8 (CH2) 64.1 (C9) 68.5 (C8) 116.8 (C11) 120.7 (C3’) 124.2 (C5’) 124.8 (C9’) 127.7 (C6’) 129.8 (C7’) 130.3 (C8’) 130.5 (C6”) 131.0 (C5”) 131.1 (C1”) 133.1 (C4”) 133.9 (C2”) 138.6 (C10) 145.8 (C10’) 148.1 (C4’) 150.6 (C2’).

#### *N*-(3-nitrobenzyl) cinchonidinium bromide, CD-(mNO_2_)

White solid. Yield: 60% (recrystallized from acetonitrile/diethyl ether). m.p. 232.2–232.4 °C; ${⦋\alpha ⦌}_{D}^{24}=-259{}^{\circ}\;(c\;0.1,MeOH)$, IR(cm^-1^): 3415 (O-H), 3176 (C-H_Ar_), 1527 (N-O), 1509 (C = N), 1346 (N-O), 1062 (C-N); HRMS (ES+) calc. for C_26_H_28_N_3_O_3_: 430.2131 found 430.2138, Anal. Calc. for [C_26_H_28_BrN_3_O_3_]: C, 61.18; H, 5.53; N, 8.23 found C, 61.20; H, 5.51; N, 8.25; ^1^H NMR (600 MHz, DMSO-*d*_6_) δ ppm 1.28–1.36 (1 H, m, H7) 1.80 (1 H, t, *J* = 9.2 Hz, H5b) 2.02 (1 H, m, H4) 2.07–2.19 (1 H, m, H5a 2.65 (1 H, m, H3) 3.29 (1 H, td, *J* = 11.7, 4.40 Hz, H6b) 3.38 (1 H, d, *J* = 4.4 Hz, H2b) 3.84 (1 H, d, *J* = 12.5 Hz, H2a) 3.96 (1 H, t, *J* = 8.8 Hz, H8) 4.36 (1 H, t, *J* = 10.6 Hz, H6a) 4.96 (1 H, d, *J* = 10.3 Hz, H11a) 5.18 (1 H, d, *J* = 17.6 Hz, CH_2_) 5.25 (1 H, d, *J* = 12.5 Hz, H11b) 5.37 (1 H, d, *J* = 12.5 Hz, CH_2_) 5.68 (1 H, ddd, *J* = 17.2, 10.6, 6.6 Hz, H10) 6.57 (1 H, s, H9) 6.74 (1 H, d, *J* = 4.4 Hz, OH) 7.77 (1 H, t, *J* = 7.7 Hz, H3’) 7.81–7.94 (3 H, m, H6”, H5”, H6’) 8.12 (1 H, d, *J* = 8.1 Hz, H7’) 8.23 (1 H, d, *J* = 7.3 Hz, H5’) 8.32 (1 H, d, *J* = 8.1 Hz, H8’) 8.40–8.46 (1 H, m, H4”) 8.68 (1 H, m, H2”) 8.99 (1 H, d, *J* = 4.4 Hz, H2’); ^13^C NMR (151 MHz, DMSO-*d*_6_) δ ppm 20.9 (C7) 24.2 (C5) 25.8 (C4) 36.9 (C3) 50.7 (C6) 59.2 (C2) 61.4 (CH2) 64.1 (C9) 68.0 (C8) 116.4 (C11) 120.1 (C3’) 123.6 (C5’) 124.3 (C9’) 124.9 (C4”) 127.2 (C6’) 128.4 (C2”) 129.4 (C7’) 129.9 (C8’) 129.9 (C1”) 130.5 (C3”) 138.0 (C10) 140.2 (C4”) 145.1 (C10’) 147.6 (C3”) 148.0 (C4’) 150.1 (C2’).

### Enzyme activity measurements

Enzyme activities were measured spectrophotometrically by Ellman method at 412 nm using 0.30 mM DTNB as thiol reagent and ATCh (0.050–0.50 mM) as substrate in 0.1 M phosphate buffer, pH 7.4 [29, 30]. For the inhibition, the reaction mixture also contained cinchonidines or cinchonines (final concentrations 0.020–200 μM, depending on compound). In the case of compounds CD-(pCl) and CN-(pCl), the final content of DMSO was the same in enzyme activity measurement and in inhibition measurements, up to 0.32%. No side interactions of tested compounds with ATCh or DTNB were detected. Measurements were done at 25 °C on a Tecan Infinite M200Pro plate reader (Austria).

### Enzyme-inhibitor dissociation constants

The reversible inhibition of BChE and AChE by cinchonines and cinchonidines was measured by determining the decrease of enzyme activity towards ATCh (0.050–0.50 mM) in their presence. The activities of the enzymes were measured at different substrate concentrations ([S]) in the absence (*v*_0_) and presence (*v*_i_) of given cinchonines and cinchonidines concentration ([I]) selected to inhibit the enzymes for 20–80%. At least three concentrations of inhibitors for each substrate concentration were used in at least two experiments. The apparent dissociation inhibition constant (*K*_i,app_) was calculated using the Hunter-Downs equation and the linear regression analysis [31]:

$$K_{i,app}=\frac{v_{i}}{v_{0}-v_{i}}\cdot [I]=K_{\left(I\right)}+\frac{K_{\left(I\right)}}{K_{\left(S\right)}}\cdot [S]$$

where y-intercept determines the enzyme-inhibitor dissociation constants (*K*_(I)_), while x-intercept determines the enzyme-substrate dissociation constant, *K*_(S)_. The equation was used with the assumption that the substrate, due to relatively low substrate concentrations used in experiments, binds only to the catalytic site, while inhibitor can bind to both sites catalytic and peripheral site [31]. In other words, if the inhibitor competes with substrate for binding to the catalytic site of the enzyme, *K*_i,app_ proportionally depend on the substrate concentration. In case of non-competitive binding as the inhibitor binds to the peripheral site, *K*_i,app_ does not depend on substrate concentration. However, along with these simple cases of competitive or non-competitive inhibition, the Hunter-Downs plot indicates the mixed inhibition in case of non-linear plot. The curve means that the inhibitor binds not only to the catalytic or peripheral site but also to intermediates of Michaelis type of the enzyme-substrate complex.

The determination of kinetic constants was carried out using the GraphPadPrism 6.0 program (GraphPad Software, San Diego, USA).

### Molecular modelling

The docking of the studied compounds was performed by the Accelrys DiscoveryStudio 17.2 (Dassault Systèmes Biovia Corp., San Diego, USA) CDOCKER docking protocol and CHARMm force field using PDB ID 4PQE [32] and PDB ID 2PM8 [33] as a model for human AChE and BChE, respectively. As a result of molecular docking, a set of 20 possible poses per one compound and enzyme pair was analysed and the pose with the highest CDOCKER interaction energy was selected for further analysis. A detailed description of the docking protocol was given earlier [34].

Quantum mechanical docking calculation was performed by the systematic scanning of the active site using a combinatorial search algorithm implemented in our program code *qcc* [35]. Three translational degrees of freedom were scanned with a step of 0.5 Å whereas the three rotational degrees of freedom were scanned with increments of 30°. Single point calculation for the model of active site and investigated compound was performed using the PM6 method from Gaussian 09 program package [36]. All energy values from single point calculations were arranged in the 6-way array and search for all local minima was performed using a combinatorial algorithm built in our program for multivariate analysis *moonee* [37]. The selected local minima were subjected to a geometry optimization procedure using the combined quantum mechanical/quantum mechanical scheme, QM/QM 2-layer ONIOM approach with semiempirical PM6 method for the outer layer, and density functional theory B3LYP/6-31G(d) method, for the inner layer of the system [38, 39]. The results were inspected visually and on the basis of the energy values.

## Results and discussion

### Synthesis of compounds

Twenty quaternary derivatives of cinchonidines (CD compounds) and their corresponding *pseudo*-enantiomers cinchonines (CN compounds) were synthesized following standard procedures for the Menshutkin reaction [25–28] starting from commercially available cinchonidine (CD) or cinchonine (CN) (Fig 2; detailed in S1 File). Compounds CD-(mBr), CD-(mMet), CD-(mCl), CD-(mNO_2_) CN-(pMet), and CN-(mMet) were novel compounds. Quaternization of quinuclidine moiety was carried out with groups different in their size: methyl, benzyl and differently *meta-* and *para-*substituted benzyl groups. Compounds CD-Met and CN-Met are the only one with the aliphatic methyl group attached to the nitrogen atom of the quinuclidinium part of the molecule, while other compounds have an alkylaromatic group whose properties are modified with substituents in *para* or *meta* position on the benzene ring with different electron donating and electron withdrawing groups. All reactions proceeded with moderate to good yields. Compounds were characterized by standard analytical methods (IR, NMR, MS, CHN analysis).

### Inhibition of cholinesterases

**Fig 3:**
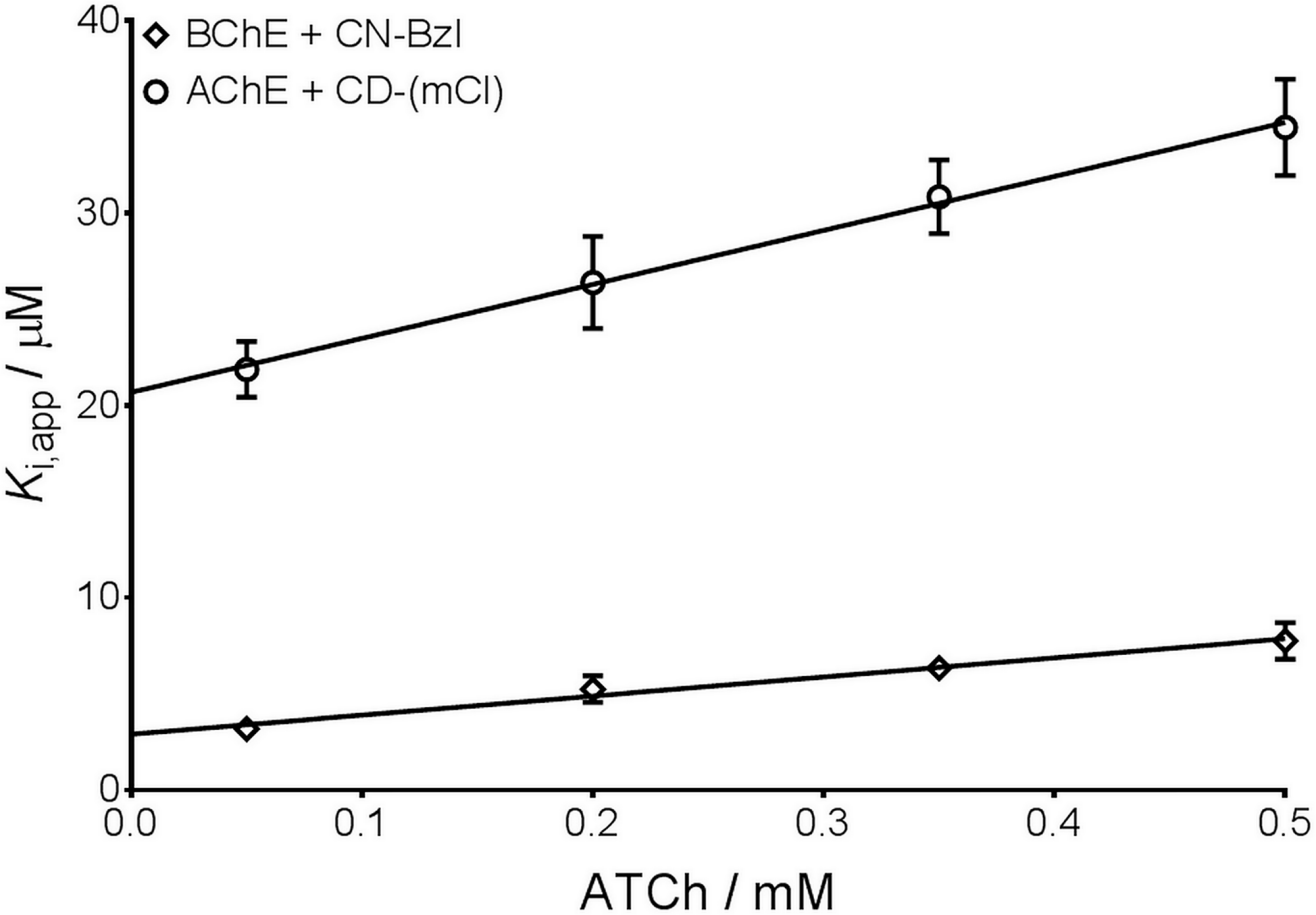
Representative inhibition experiment of BChE inhibited by CN-Bzl and AChE inhibited by CD-(mCl). Points indicate the average apparent enzyme-inhibitor constant (*K*_*i*,*app*_) at a given substrate (acetylthiocholine; ATCh) concentration according to the Hunter-Downs equation. The lines derived from linear regression analysis and y-intercept represent the enzyme-inhibitor dissociation constant *K*_i_. The concentrations of CN-Bzl and CD-(mCl) used in experiments were 2–10 μM and 10–40 μM, respectively. For BChE and CN-Bzl determined enzyme-inhibitor dissociation constant (*K*_i_) and enzyme-substrate dissociation constant (*K*_s_) were 2.9±0.3 μM and 0.29±0.04 mM, respectively. For AChE and CD-(mCl) determined *K*_i_ and *K*_s_ constants were 23±1 μM and 0.74±0.08 mM.

All cinchonidines and cinchonines reversibly inhibited the activity of both BChE and AChE, forming noncovalent interactions within the active site of the enzymes. To measure the inhibition potency of the tested compounds, we determined the dissociation constants (± standard errors) of the enzyme-inhibitor complex (*K*_i_) and the dissociation constants of the enzyme-substrate complex (*K*_s_) (Fig 3). The *K*_i_ and *K*_s_ constants for BChE and AChE, as well as the used concentration range of inhibitors, are given in Table 1.

**Table 1:** Inhibition of human BChE and AChE by the tested compounds.

Compound	BChE			AChE			*K*_i_(AChE)/ *K*_i_ (BChE)
	*c(I)/μM*	*K*_i_/μM	*K*_s_/mM	*c(I)/ μM*	*K*_i_/*μ*M	*K*_s_/mM	
CD	*10–200*	28±4 (c)	0.25±0.05	*100–200*	>400	-	~14
CN	*2–20*	4.9±1.4 (c)	0.50±0.27	*20–100*	34±1 (c)	0.25±0.01	12
CD-Met	10–50	26±4 (c)	0.70±0.22	50–200	67±6 (c)	0.70±0.17	2.6
CN-Met	*10–80*	29±4(c)	0.35±0.07	*20–200*	42±4 (c)	0.32±0.05	1.5
CD-Bzl	*0*.*05–0*.*5*	0.075±0.007(c)	0.27±0.03	*10–50*	15±2 (m)	0.78±0.3	200
CN-Bzl	*2–10*	2.9±0.3 (c)	0.29±0.04	*50–200*	121±12 (m)	1.1±0.4	42
CD-(pBr)	*0*.*02–0*.*20*	0.038±0.005 (c)	0.34±0.07	*10–50*	19±1 (m)	2.2±0.524	510
CN-(pBr)	*2–10*	3.5±0.3 (c)	0.44±0.07	*20–100*	31±1 (m)	2.7±0.9	8.8
CD-(pCH_3_)	*0*.*10–0*.*50*	0.17±0.02 (m)	0.38±0.06	*10–50*	16±1 (c)	1.3±0.2	99
CN-(pCH_3_)	*2–10*	3.1±0.3 (c)	0.33±0.05	*20–100*	42±2 (c)	2.0±0.2	13
CD-(pNO_2_)	*5–20*	6.0±0.5 (c)	0.38±0.05	*20–200*	36±2 (n)	-	5.9
CN-(pNO_2_)	*5–20*	7.6±0.7 (c)	0.32±0.04	*20–100*	51±2 (n)	-	6.6
CD-(pCl)	*0*.*05–0*.*20*	0.10±0.01 (c)	0.42±0.05	*20–80*	37±4 (n)	-	350
CN-(pCl)	*5–20*	6.2±0.8 (m)	0.49±0.12	*10–80*	40±4 (n)	-	6.4
CD-(mBr)	*0*.*25–1*	0.60±0.03 (m)	0.50±0.05	*10–40*	20±1 (m)	0.91±0.08	33
CN-(mBr)	*1–5*	2.3±0.2 (m)	0.48±0.09	*20–80*	39±2 (m)	0.73±0.08	17
CD-(mCH_3_)	*0*.*05–0*.*20*	0.077±0.006 (m)	0.52±0.08	*3–10*	3.0±0.3 (m)	0.63±0.14	39
CN-(mCH_3_)	*2–8*	4.6±0.4 (m)	0.53±0.00	*40–80*	49±2 (m)	0.66±0.06	11
CD-(mNO_2_)	*2–20*	4.7±0.4(m)	0.62±0.1	*1–5*	2.5±0.2 (c)	1.0±0.2	0.53
CN-(mNO_2_)	*2–40*	4.2±0.3 (m)	0.35±0.04	*2–20*	8.6±0.5 (m)	0.72±0.10	2.1
CD-(mCl)	*0*.*2–0*.*6*	0.24±0.01 (m)	0.53±0.05	*10–40*	23±1 (c)	0.74±0.08	95
CN-(mCl)	*5–20*	5.4±0.5 (m)	0.63±0.12	*50–160*	56±8 (c)	0.19±0.03	10
Ethopropazine [31]	*0*.*25–2*	0.16±0.03	0.69±0.14	*200*	161	0.10	1010
Donepezil [40, 41]		2.3 ± 1.0			0.0043		0.0019

c, n and m stands for competitive, noncompetitive and mixed type of inhibition. The selectivity of the corresponding compound is determined as the ratio of *K*_i_ constants for AChE and BChE and the corresponding compound.

BChE activity was inhibited by all compounds with *K*_i_ constants ranging from 0.038–29 μM (Table 1). Interestingly, all CD derivatives displayed higher affinities (1/*K*_i_) than the parent CD compound, while affinities of CN derivatives were almost unchanged from in comparison to their parent compound CN. BChE displayed the highest affinity toward compound CD-(pBr), followed by CD-Bzl, CD-(mCH_3_) and CD-(pCl), all with *K*_i_ values up to 100 nM, which classifies them as high potent BChE inhibitors [31]. Moreover, their affinities are slightly higher than that of the potent BChE inhibitor, ethopropazine [31]. BChE showed the lowest affinity toward compounds CD-Met and CN-Met; approximately 730 times compared to CD-(pBr). Since both CD-Met and CN-Met are the only compounds with an aliphatic substituent on the nitrogen atom of the quinuclidinium part of the molecule, it seems that the size and electronic properties of the substituent at that position are important for achieving high inhibition potency. This observation is in accordance with results by Nawaz et al. [22], where cinchonine quaternized with anthracene was about a 110 times more potent inhibitor than cinchonine without a substituent. The inhibition potency of CD-Met toward human BChE determined here is similar to that for equine BChE determined previously [23]. The *K*_s_ values derived from the kinetics of inhibition were very close to BChE’s previously determined Michaelis-Menten constant (*K*_M_) [7], which implies binding of the tested compounds to the catalytic site of BChE. The inhibition by all of the tested cinchonidines and cinchonines was competitive up to 0.35 mM substrate because the apparent dissociation constants proportionally depended on the substrate concentration as shown in Fig 3. At the substrate concentrations higher than 0.35 mM a slight deviation from linearity in the Hunter-Downs plot was observed for compounds with *meta*-positioned substituents on the benzene ring, CD-(pCH_3_) and CN-(pCl) indicating mixed inhibition (Table 1).

For BChE, the impact of changes in substituents on the benzene ring on inhibition potency can be analysed separately for cinchonidines and cinchonines. The inhibition potency of cinchonines with a benzene ring seems to be unaffected by the size and position of the substituents on the benzene ring, displaying only a 3–14 times more potent inhibition than CD-Met, which has no such substitution. On the other hand, the inhibition potency of cinchonidines toward BChE increased 44–700 times compared to that of CD-Met by introducing substituents at the benzene ring as in CD-(mBr) and CD-(pBr), respectively. Only cinchonidines with a nitro group on the benzene ring, CD-(pNO_2_) and CD-(mNO_2_), displayed an inhibition potency toward BChE only five times higher than CD-Met. Furthermore, no particular (up to 2.2 times) binding preference of BChE neither for cinchonidines nor for cinchonines regarding *meta-* or *para-*positioned substituents on the benzene ring was detected. The only exception was a 16 times higher preference for *para*-substituted cinchonidine with a bromine atom on the benzene ring CD-(pBr) compared to that in *meta* position CD-(mBr).

All compounds inhibited AChE with *K*_i_ constants ranging from 2.5–400 μM (Table 1) which is at least 1000 times lower than the affinity of donepezil [40, 41]. Similarly to BChE, all of the CD derivatives displayed higher affinities (1/*K*_i_) than the parent CD compound, while affinities of CN derivatives were almost unchanged from their parent compound CN. AChE had the highest affinity for CD-(mNO_2_) and CD-(mCH_3_), while the lowest—about 160 or 50 times–displayed for CD or CN-Bzl, respectively. It seems that the affinity of AChE toward the tested compounds was not affected by the size of the substituent at the nitrogen atom on the quinuclidinium part of the molecule. Furthermore, AChE did not show particular preference either to a *meta* or a *para* orientation of substituents on the benzene ring in both series, cinchonidines or cinchonines. The exceptions were compounds with nitro or methyl substituents in which AChE preferred a *meta* over a *para* orientation (5.5 to 14-fold higher inhibition with compounds having *meta* substituted benzyl moiety). The value of *K*_s_ constants derived from the kinetics of inhibition was between the two enzyme-substrate dissociation constants calculated in the absence of an inhibitor [7], generally indicating the mixed type of AChE inhibition. To designate whether the tested compounds bind to catalytic, peripheral or another intermediate, more rigorous criteria as well experimental method (i.e. stopped-flow method) should be applied [42]. Noncompetitive inhibition was observed for *para*-substituted compounds with chlorine and nitro group on a benzene ring.

### AChE/BChE selectivity

The inhibition selectivity of the newly synthesised compounds was defined with the ratio of *K*_i_ constants determined for interaction with AChE and BChE (Table 1). Overall, all of the compounds had a higher preference for BChE, among which five compounds displayed a 95–510 times higher inhibition selectivity toward BChE over AChE. The most selective BChE inhibitor was CD-(pBr), followed by CD-(pCl) and CD-Bzl, whose affinities were 510, 350 and 200 times higher than that for AChE, respectively. The lowest inhibition selectivity, only up to 2.6 times, was obtained by compounds with aliphatic substituents on the quinuclidine nitrogen of the molecule (CD-Met and CN-Met) and compound with a nitro group in the meta position on the benzene ring (CD-(mNO2). It is worth mentioning that the BChE selectivity of CD-(pBr), CD-(pCl) and CD-Bzl is similar to that of tacrine-based inhibitors and phenothiazine ethopropazine currently in use to treat parkinsonism [43].

### Cholinesterase stereoselectivity

The stereoselectivity of both cholinesterases was evaluated as a ratio of *K*_i_ constants determined for cinchonidines and their corresponding *pseudo*-enantiomers cinchonines (Table 2). Overall, the stereoselectivity of both enzymes ranged from an insignificant (up to 1.6) to a 92 times higher preference to bind cinchonidines than the corresponding cinchonines. BChE showed an about 4–92 times higher stereoselectivity to bind cinchonidines compared to corresponding cinchonines for all cinchonidine-cinchonine pairs except for compounds with an alkyl substituent on the quinuclidinium nitrogen and compounds with a nitro group attached to the benzene ring (pNO_2_ and mNO_2_ compounds). BChE displayed the highest stereoselectivity in the case of CD-(pBr) with a 92 times higher preference compared to that of the corresponding CN *pseudo*-enantiomer. By contrast, the highest AChE stereoselectivity was shown in the case of compounds with the methyl group in *meta* position on the benzene ring, where AChE had a 16 times higher affinity to CD-(mCH_3_) compared to CN-(mCH_3_). Generally, AChE stereoselectivity was lower than that of BChE; the highest difference was for compounds with a bromide or chloride group in *para* position, where AChE stereoselectivity was about 57 times lower than that of BChE.

**Table 2:** Stereoselectivity of BChE and AChE.

	*K*_i(CN/CD)_									
	Met	Bzl	(pBr)	(pCH_3_)	(pNO_2_)	(pCl)	(mBr)	(mCH_3_)	(mCl)	(mNO_2_)
BChE	1.1	39	92	19	1.3	60	3.9	60	23	0.89
AChE	0.62	8.1	1.6	2.5	1.4	1.1	2.0	16	2.5	3.5

Stereoselectivity was determined as a ratio of *K*_i_ constants for cinchonidines and their corresponding *pseudo*-enantiomeric cinchonine pairs.

### Docking study and quantum-chemical calculations

**Fig 4:**
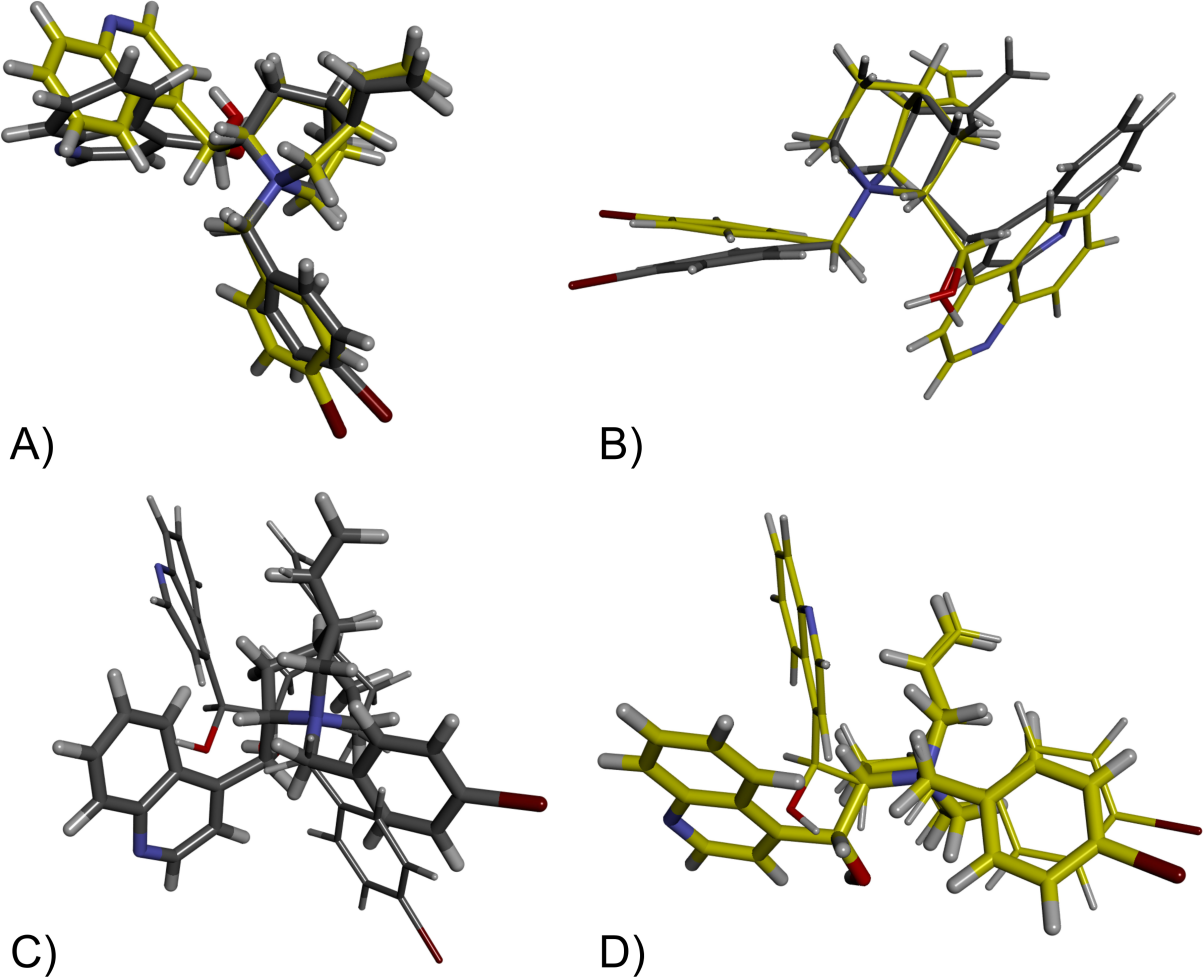
CD-(pBr) (thicker stick model) and CN-(pBr) (slimmer stick model) in the active site of BChE (yellow carbon atoms) and AChE (grey carbon atoms) obtained by ONIOM calculations. A) overlay of CD-(pBr) bioactive conformers; B) overlay of CN-(pBr) bioactive conformers; C) overlay of CD-(pBr) and CN-(pBr) bioactive conformers from AChE; D) overlay of CD-(pBr) and CN-(pBr) bioactive conformers from BChE.

To propose the key interactions for compounds within the active sites, molecular docking studies were performed, and to give us better insight into multiple interactions and structural requirements for inhibition of *pseudo*-enantiomers, Quantum mechanical docking calculations were performed [35] followed by combined, QM/QM 2-layer ONIOM calculations for CD-(pBr) and CN-(pBr) [38, 39]. The resulting geometries were analysed based on their energy values and the overlay of CD-(pBr) and that of CN-(pBr) bioactive conformers (the lowest energy conformers) from both enzymes are presented in Fig 4. It can be seen that the main difference in binding of cinchonidine derivative in AChE and BChE is the orientation of the quinoline group (tetrahedral angle in BChE C8-C9-Q4'-Q3' 109°; in AChE C8-C9-Q4'-Q3' -170°) while the rest of the moieties are positioned very similar (Fig 4A)). These sterical differences in placement are the most probable reason for the observed differences in the stereoselectivity of BChE and AChE. Furthermore, the overlay of bioactive conformers of the *Cinchona* derivative from both enzymes showed similarity concerning the positioning of almost all moieties except the vinyl group (tetrahedral angle in BChE H3-C3-C10-H10' 178°; in AChE H3-C3-C10-H10' 93°) (Fig 4B)). On the other hand, the overlay of geometries of each *pseudo*-enantiomer from AChE (Fig 4C)) and BChE (Fig 4D)) revealed that the positioning of hydroxyl and quinoline group are those which due to the C8, C9 opposite chirality have to bind significantly different. The differences in bioactive conformers from AChE are the greatest which can be associated with the smaller active site of that enzyme.

**Fig 5:**
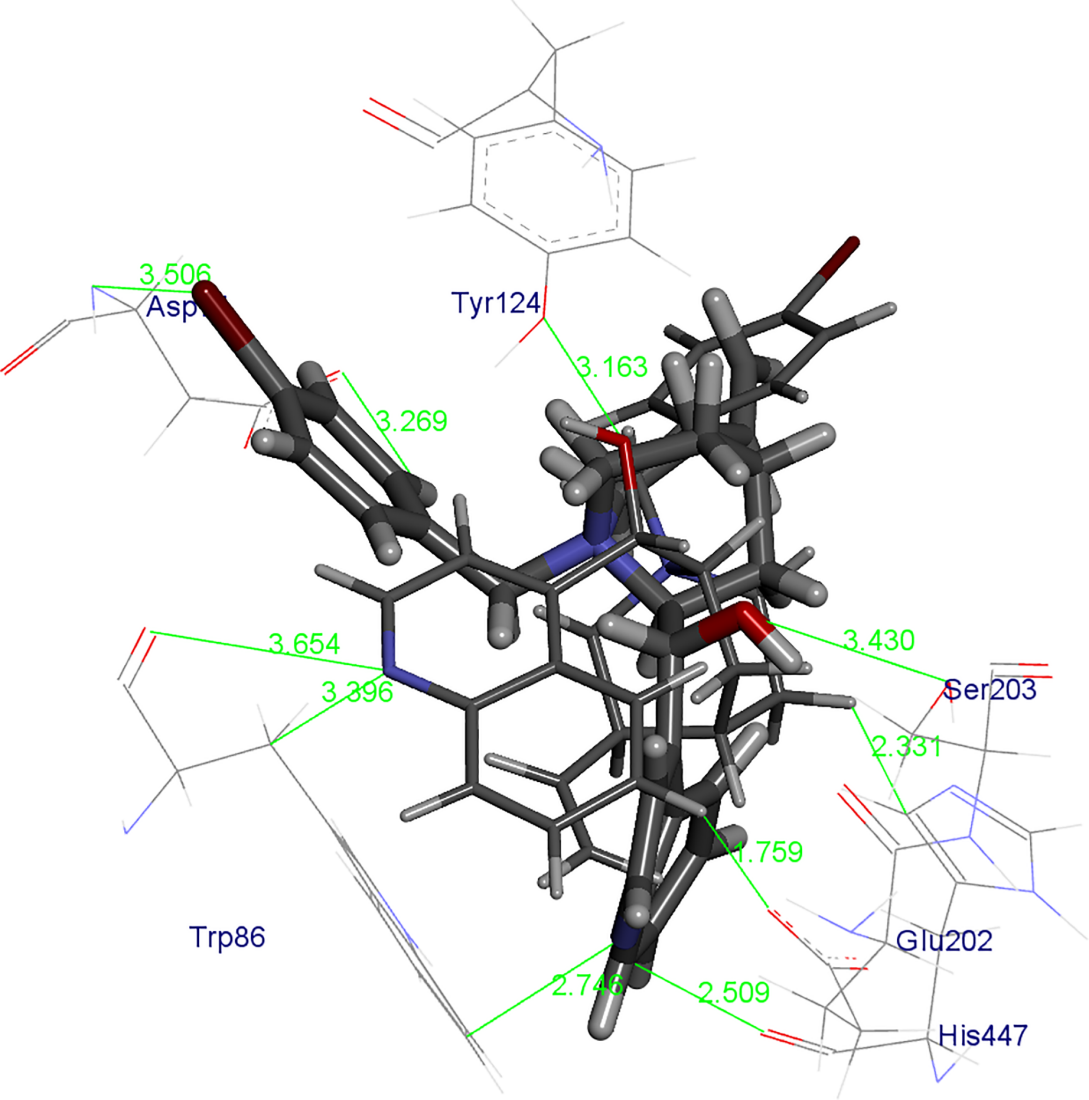
CD-(pBr) (stick model) and CN-(pBr) (yellow stick model) in the active site of BChE obtained by ONIOM calculations. The hydroxyl group hydrogen bond is marked with a green line and the values are given in Å. Only amino acids in the vicinity (up to 5 Å) of the compounds are displayed. Hydrogen atoms of the amino acid are omitted for clarity.

Generally, kinetic studies pointed out that BChE and AChE can more effectively accommodate cinchonidines inside the active site gorge. Modelling experiments revealed steric and electronic reasons for the measured affinities. Interactions in the BChE active site responsible for stabilization of enzyme complexes with CD-(pBr) and CN-(pBr) are pointed out in Fig 5.

One of the crucial stabilization factors of compounds:BChE complexes are π-π interactions, thus aromatic moieties of the compounds are placed in the same regions of the active site of BChE. In the cation-π binding site there are π-π interactions between the quinoline moieties of *Cinhona* derivatives with Trp86 and the close contacts with His447 (catalytic triad amino acid). At the same time, the aromatic part of the benzyl group is positioned toward the PAS having π-π interactions with the Tyr332. Positioning of the aromatic parts of the compounds dictated the position of the vinyl group as well as the quinuclidine ring and most importantly the hydroxyl C9 group which, as expected made H-bonds. Therefore, stabilization is largely achieved by the existence of a very strong H-bond between the oxygen atom from C9 hydroxyl group of and Thr120 in CD-(pBr), In the complex of CN-(pBr) derivative with BChE, C9 hydroxyl group is pointed toward Tyr332 and is much longer.

**Fig 6:**
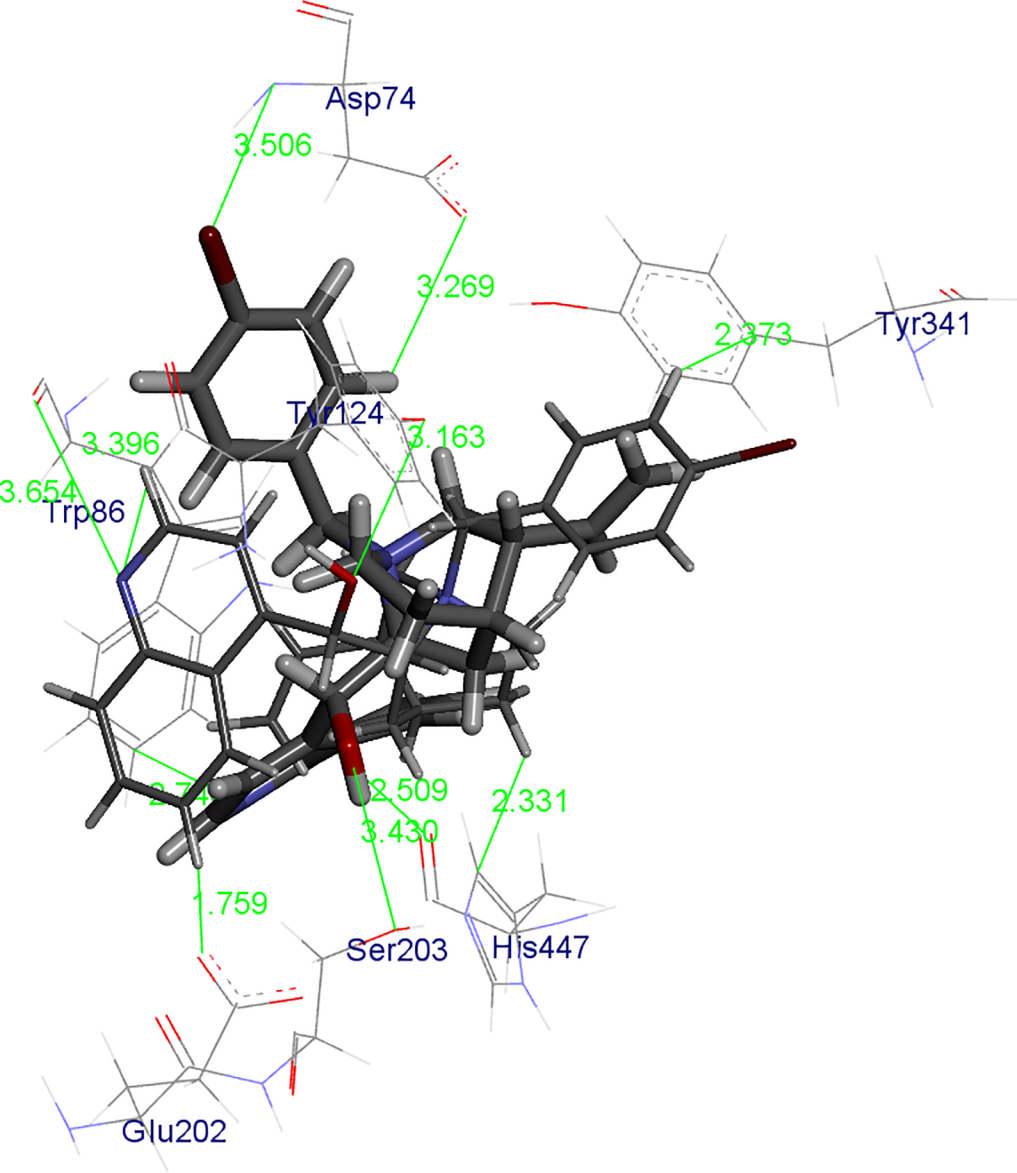
CD-(pBr) (stick model) and CN-(pBr) (yellow stick model) in the active site of AChE obtained by ONIOM calculations. The hydroxyl group hydrogen bond is marked with a green line and the values are given in Å. Only amino acids in the vicinity (up to 4 Å) of the compounds are displayed. Hydrogen atoms of amino acid are omitted for clarity.

Interactions in the AChE active site responsible for the stabilization of enzyme complexes with CD-(pBr) and CN-(pBr) are pointed out in Fig 6. In the AChE complexes with cinchonines and cinchonidines, the observed π-π interactions between benzyl and quinoline moieties are not in the same regions as the one for compounds in BChE since the active site is smaller and contains more aromatic amino acids. Therefore, the quinoline ring of CD-(pBr) has close contacts with Trp86 while the quinoline ring of CN-(pBr) is pointed toward Tyr133. The aromatic ring of benzyl moiety in CD-(pBr) is oriented toward the Asp74, but the same ring in CN-(pBr) toward Tyr341. H-bonds of the 9C hydroxyl groups present in both CD (Ser203) and CN (Tyr124) complexes and are longer than the one in the CD-(pBr):BChE complex.

## General discussion

Ever since the first cholinesterase inhibitors were officially approved for clinical treatment of AD and other similar neurological impairments, the number of studies focused on the search for compounds with AChE inhibition potency has greatly increased. Nowadays, as a result of very intensive and comprehensive studies related to AD, the search has expanded to developing BChE selective inhibitors, as well as AChE dual binding inhibitors, with a tendency toward these two qualities merging into the characteristics of one compound. With this in mind, we synthesized two series of ten synthetic derivatives whose primary structural motive was the alkaloid from the bark of the *Cinchona* tree, one cinchonidine and the second its *pseudo*-enantiomer cinchonine. The quinuclidinium nitrogen atom of all compounds was quaternized to gain a structural feature common to all good cholinesterase inhibitors: a positive charge that facilitate approach and entry into the cholinesterase active site. An additional benefit of that quaternization can be the fact that the 3D structures of the quaternized quinuclidinium compounds resemble that of acetylcholine, an AChE physiological substrate.

The kinetic results confirmed our expectations that the compounds would be reversible BChE and AChE inhibitors. Although all newly-synthesized compounds were more potent BChE inhibitors than AChE (except in the case of *para*-nitro substituted benzyl moiety), four cinchonidines were identified as candidates for BChE selective inhibitors with potency for further structural tuning. CD-(pBr), CD-(pCl), CD-Bzl and CD-(mCH_3_) were selected due to their inhibition potency (*K*_i_ constants in nanomolar range), which can be compared to the *K*_i_ of ethopropazine for human BChE (*K*_i_ = 0.16 μM) [31]. The results obtained from molecular modelling gave us better insight into the multiple interactions and structural requirements for the inhibitory properties of *pseudo*-enantiomers. Comparison of kinetic results with results of molecular modelling showed that, beside π-π interactions found to be important for the stabilization of BChE:cinchonidines complexes, a strong H-bond of the 9C hydroxyl group with Thr120, located between the choline binding site and PAS, is crucial for the determined stereoselectivity.

Regarding AChE, the inhibition potency of the tested compounds was much lower than for BChE. The inhibition potency of those compounds was up to 1000 times lower compared to compounds in the use as anti-AD drugs; galantamine (IC_50_ = 0.8 μM in human AChE), huperzine A (IC_50_ = 0.047 μM in human AChE) and donepezil (IC_50_ = 0.038 nM in hAChE) [2, 15, 43]. The inhibition profile of the tested alkaloids and docking study imply that they create interactions in both PAS and residues deeper in the AChE active site, the mode of binding, which can be compared with the binding of donepezil [44].

The treatment of AD is focused on slowing down the disease’s progression and symptomatic treatment, maintaining functional status and improving the patient’s quality of life. So far the most successful approach in treating AD has been the use of cholinesterase inhibitors that target primary AChE [12, 13]. Recent studies demonstrated that over the course of AD’s progression, the activity of AChE in certain brain regions decreases to only 10–15% of its normal values, while BChE activity progressively increases to a maximum of 120% [2, 19]. Also, the increasing activity of BChE in neurotoxic plaques seen in AD suggests that BChE participates in the transformation of amyloid plaques from an initially benign form as those in normal aging to a malignant form as in neurotoxic plaques seen in an AD-affected brain [19]. Based on this it can be assumed that the here tested alkaloids with demonstrated BChE selectivity can affect the symptomatic treatment of AD by reducing the activity of BChE thus contributing to the increase of acetylcholine concentration and additionally to lowering the formation of neurotoxic plaques. The potential of the tested compounds to be considered as central nervous system drugs depends mostly on its ability to cross the blood-brain barrier. In the case of the synthesised here, the existence of a quaternary amine in the structure makes these compounds permanently positively charged and is therefore considered to be less able to penetrate the blood brain barrier by passive transport. However, it has been shown experimentally determined that positively charged quinolinium derivatives can enter membranes [45, 46].

## Conclusion

In this study, we reported the synthesis of twenty synthetic quaternary derivatives of *Cinchona* alkaloids which presented ten *pseudo*-enantiomeric pairs. A comprehensive evaluation of BChE and AChE inhibition potency accompanied with a docking study enabled the identification of BChE selective inhibitors from which cinconidine CD-(pBr) can be pointed out as a lead molecule for further optimization for possible use in the treatment of neurodegenerative diseases, like Alzheimer’s disease. Since the existence of a quaternary ammonium group in the structure makes it permanently positively charged and less able to penetrate the blood brain barrier (BBB) by passive transport, the possible use and further structure and inhibition profile refinement of studied compounds, particularly the nonselective compounds can be oriented toward the development of peripherally active cholinesterase inhibitors, which is the primary treatment in early stages and mild forms of *myasthenia gravis* [47].

## Supporting information

S1 File A detailed description of the purity, synthesis and analysis for all compounds. (PDF)
