# Supplementary material for: Design and evaluation of selective butyrylcholinesterase inhibitors based on Cinchona alkaloid scaffold
Source: PLoS One. 2018 Oct 5;13(10):e0205193. doi: 10.1371/journal.pone.0205193 (PMC6173406; doi:10.1371/journal.pone.0205193)
Supplement: S1 File — (PDF) [file pone.0205193.s001.pdf]

Supporting Information for

Design and evaluation of selective butyrylcholinestrase inhibitors based on Cinchona alkaloid scaffold

Anita Bosak, Alma Ramić, Tamara Šmidlehner, Tomica Hrenar, Ines Primožič and Zrinka Kovarik

Contents

S1. Synthesis of compounds **1**

S1.1. General procedure for synthesis **2**

S1.2. Analytical data **2**

S2. 1H and 13C NMR spectra of prepared compounds **9**

Synthesis of compounds

Compounds **CD-(mBr), CD-(mMet), CD-(mCl), CD-(mNO$_2$) CN-(pMet), and CN-(mMet)** are novel compounds. Their spectral and analytical data are given in the manuscript. Melting points were determined on a Melting Point B-540 apparatus (Büchi, Essen, Germany) and are uncorrected. Optical rotations were measured on an Optical Activity AA-10 automatic polarimeter (Optical Activity Limited, Ramsey, Cambridgeshire, UK) at 22 °C. CHN analysis was performed on Perkin Elmer 2400 Series II CHNS analyzer. 1D and 2D, ^{1}H and ^{13}CNMR spectra were recorded on a Varian XL-GEM 600 spectrometer at 22 °C and Bruker Avance III HD 400 MHz/54 mm Ascend spectrometer (Bruker Optics Inc, Billerica, MA, USA). Chemical shifts are given in ppm downfield from tetramethylsilane (TMS) as an internal standard and coupling constants (*J*) in Hz. Splitting patterns are designated as s (singlet), d (doublet), dd (doublet of doublets), ddd (doublet of doublet of doublets), qd (quartet of doublets), t (triplet), q (quartet) or m (multiplet). Quinoline hydrogen and carbon atoms are marked with an apostrophe. Benzene hydrogen and carbon atoms are marked with an double apostrophe.

S1.1. General procedure for synthesis

The compounds were synthesized following this procedure: *Cinchona* alkaloid (1 mmol) and appropriate halide (1.05 mmol for *para*- and 1.20 mmol for *meta*-substituted halides) were heated to reflux in toluene (5 mL). End of reaction was detected with thin layer chromatography ($CHCl_3$: MeOH = 9:1). After cooling to room temperature, the precipitated product was isolated by filtration and

recrystallized from stated solvent. *Meta*-substituted derivatives required longer time for reaction (24 – 48 h) then *para*-substituted derivatives (1 – 2 h).

S1.2. Analytical data

***N*-methyl cinchonidinium iodide (CD-Met).** Orange solid. Yield: 69% (recrystallized from methanol/diethyl ether). m.p. 222.5 °C; $[\alpha]_D^{24} = -214° \ (c\ 0.1, \mathrm{MeOH})$; IR (cm^{-1}): 3443 (O-H), 3172 (C-H$_{Ar}$), 1511 (C=N), 1132 (C-N); Anal. Calc. for [$C_{20}H_{25}IN_2O$]: C, 55.05; H, 5.78; N, 6.42 found C, 55.08; H, 5.77; N, 6.40; ^{1}H NMR (600 MHz, DMSO-*d*$_6$) δ ppm 1.20 - 1.27 (1 H, m, H7b) 1.90 - 1.97 (1 H, m, H4) 2.02 - 2.13 (3 H, m, H7a, H5) 2.78 - 2.84 (1 H, m, H3) 3.42 (3 H, s, CH_3) 3.43 - 3.47 (1 H, m, H6b) 3.64 - 3.77 (3 H, m, H8, H2) 4.16 - 4.22 (1 H, m, H6a) 4.96 (1 H, d, *J*=10.6 Hz, H11b) 5.15 (1 H, d, *J*=17.2 Hz, H11a) 5.69 (1 H, ddd, *J*=17.2, 10.6, 6.6 Hz, H10) 6.21 (1 H, m, H9) 6.58 (1 H, d, *J*=3.7 Hz, OH) 7.70 - 7.74 (1 H, m, H7’) 7.75 (1 H, d, *J*=4.4 Hz, H3’) 7.81 - 7.86 (1 H, m, H6’) 8.08 - 8.11 (1 H, m, H5’) 8.15 (1 H, d, *J*=8.4 Hz, H8’) 8.96 (1 H, d, *J*=4.4 Hz, H2’); ^{13}C NMR (151 MHz, DMSO-*d*$_6$) δ ppm 19.8 (C7) 24.7 (C5) 25.7 (C4) 37.4 (C3) 48.5 (CH_3) 54.5 (C6) 63.7 (C2) 64.3 (C9) 66.4 (C8) 116.3 (C11) 119.8 (C3’) 123.3 (C5’) 124.2 (C9’) 127.2 (C6’) 129.4 (C7’) 129.9 (C8’) 138.1 (C10) 145.2 (C10’) 147.6 (C4’) 150.1 (C2’).

***N*-benzylcinchonidinium bromide (CD-Bzl).** White solid. Yield: 90% (recrystallized from methanol/diethyl). m.p. 185.6-186.7 °C; $[\alpha]_D^{24} = -155° \ (c\ 0.1, \mathrm{MeOH})$. IR (cm^{-1}): 3435 (O-H), 3115 (C-H$_{Ar}$), 1506 (C=N), 1129 (C-N); Anal. Calc. for [$C_{26}H_{29}BrN_2O$]: C, 67.10; H, 6.28; N, 6.02 found C, 67.13; H, 6.27; N, 6.04; ^{1}H NMR (300 MHz, DMSO-*d*$_6$) δ ppm 1.20 - 1.37 (1 H, m, H7b) 1.82 (1 H, m, H5b) 1.96 - 2.21 (3 H, m, H5a, H7a, H4) 2.69 (1 H, m, H3) 3.18 - 3.32 (2 H, m, H2b, H6b) 3.78 (1 H, d, *J*=11.3 Hz, H2a) 3.94 (1 H, t, *J*=8.7 Hz, H8) 4.29 (1 H, t, *J*=10.6 Hz, H6a) 4.90 - 5.10 (2 H, m, H11) 5.10 - 5.25 (2 H, m, CH_2) 5.68 (1 H, ddd, *J*=17.2, 10.6, 6.5 Hz, H10) 6.52 - 6.63 (1 H, m, H9) 6.75 (1 H, d, *J*=4.5 Hz, OH) 7.54 - 7.66 (3 H, m, H4’’, H6’’, H2’’) 7.70 - 7.91 (5H, m, H6’, H3’’’, H5’’, H7’, H3’) 8.08 - 8.15 (1 H, m, H5’) 8.31 (1 H, d, *J*=8.3 Hz, H8’) 8.99 (1 H, d, *J*=4.5 Hz, H2’); ^{13}C NMR (75 MHz, DMSO-*d*$_6$) δ ppm 21.4 (C7) 24.7 (C5) 26.3 (C4) 37.3 (C3) 51.1 (C6) 59.7 (C2) 63.2 (CH2) 64.5 (C9) 68.1 (C8) 116.8 (C11) 120.6 (C3’) 124.2 (C5’) 124.8 (C9’) 127.7 (C6’) 128.4 (C1’’) 129.4 (C7’) 129.9 (C8’) 130.3 (C2’’, C6’’) 130.6 (C4’’) 134.2 (C3’’, C5’’) 138.6 (C10) 145.7 (C10’) 148.1 (C4’) 150.6 (C2’).

***N*-(4-bromobenzyl)cinchonidinium bromide (CD-(pBr)).** White solid. Yield: 80% (recrystallized from methanol/diethyl). m.p. 234.9-235.8 °C; $[\alpha]_D^{24} = -176° \ (c\ 0.1, \mathrm{MeOH})$; IR (cm^{-1}): 3436 (O-H), 3144 (C-H$_{Ar}$), 1506 (C=N), 1071 (C-N); Anal. Calc. for [$C_{26}H_{28}Br_2N_2O$]: C, 57.37; H, 5.19; N, 5.15 found C, 57.33; H, 5.17; N, 5.14; ^{1}H NMR (600 MHz, DMSO-*d*$_6$) δ ppm 1.20 - 1.36 (1 H, m, H7b) 1.80 (1 H, t, *J*=9.4 Hz, H5b) 1.93 - 2.02 (1 H, m, H4) 2.02 - 2.08 (1 H, m, H5a) 2.12 (1 H, dd, *J*=12.8, 8.1 Hz, H7a) 2.67 (1 H, m, H3) 3.23 (1 H, td, *J*=11.4, 4.4 Hz, H6b) 3.32 - 3.35 (1 H, m, H2b) 3.61 - 3.76 (1 H, m, H2a) 3.89 (1 H, t, *J*=9.0 Hz, H8) 4.17 - 4.30 (1 H, m, H6a) 4.95 (1 H, d, *J*=10.6 Hz,

H11b) 4.99 (1 H, d, *J*=12.5 Hz, H11a) 5.11 - 5.17 (2 H, m, CH_2) 5.67 (1 H, ddd, *J*=17.2, 10.6, 6.0 Hz, H10) 6.48 - 6.59 (1 H, m, H9) 6.72 (1 H, d, *J*=4.4 Hz, OH) 7.68 (2 H, d, *J*=8.4 Hz, H2'', H6'') 7.75 (1 H, td, *J*=7.6, 1.3 Hz, H3') 7.78 - 7.81 (3 H, m, H6', H5'', H3'') 7.83 - 7.87 (1 H, m, H7') 8.09 - 8.13 (1 H, m, H5') 8.28 (1 H, d, *J*=8.1 Hz, H8') 8.98 (1 H, d, *J*=4.4 Hz, H2'), ^{13}C NMR (151 MHz, DMSO-d_6) δ ppm 20.8 (C7) 24.2 (C5) 25.8 (C4) 36.9 (C3) 50.6 (C6) 59.1 (C2) 61.9 (CH_2) 64.1 (C9) 67.7 (C8) 116.3 (C11) 120.0 (C3') 123.5 (C5') 124.0 (C9') 124.3 (C4'') 127.1 (C6') 127.3 (CI) 129.4 (C7') 129.9 (C8') 131.9 (C5'', C3'') 135.8 (C6'', C2'') 138.0 (C10) 145.2 (C10') 147.6 (C4') 150.1 (C2').

***N*-(4-methylbenzyl)cinchonidinium bromide (CD-(pMet)).** Pink solid. Yield: 72% (recrystallized from methanol/diethyl). m.p. 218.3-219.8 °C; $[\alpha]_D^{24} = -226° \; (c\ 0.1, \mathrm{MeOH})$; IR (cm^{-1}): 3422 (O-H), 3120 (C-H_{Ar}), 1506 (C=N), 1127 (C-N); Anal. Calc. for [$C_{27}H_{31}BrN_2O$]: C, 67.64; H, 6.52; N, 5.84 found C, 67.66; H, 6.51; N, 5.86; ^{1}H NMR (300 MHz, DMSO-d_6) δ ppm 1.21 - 1.35 (1 H, m, H7b) 1.73- 1.89 (1 H, m, H5b) 1.99 (1 H, m, H4) 2.03 - 2.18 (2 H, m, H7a, H5a) 2.40 (3 H, s, CH_3) 2.68 (1 H, s, H3) 3.14 - 3.30 (2 H, m, H2b, H6b) 3.68 (1 H, d, *J*=12.8 Hz, H2a) 3.89 (1 H, t, *J*=8.8 Hz, H8) 4.22 (1 H, t, *J*=11.7 Hz, H6a) 4.88 - 5.00 (2 H, m, H11) 5.01 - 5.19 (2 H, m, CH_2) 5.67 (1 H, ddd, *J*=17.2, 10.5, 6.6 Hz) 6.55 (1 H, s, H9) 6.72 (1 H, d, *J*=4.1 Hz, OH) 7.39 (2 H, m, *J*=7.9 Hz, H5'', H3'') 7.59 (2 H, m, *J*=7.9 Hz, H2'', H6'') 7.70 - 7.91 (3 H, m, H7', H3', H6') 8.06 - 8.15 (1 H, m, H5') 8.27 (1 H, d, *J*=8.3 Hz, H8') 8.98 (1 H, d, *J*=4.1 Hz, H2'); ^{13}C NMR (75 MHz, DMSO-d_6) δ ppm 21.3 (CH_3) 21.8 (C7) 24.7 (C5) 26.3 (C4) 37.3 (C3) 59.6 (C6) 62.3 (C2) 63.2 (CH_2) 64.6 (C9) 68.00 (C8) 116.8 (C11) 120.5 (C3') 124.1 (C5') 124.7 (C9') 125.3 (C1'') 127.6 (C6') 130.0 (C2'', C4'') 130.4 (C8') 134.1 (C3'', C5'') 138.6 (C10) 140.3 (C4'') 145.7 (C10') 148.1 (C4') 150.7 (C2').

***N*-(4-nitrobenzyl)cinchonidinium bromide (CD-(pNO_2)).** Yellow solid. Yield: 68% (recrystallized from methanol/diethyl). m.p. 193.5-194.5 °C; $[\alpha]_D^{24} = -317° \; (c\ 0.1, \mathrm{MeOH})$; IR(cm^{-1}): 3433 (O-H), 3176 (C-H_{Ar}), 1509 (C=N), 1519 (N-O), 1346 (N-O), 1113 (C-N); Anal. Calc. for [$C_{26}H_{28}BrN_3O_3$]: C, 61.18; H, 5.53; N, 8.23 found C, 61.17; H, 5.57; N, 8.20; ^{1}H NMR (600 MHz, DMSO-d_6) δ ppm 1.26 - 1.37 (1 H, m, H7b) 1.76 - 1.86 (1 H, m, H5b) 1.97 - 2.05 (1 H, m, H4) 2.05 - 2.12 (1 H, m, H5a) 2.12 - 2.18 (1 H, m, H7a) 2.65 (1 H, s, H3) 3.26 (1 H, td, *J*=11.6, 5.1 Hz, H6b) 3.34 - 3.38 (1 H, m, H2b) 3.81 - 3.87 (1 H, m, H2a) 3.97 (1 H, t, *J*=8.8 Hz, H8) 4.30 - 4.38 (1 H, m, H6a) 4.97 (1 H, d, *J*=10.6 Hz, H11a) 5.15 - 5.25 (2 H, m, CH_2) 5.33 (1 H, d, *J*=12.1 Hz, H11b) 5.68 (1 H, ddd, *J*=17.2, 10.6, 6.6 Hz, H10) 6.54 - 6.61 (1 H, m, H9) 6.76 (1 H, d, *J*=4.4 Hz, OH) 7.74 - 7.79 (1 H, m, H6') 7.81 - 7.88 (2 H, m, H3', H7') 8.07 (2 H, m, *J*=8.4 Hz, H2'', H6'') 8.12 (1 H, d, *J*=8.4 Hz, H5') 8.32 (1 H, d, *J*=8.4 Hz, H8') 8.43 (2 H, m, *J*=8.4 Hz, H5'', H3'') 9.00 (1 H, d, *J*=4.4 Hz, H2'); ^{13}C NMR (151 MHz, DMSO-d_6) δ ppm 20.9 (C7) 24.2 (C5) 25.7 (C4) 36.9 (C3) 50.9 (C6) 59.3 (C2) 61.4 (CH2) 64.1 (C9) 68.0 (C8) 116.4 (C11) 120.1 (C3') 123.6 (C5') 123.7 (C3'', C5'') 124.3 (C9') 127.20 (C6', C7') 129.4 (C5') 129.9 (C8') 135.3 (CI) 135.3 (C6'', C2'') 138.0 (C10) 145.1 (C4'') 147.6 (C10') 148.5 (C4') 150.2 (C2').

***N*-(4-chlorobenzyl)cinchonidinium bromide (CD-(pCl)).** White solid. Yield: 82%. m.p. 225.4-226.3 °C. $[\alpha]_D^{24} = -156°$ (c 0.1, MeOH); IR(cm^{-1}): 3430 (O-H), 3117 ($C-H_{Ar}$), 1506 (C=N), 1116 (C-N); Anal. Calc. for [$C_{26}H_{28}BrClN_2O$]: C, 62.47; H, 5.65; N, 5.60 found 62.44; H, 5.66; N, 5.63; ^1H NMR (300 MHz, DMSO-d_6) δ ppm 1.21 - 1.38 (1 H, m, H7b) 1.80 (1 H, t, J=9.2 Hz, H5b) 2.00 (1 H, m, H4) 2.06 - 2.20 (2 H, m, H7a, H5a) 2.67 (1 H, m, H3) 3.15 - 3.25 (1 H, m, H6b) 3.35 (1 H, d, J=11.1 Hz, H2b) 3.72 (1 H, d, J=13.0 Hz, H2a) 3.91 (1 H, t, J=8.8 Hz, H8) 4.14 - 4.34 (1 H, m, H6a) 4.92 - 5.08 (2 H, m, CH_2) 5.11 - 5.23 (2 H, m, H11) 5.67 (1 H, ddd, J=17.2, 10.6, 6.5 Hz, H10) 6.50 - 6.59 (1 H, m, H9) 6.71 (1 H, d, J=4.3 Hz, OH) 7.60 - 7.70 (2 H, m, H3', H6') 7.71 - 7.83 (4 H, m, ArH'') 7.84 - 7.88 (1 H, m, H7') 8.11 (1 H, d, J=8.5 Hz, H5') 8.28 (1 H, d, J=8.3 Hz, H8') 8.98 (1 H, d, J=4.5 Hz, H2'); ^{13}C NMR (75 MHz, DMSO-d_6) δ ppm 21.4 (C7) 24.7 (C5) 26.3 (C4) 37.4 (C3) 51.1 (C6) 59.7 (C2) 62.4 (CH2) 64.6 (C9) 68.24 (C8) 116.8 (C11) 120.5 (C3') 124.1 (C5') 124.7 (C9') 127.4 (CI) 127.7 (C6') 129.5 (C7') 129.9 (C8') 130.4 (C2'', C6'') 135.7 (C'') 136.0 (C3'', C5'') 138.5 (C10) 145.7 (C10') 148.1 (C4') 150.6 (C2').

***N*-methyl cinchoninium iodide (CN-Met).** Orange solid. Yield: 60% (recrystallized from acetonitrile/diethyl ether). m.p. 236.8-237.8 °C; $[\alpha]_D^{24} = +225°$ (c 0.1, MeOH); IR(cm^{-1}): 3446 (O-H), 3124 ($C-H_{Ar}$), 1511 (C=N), 1132 (C-N); Anal. Calc. for [$C_{20}H_{25}IN_2O$]: C, 55.05; H, 5.78; N, 6.42 found C, 55.02; H, 5.75; N, 6.40; ^1H NMR (600 MHz, DMSO-d_6) δ ppm 0.96 – 1.03 (1 H, m, H7b) 1.77 - 1.84 (1 H, m, H5b) 1.86 – 1.91 (1 H, m, H5a) 1.93 (1 H, m, H4) 2.20 (1 H, t, J=11.6 Hz, H7a) 2.78 (1 H, q, J=8.6 Hz, H3) 3.37 (3 H, s, CH_3) 3.49 - 3.60 (2 H, m, H6b) 3.66 (1 H, t, J=9.4 Hz, H2a) 3.74 (1 H, t, J=11.4 Hz, H8) 4.22 (1 H, ddd, J=11.9, 9.0, 2.6 Hz, H6a) 5.23 - 5.27 (2 H, m, H11) 6.01 (1 H, ddd, J=17.4, 10.1, 7.0 Hz, H10) 6.20 (1 H, s, H9) 6.66 (1 H, d, J=3.3 Hz, OH) 7.70 - 7.74 (1 H, m, H7') 7.76 (1 H, d, J=4.4 Hz, H3') 7.81 - 7.85 (1 H, m, H6') 8.10 (1 H, d, J=8.4 Hz, H5') 8.20 (1 H, d, J=8.1 Hz, H8') 8.97 (1 H, d, J=4.4 Hz, H2'); ^{13}C NMR (151 MHz, DMSO-d_6) δ ppm 19.6 (C7) 23.3 (C5) 26.2 (C4) 37.2 (C3) 48.0 (CH_3) 58.5 (C6) 59.9 (C2) 64.9 (C9) 65.7 (C8) 117.0 (C11) 119.8 (C3') 123.4 (C5') 124.2 (C9') 127.2 (C6') 129.4 (C7') 129.8 (C8') 137.0 (C10) 144.8 (C10) 147.6 (C4') 150.1 (C2').

***N*-benzyl cinchoninium bromide (CN-Bzl).** White solid. Yield: 61% (recrystallized from acetonitrile/diethyl ether); m.p. 259.3-260.5 °C; $[\alpha]_D^{24} = +136°$ (c 0.1, MeOH); IR(cm^{-1}): 3444 (O-H), 3187 ($C-H_{Ar}$), 1512 (C=N), 1071 (C-N); Anal. Calc. for [$C_{26}H_{29}BrN_2O$]: C, 67.10; H, 6.28; N, 6.02 found C, C, 67.12; H, 6.25; N, 6.01; ^1H NMR (300 MHz, DMSO-d_6) δ ppm 0.99 - 1.12 (1 H, m, H7b) 1.78 (2 H, d, J=8.3 Hz, H5) 1.87 (1 H, m, H4) 2.30 (1 H, t, J=11.5 Hz, H7a) 2.65 (1 H, q, J=8.0 Hz, H3) 2.88 - 3.03 (1 H, m, H2b) 3.48 (1 H, t, J=11.2 Hz, H6b) 3.88 - 4.06 (2 H, m, H2a, H8) 4.24 (1 H, t, J=9.4 Hz, H6a) 4.98 (1 H, d, J=12.3 Hz, CH2) 5.14 (1 H, d, J=12.3 Hz, CH_2) 5.18 - 5.29 (2 H, m, H11) 5.93 - 6.08 (1 H, m, H10) 6.53 (1 H, s, H9) 6.82 (1 H, d, J=3.9 Hz, OH) 7.55 - 7.62 (3 H, m, H3', H6', H7') 7.71 - 7.89 (5 H, m, Ar H'') 8.11 (1 H, d, J=8.3 Hz, H5') 8.36 (1 H, d, J=8.3 Hz, H8') 8.99 (1 H, d, J=4.3 Hz, H2'); ^{13}C NMR (75 MHz, DMSO-d_6) δ ppm 21.2 (C7) 23.4 (C5) 26.8 (C4)

37.1 (C3) 54.3 (C6) 56.5 (C2) 62.8 (C5) 65.2 (C9) 67.6 (C8) 117.5 (C11) 120.6 (C3’) 124.4 (C5’) 124.9 (C9’) 127.7 (C6’) 128.4 (C1’’) 129.4 (C3’’, C5’’) 129.9 (C7’) 130.3 (C8’) 130.6 (C4’’) 134.3 (C2’’, C6’’) 137.6 (C10) 145.5 (C10’) 148.1 (C4’) 150.6 (C2’).

***N*-(4-bromobenzyl)cinchoninium bromide (CN-(pBr)).** White solid. Yield: 72% (recrystallized from methanol/diethyl ether); m.p. 236-237 °C; $[\alpha]_D^{24} = +98° (c\ 0.1, \mathrm{MeOH})$. IR ($cm^{-1}$): 3438 (O-H), 3142 (C-$H_{Ar}$), 1508 (C=N), 1070 (C-N); Anal. Calc. for [$C_{26}H_{28}Br_2N_2O$]: C, 57.37; H, 5.19; N, 5.15 found C, 57.36; H, 5.18; N, 5.16; ^{1}H NMR (400 MHz, $CDCl_3$) δ ppm 0.81 - 0.93 (1 H, m, H7b) 1.26 - 1.43 (2 H, m, H5) 1.80 (1 H, m, H4) 2.04 - 2.15 (1 H, m, H7a) 2.24 - 2.39 (1 H, m, H3) 2.65 - 2.78 (1 H, m, H2b) 3.20 (1 H, t, *J*=11.3 Hz, H6b) 4.05 - 4.23 (2 H, m, H8, H2a) 4.46 (1 H, ddd, *J*=12.2, 9.3, 2.3 Hz, H6a) 5.19 (1 H, d, *J*=17.2 Hz, H11a) 5.23 - 5.40 (2 H, m, CH_2) 5.84 (1 H, ddd, *J*=17.3, 10.2, 7.2 Hz, H10) 6.30 (1 H, d, *J*=12.1 Hz, H9) 6.56 (1 H, d, *J*=5.9 Hz, OH) 6.89 - 7.02 (2 H, m, H3’, H6’) 7.34 (2 H, d, *J*=8.6 Hz, H3’’, H5’’) 7.47 - 7.58 (3 H, m, H2’’, H6’’, H7’) 7.86 (1 H, d, *J*=4.658 Hz, H5’) 8.24 (1 H, d, *J*=7.8 Hz, H8’) 8.84 (1 H, d, *J*=4.3 Hz, H2’); ^{13}C NMR (101 MHz, $CDCl_3$) δ ppm 21.8 (C7) 23.7 (C5) 27.1 (C4) 38.0 (C3) 53.7 (C6) 56.2 (C2) 60.4 (CH_2) 65.6 (C9) 66.9 (C8) 118.3 (C11) 119.6 (C3’) 123.0 (C5’) 123.3 (C9’) 125.4 (C4’’) 125.9 (C1’’) 127.1 (C6’) 128.2 (C7’) 129.5 (C8’) 132.0 (C2’’, C6’’) 135.0 (C10) 135.5 (C3’’, C5’’) 144.0 (C10’) 146.9 (C4’) 149.4 (C2’).

***N*-(4-nitrobenzyl)cinchoninium bromide (CN-(pNO_2)).** Yellow solid. Yield: 65% (recrystallized from methanol/diethyl ether). m.p. 239.8-240.6 °C; $[\alpha]_D^{24} = +108° (c\ 0.1, \mathrm{MeOH})$; IR($cm^{-1}$): 3427 (O-H), 3200 (C-$H_{Ar}$), 1509 (C=N), 1527 (N-O), 1351 (N-O), 1103 (C-N); Anal. Calc. for [$C_{26}H_{28}BrN_3O_3$]: C, 61.18; H, 5.53; N, 8.23 found C, 61.20; H, 5.54; N, 8.23; ^{1}H NMR (400 MHz, $CDCl_3$) δ ppm 0.81 - 0.88 (1 H, m, H7b) 1.25 - 1.41 (1 H, m, H5b) 1.77 - 1.88 (2 H, m, H4, H5a) 2.09 - 2.17 (1 H, m, H7a) 2.30 - 2.39 (1 H, m, H3) 2.70 - 2.81 (1 H, m, H2b) 3.17 (1 H, t, *J*=11.3 Hz, H6b) 4.13 - 4.26 (2 H, m, H8, H2a) 4.55 (1 H, ddd, *J*=12.1, 9.4, 2.0 Hz, H6a) 5.22 (1 H, d, *J*=17.2 Hz, H11a) 5.28 (1 H, d, *J*=10.2 Hz, H11b) 5.60 - 5.72 (1 H, m, CH2a) 5.85 (1 H, ddd, *J*=17.3, 10.4, 7.0 Hz, H10) 6.40 - 6.44 (1 H, m, H9) 6.47 - 6.53 (2 H, m, OH, CH2b) 6.93 - 7.02 (2 H, m, H3’, H7’) 7.51 - 7.55 (1 H, m, H6’) 7.84 (1 H, d, *J*=4.3 Hz, H5’) 7.87 - 7.95 (4 H, m, Ar H’’) 8.22 - 8.28 (1 H, m, H8’) 8.84 (1 H, d, *J*=4.7 Hz, H2’); ^{13}C NMR (101 MHz, $CDCl_3$) δ ppm 21.8 (C7) 23.7 (C5) 27.0 (C4) 37.9 (C3) 54.3 (C6) 56.6 (C2) 59.7 (CH_2) 65.6 (C9) 67.4 (C8) 118.6 (C11) 119.5 (C3’) 122.8 (C5’) 123.2 (C3’’, C5’’) 123.2 (C9’) 127.1 (C6’) 128.2 (C7’) 129.7 (C8’) 134.2 (C1’’) 134.6 (C10) 135.4 (C2’’, C6’’).

***N*-(4-chlorobenzyl)cinchoninium bromide (CN-(pCl)).** White solid. Yield: 72% (recrystallized from methanol/diethyl ether). m.p. 248.6-249.8 °C; $[\alpha]_D^{24} = +173° (c\ 0.1, \mathrm{MeOH})$; IR($cm^{-1}$): 3435 (O-H), 3131 (C-$H_{Ar}$), 1506 (C=N), 1062 (C-N); Anal. Calc. for [$C_{26}H_{28}BrClN_2O$]: C, 62.47; H, 5.65; N, 5.60 found C, 62.43; H, 5.67; N, 5.61; ^{1}H NMR (400 MHz, $CDCl_3$) δ ppm 0.81 - 0.93 (1 H, m, H7b) 1.26 - 1.43 (2 H, m, H5) 1.80 (1 H, m, H4) 2.04 - 2.15 (1 H, m, H7a) 2.24 - 2.39 (1 H, m, H3) 2.65 - 2.78 (1

H, m, H2b) 3.20 (1 H, t, *J*=11.3 Hz, H6b) 4.05 - 4.23 (2 H, m, H8, H2a) 4.46 (1 H, ddd, *J*=12.2, 9.3, 2.3 Hz, H6a) 5.19 (1 H, d, *J*=17.2 Hz, H11a) 5.23 - 5.40 (2 H, m, CH_2) 5.84 (1 H, ddd, *J*=17.2, 10.2, 7.2 Hz, H10) 6.30 (1 H, d, *J*=12.1 Hz, H9) 6.56 (1 H, d, *J*=5.9 Hz, OH) 6.89 - 7.02 (2 H, m, H3', H6') 7.34 (2 H, d, *J*=8.6 Hz, H3'', H5'') 7.47 - 7.58 (3 H, m, H2'', H6'', H7') 7.86 (1 H, d, *J*=4.7 Hz, H5') 8.24 (1 H, d, *J*=7.8 Hz, H8') 8.84 (1 H, d, *J*=4.3 Hz, H2'); ^{13}C NMR (101 MHz, $CDCl_3$) δ ppm 21.8 (C7) 23.7 (C5) 27.1 (C4) 38.0 (C3) 53.7 (C6) 56.2 (C2) 60.4 (CH_2) 65.6 (C9) 66.9 (C8) 118.3 (C11) 119.6 (C3') 123.0 (C5') 123.3 (C9') 125.4 (C4'') 125.9 (C1'') 127.1 (C6') 128.2 (C7') 129.5 (C8') 132.0 (C2'', C6'') 135.0 (C10) 135.5 (C3'', C5'') 144.0 (C10') 146.9 (C4') 149.4 (C2').

***N*-(3-bromobenzyl)cinchoninium bromide (CN-(mBr)).** White solid. Yield: 53% (recrystallized from acetonitrile/diethyl ether). m.p. 227.9-229.1 °C; $[\alpha]_D^{24} = +117°$ (c 0.1, MeOH); IR(cm^{-1}): 3139 (O-H), 3454 (C-H_{Ar}), 1509 (C=N), 1060 (C-N); Anal. Calc. for [$C_{26}H_{28}Br_2N_2O$]: C, 62.47; H, 5.65; N, 5.60 found C, 62.50; H, 5.63; N, 5.57; ^1H NMR (300 MHz, DMSO-d_6) δ ppm 1.00 - 1.13 (1 H, m, H7b) 1.69 - 1.82 (2 H, m, H5) 1.88 (1 H, m, H4) 2.30 (1 H, t, *J*=11.7 Hz, H7a) 2.60 - 2.74 (1 H, m, H3) 2.92 - 3.07 (1 H, m. H2b) 3.50 (1 H, t, *J*=11.3 Hz, H6b) 3.83 - 4.03 (2 H, m, H8, H2a) 4.24 (1 H, t, *J*=9.6 Hz, H6a) 4.94 (1 H, d, *J*=12.4 Hz, H11a) 5.12 (1 H, d, *J*=12.4 Hz, H11b) 5.19 - 5.29 (2 H, m, CH_2) 6.01 (1 H, ddd, *J*=17.5, 10.2, 7.0 Hz, H10) 6.49 (1 H, s, H9) 6.77 (1 H, d, *J*=3.8 Hz, OH) 7.49 - 7.60 (1 H, m, H3') 7.70 - 7.90 (5 H, m, H Ar'', H7') 8.04 - 8.16 (2 H, m, H, H5', H6') 8.34 (1 H, d, *J*=8.3 Hz, H8') 8.99 (1 H, d, *J*=4.5 Hz, H2'); ^{13}C NMR (75 MHz, DMSO-d_6) δ ppm 21.1 (C7) 23.5 (C5) 26.8 (C4) 37.1 (C3) 54.5 (C6) 56.5 (C2) 61.8 (CH_2) 65.2 (C8) 67.9 (C9) 117.5 (C11) 120.5 (C3') 122.5 (C9') 124.2 (C5') 124.8 (C3'') 127.7 (C6') 129.9 (C7') 130.3 (C8') 131.0 (C1'') 131.5 (C6'') 133.4 (C5'') 133.5 (C4'') 136.6 (C2'') 137.6 (C10) 145.4 (C10') 148.1 (C4') 150.6 (C2').

***N*-(3-chlorobenzyl)cinchoninium chloride (CN-(mCl)).** White solid. Yield: 30% (recrystallized from acetonitrile/diethyl ether). m.p. 240.5-241.5°C; $[\alpha]_D^{24} = +223°$ (c 0.1, MeOH); IR(cm^{-1}): 3417 (O-H), 3085 (C-H_{Ar}), 1511 (C=N), 1100 (C-N); Anal. Calc. for [$C_{26}H_{28}Cl_2N_2O$]: C, 68.57; H, 6.20; N, 6.15 found C, 68.55; H, 6.21; N, 6.17, ^1H NMR (300 MHz, DMSO-d_6) δ ppm 1.00 - 1.13 (1 H, m, H7b) 1.69 - 1.82 (2 H, m, H5) 1.88 (1 H, m, H4) 2.30 (1 H, t, *J*=11.7 Hz, H7a) 2.60 - 2.74 (1 H, m, H3) 2.92 - 3.07 (1 H, m. H2b) 3.50 (1 H, t, *J*=11.3 Hz, H6b) 3.83 - 4.03 (2 H, m, H8, H2a) 4.24 (1 H, t, *J*=9.6 Hz, H6a) 4.94 (1 H, d, *J*=12.4 Hz, H11a) 5.12 (1 H, d, *J*=12.4Hz, H11b) 5.19 - 5.29 (2 H, m, CH_2) 6.01 (1 H, ddd, *J*=17.5, 10.2, 7.0 Hz, H10) 6.49 (1 H, s, H9) 6.77 (1 H, d, *J*=3.8 Hz, OH) 7.49 - 7.60 (1 H, m, H3') 7.70 - 7.90 (5 H, m, H Ar'', H7') 8.04 - 8.16 (2 H, m, H, H5', H6') 8.34 (1 H, d, *J*=8.3 Hz, H8') 8.99 (1 H, d, *J*=4.5 Hz, H2'); ^{13}C NMR (75 MHz, DMSO-d_6) δ ppm 21.1 (C7) 23.5 (C5) 26.8 (C4) 37.1 (C3) 54.5 (C6) 56.5 (C2) 61.8 (CH_2) 65.2 (C8) 67.9 (C9) 117.5 (C11) 120.5 (C3') 122.5 (C9') 124.2 (C5') 124.8 (C3'') 127.7 (C6') 129.9 (C7') 130.3 (C8') 131.0 (C1'') 131.5 (C6'') 133.4 (C5'') 133.5 (C4'') 136.6 (C2'') 137.6 (C10) 145.4 (C10') 148.1 (C4') 150.6 (C2').

***N*-(3-nitrobenzyl)cinchoninium bromide (CN-(mNO$_2$)).** White solid. Yield: 67% (recrystallized from acetonitrile/diethyl ether). m.p. 248.2-249.6 °C; $[\alpha]_D^{24} = +176° (c\ 0.1, \mathrm{MeOH})$; IR(cm^{-1}): 3417 (O-H), 3176 (C-H$_{Ar}$), 1527 (N-O), 1509 (C=N), 1346 (N-O), 1062 (C-N); Anal. Calc. for [$C_{26}H_{28}BrN_3O_3$]: C, 61.18; H, 5.53; N, 8.23 found C, 61.19; H, 5.51; N, 8.25; ^{1}H NMR (600 MHz, DMSO-d_6) δ ppm 1.00 - 1.14 (1 H, m, H7b) 1.71 - 1.85 (2 H, m, H5) 1.90 (1 H, m, H4) 2.28 - 2.39 (1 H, m, H7a) 2.61 (1 H, d, *J*=8.1 Hz, H3) 2.99 (1 H, d, *J*=9.5 Hz, H2b) 3.53 (1 H, t, *J*=11.4 Hz, H6b) 3.88 - 4.06 (2 H, m, H2a, H8) 4.28 (1 H, t, *J*=9.2 Hz, H6a) 5.10 (1 H, d, *J*=12.5 Hz, H11b) 5.20 - 5.35 (3 H, m, H11a, CH$_2$) 6.01 (1 H, ddd, *J*=17.1, 10.5, 7.0 Hz, H10) 6.53 (1 H, s, H9) 6.80 (1 H, d, *J*=3.7 Hz, OH) 7.78 (1 H, t, *J*=7.3 Hz, H3') 7.81 - 7.92 (3 H, m, H6', H5'', H6'') 8.13 (1 H, d, *J*=8.1 Hz, H7') 8.24 (1 H, d, *J*=7.3 Hz, H5') 8.36 (1 H, d, *J*=8.0 Hz, H8') 8.43 (1 H, dd, *J*=8.0, 1.5 Hz, H4'') 8.70 (1 H, m, H2'') 9.00 (1 H, d, *J*=4.4 Hz, H2'); ^{13}C NMR (151 MHz, DMSO-d_6) δ ppm 20.6 (C7) 23.0 (C5) 26.3 (C4) 36.7 (C3) 39.1 (C6) 54.1 (C6) 56.0 (C2) 61.0 (CH$_2$) 64.7 (C9) 67.6 (C8) 117.0 (C11) 120.0 (C3') 123.7 (C5') 124.3 (C9') 124.9 (C6') 127.2 (C7') 128.4 (C5'') 129.4 (C6'') 129.8 (C8') 129.9 (C1'') 130.5 (C4'') 137.0 (C2'') 140.2 (C10) 144.8 (C10') 147.6 (C3'') 148.0 (C4') 150.2 (C2').

.

S2. ^{1}H and ^{13}C NMR spectra of prepared compounds

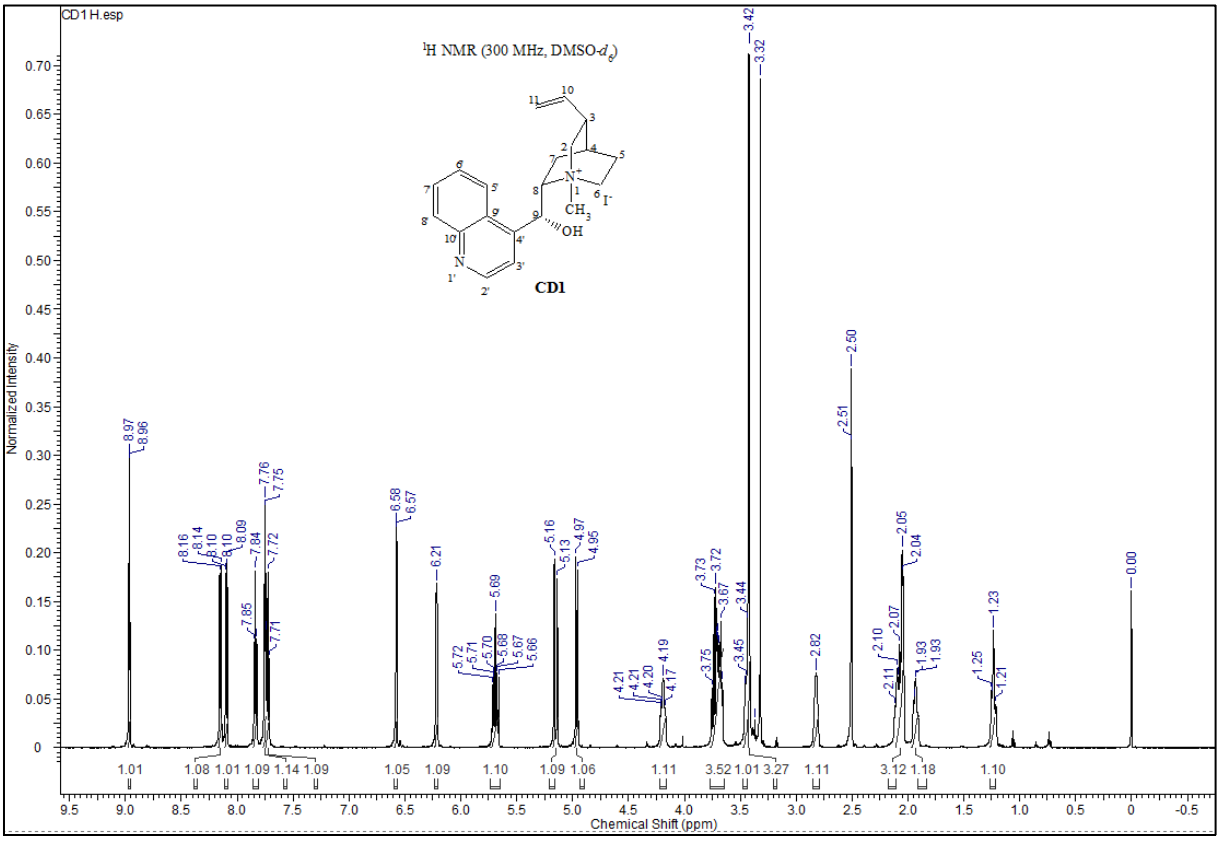

Figure 1. ^{1}H NMR spectra of **CD-Met**

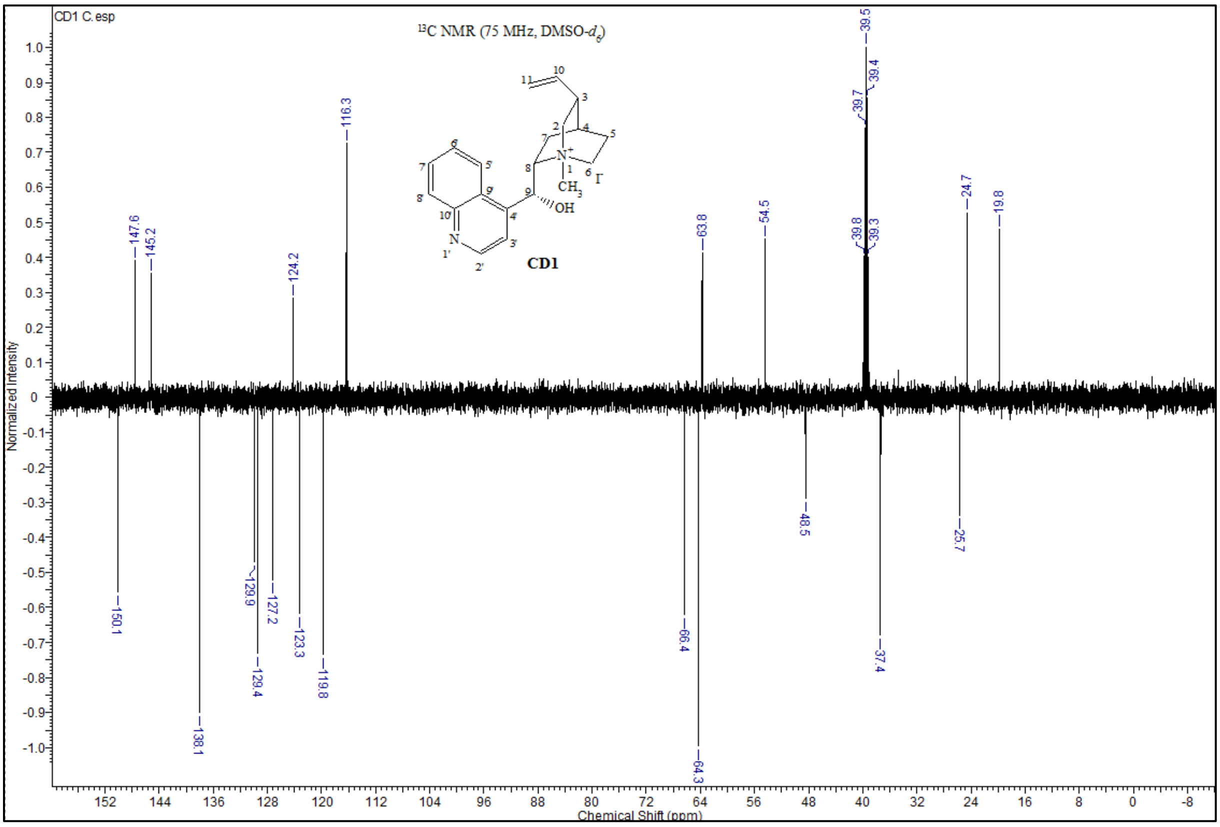

Figure 2. ^{13}C NMR spectra of **CD-Met**

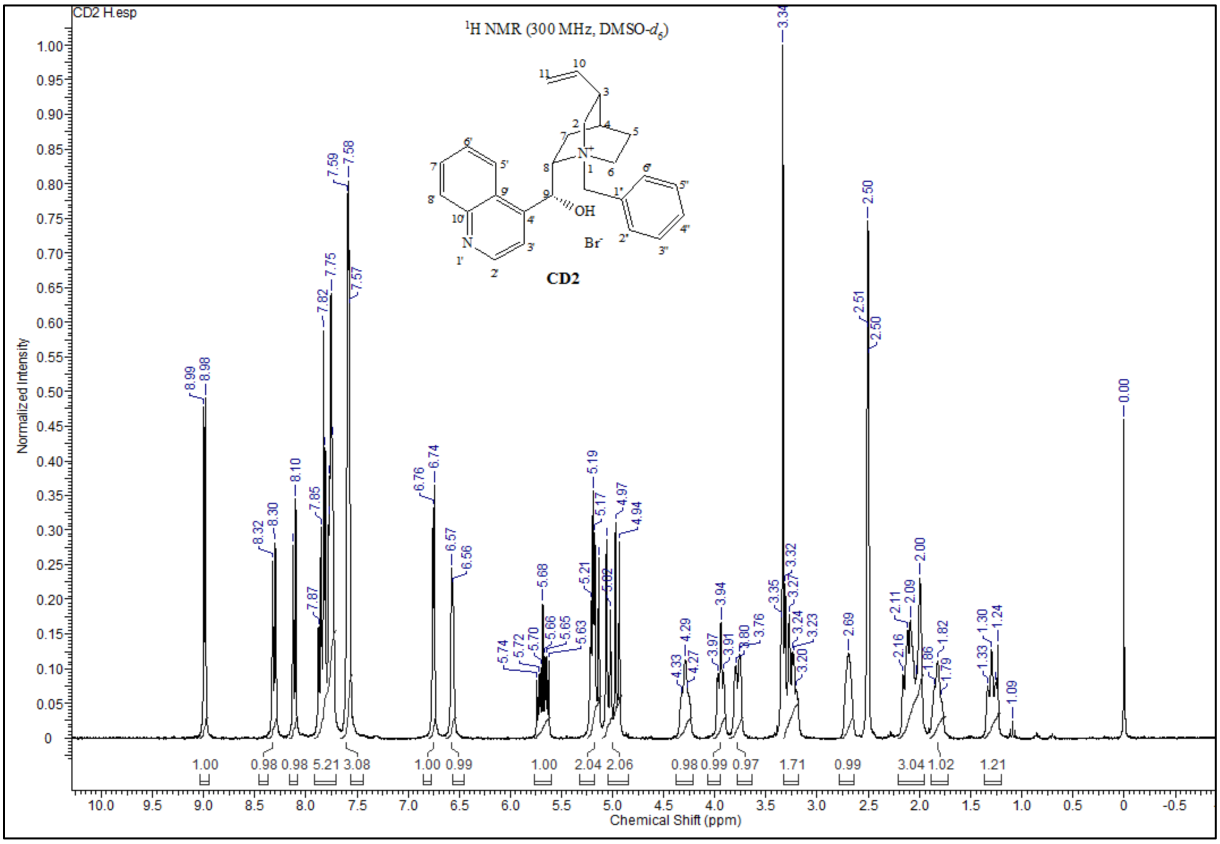

Figure 3. ^{1}H NMR spectra of **CD-Bzl**

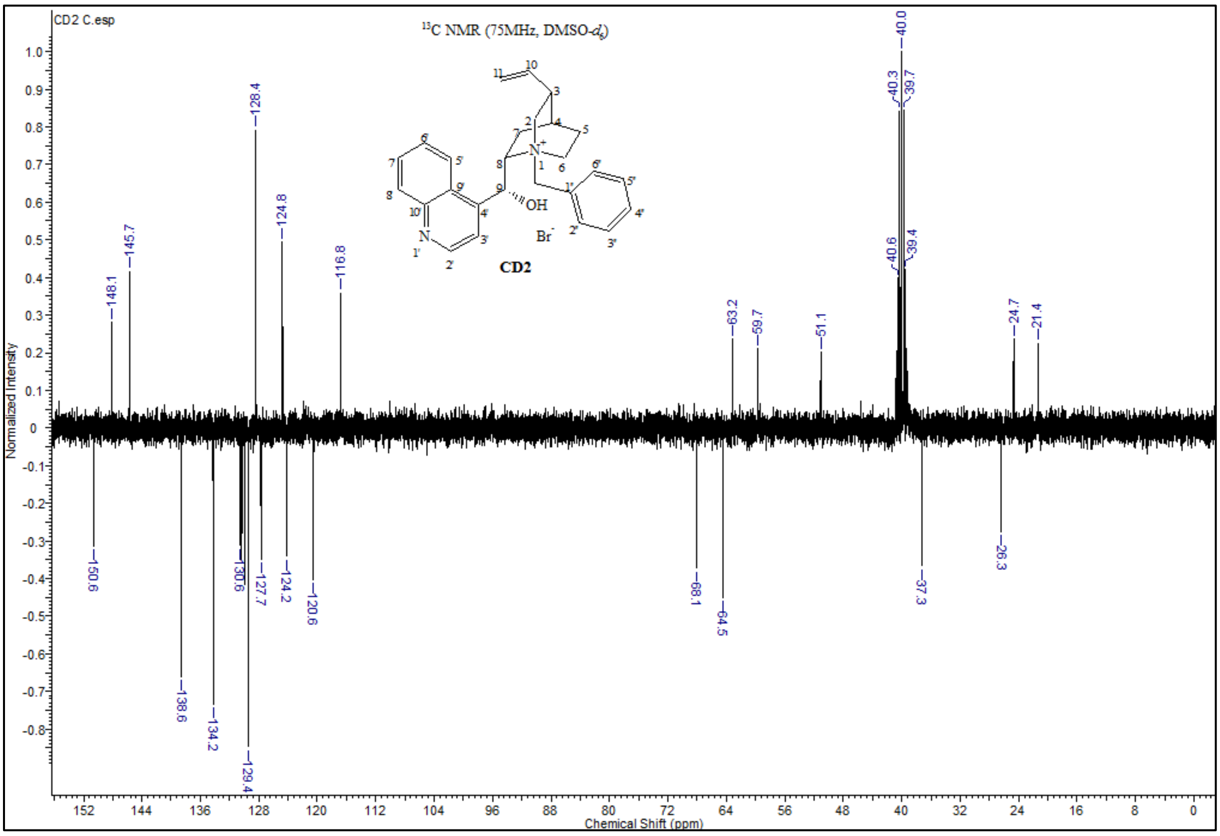

Figure 4. ^{13}C NMR spectra of **CD-Bzl**

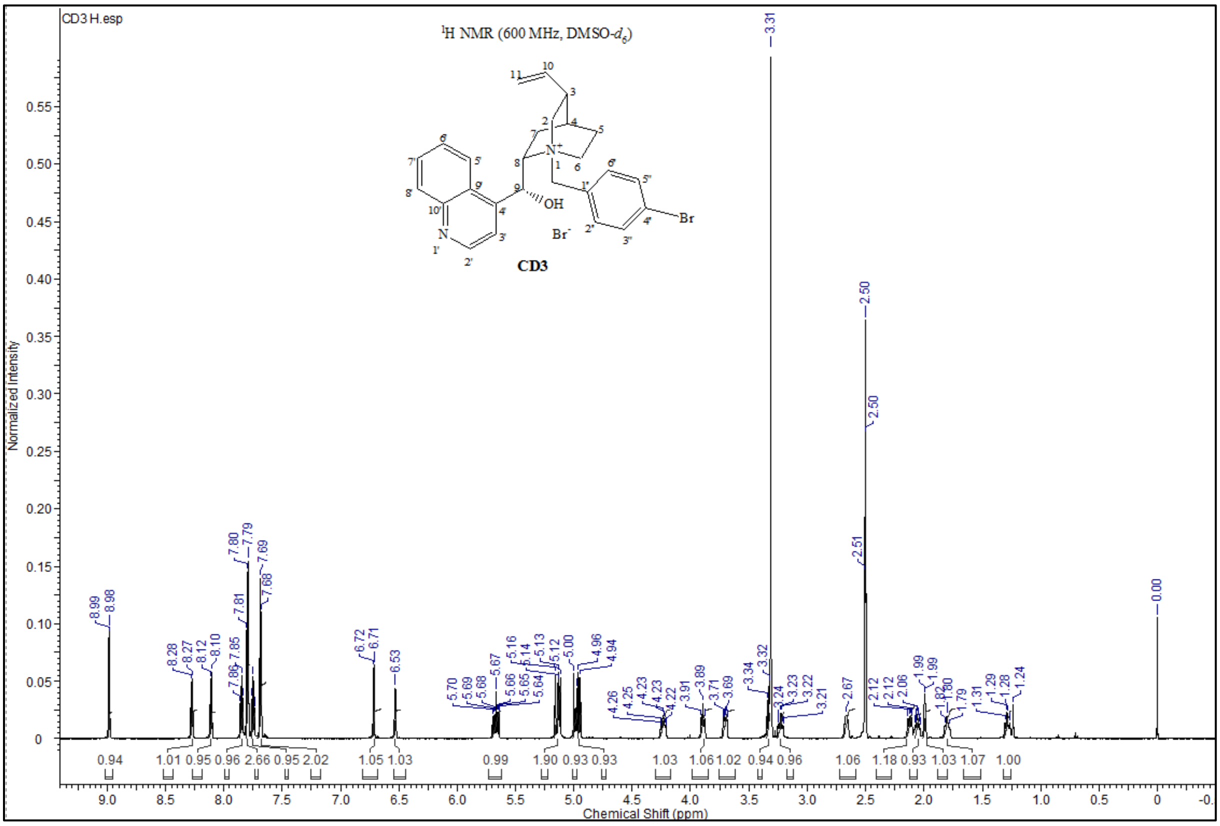

Figure 5. ^{1}H NMR spectra of **CD-(pBr)**

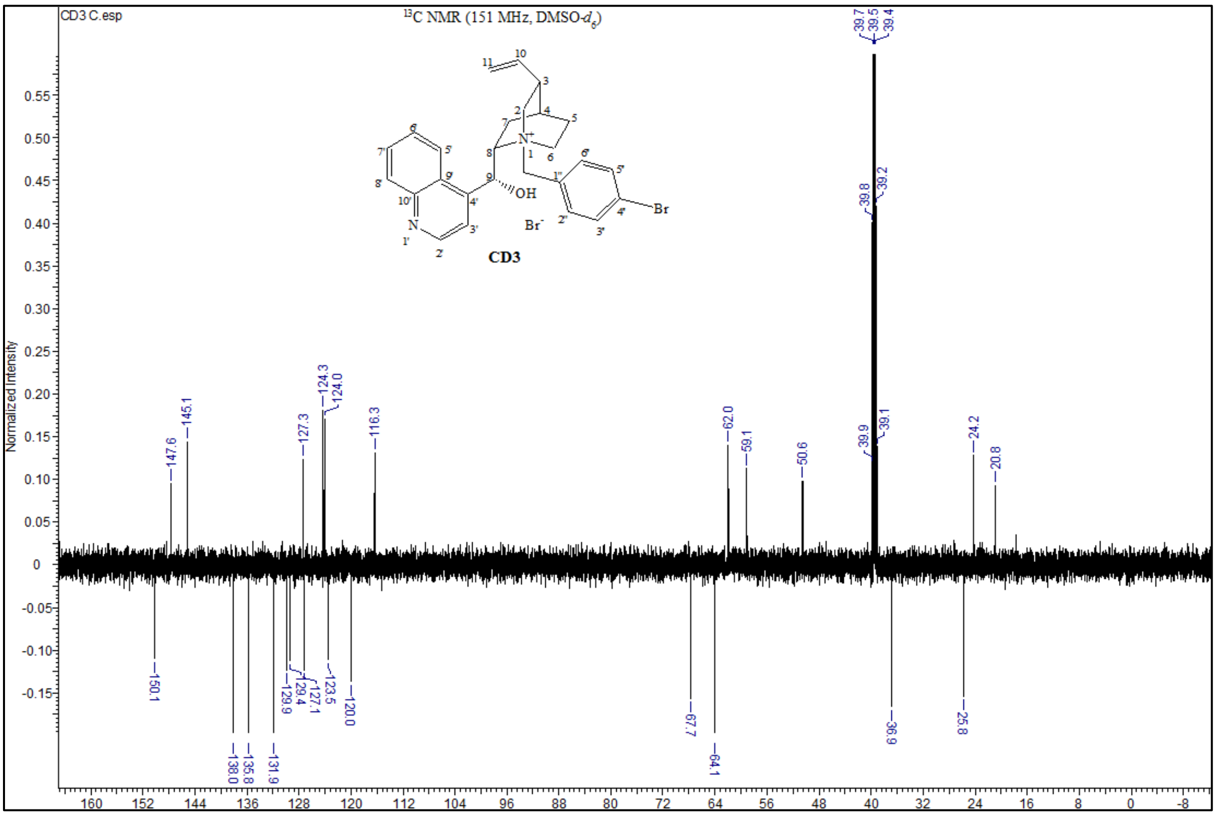

Figure 6. ^{13}C NMR spectra of **CD-(pBr)**

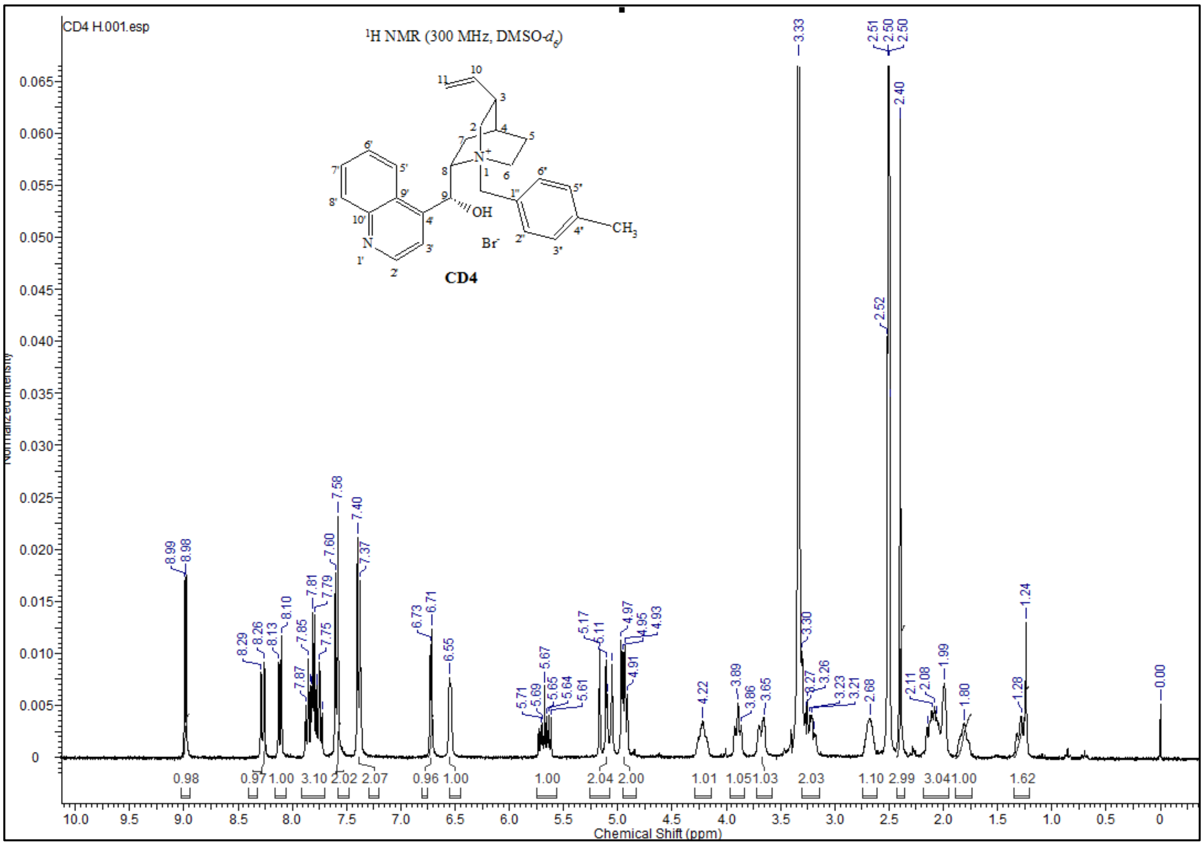

Figure 7. ^{1}H NMR spectra of **CD-(pMet)**

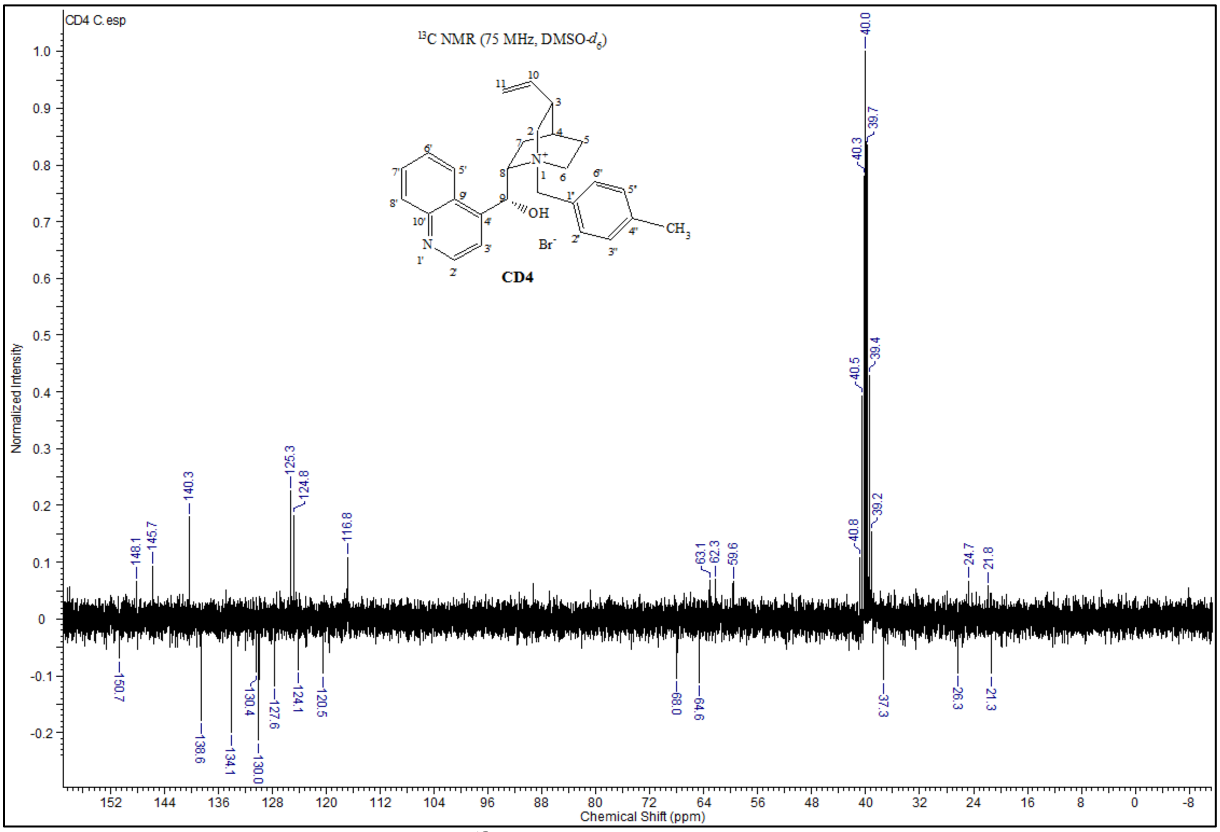

Figure 8. ^{13}C NMR spectra of **CD-(pMet)**

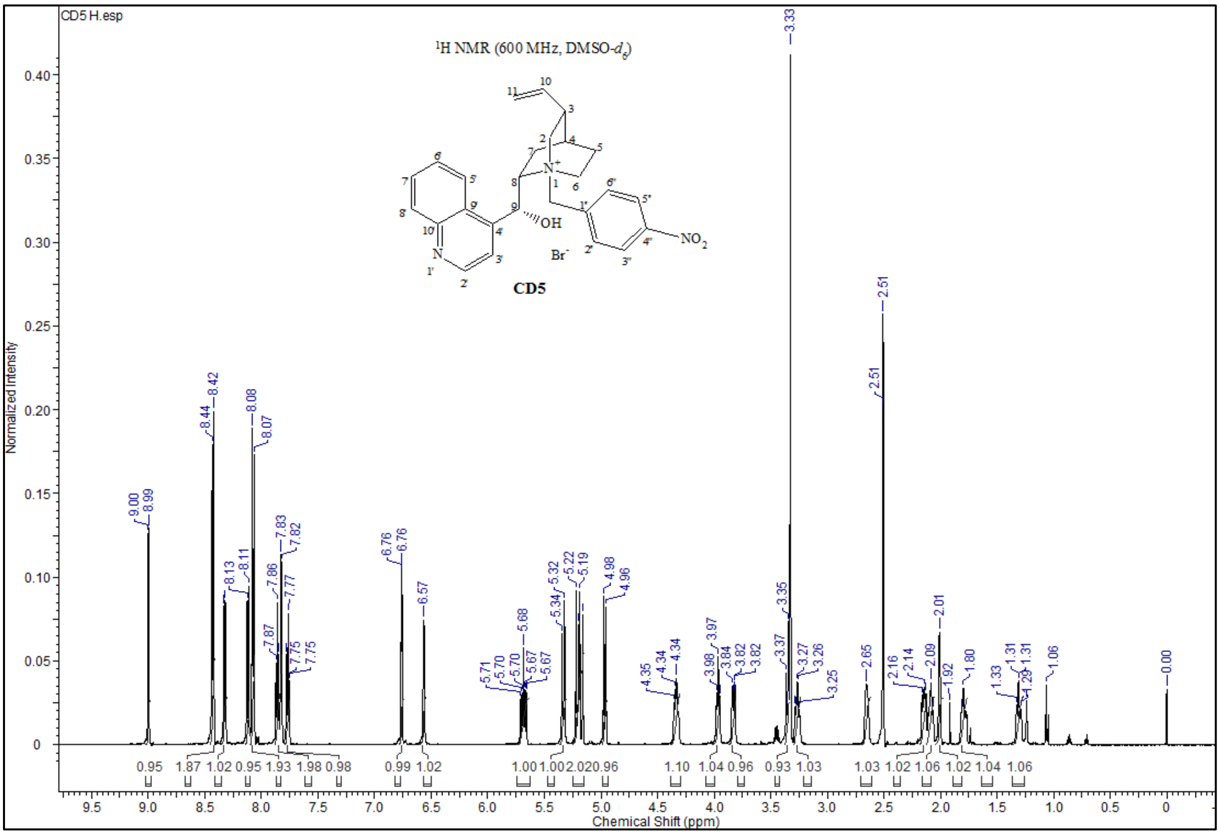

Figure 9. ^{1}H NMR spectra of **CD-(pNO$_2$)**

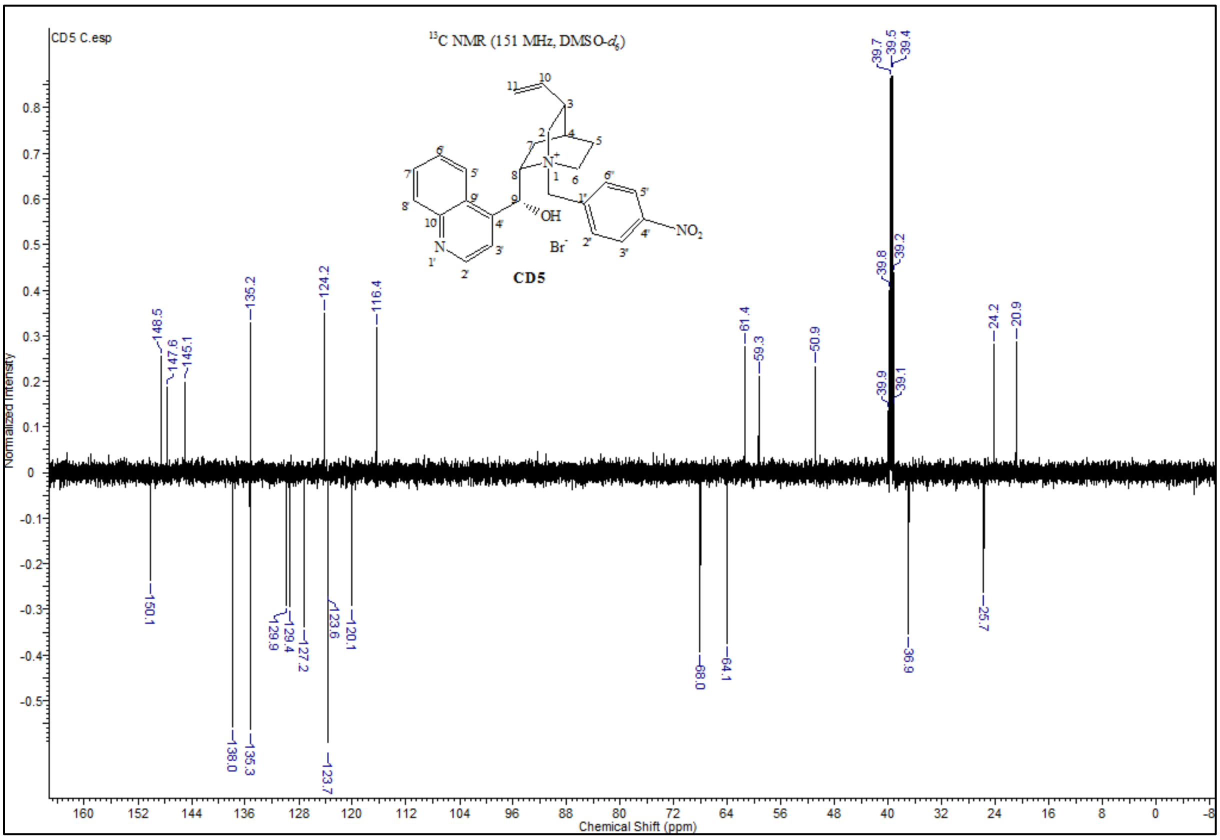

Figure 10. ^{13}C NMR spectra of **CD-(pNO$_2$)**

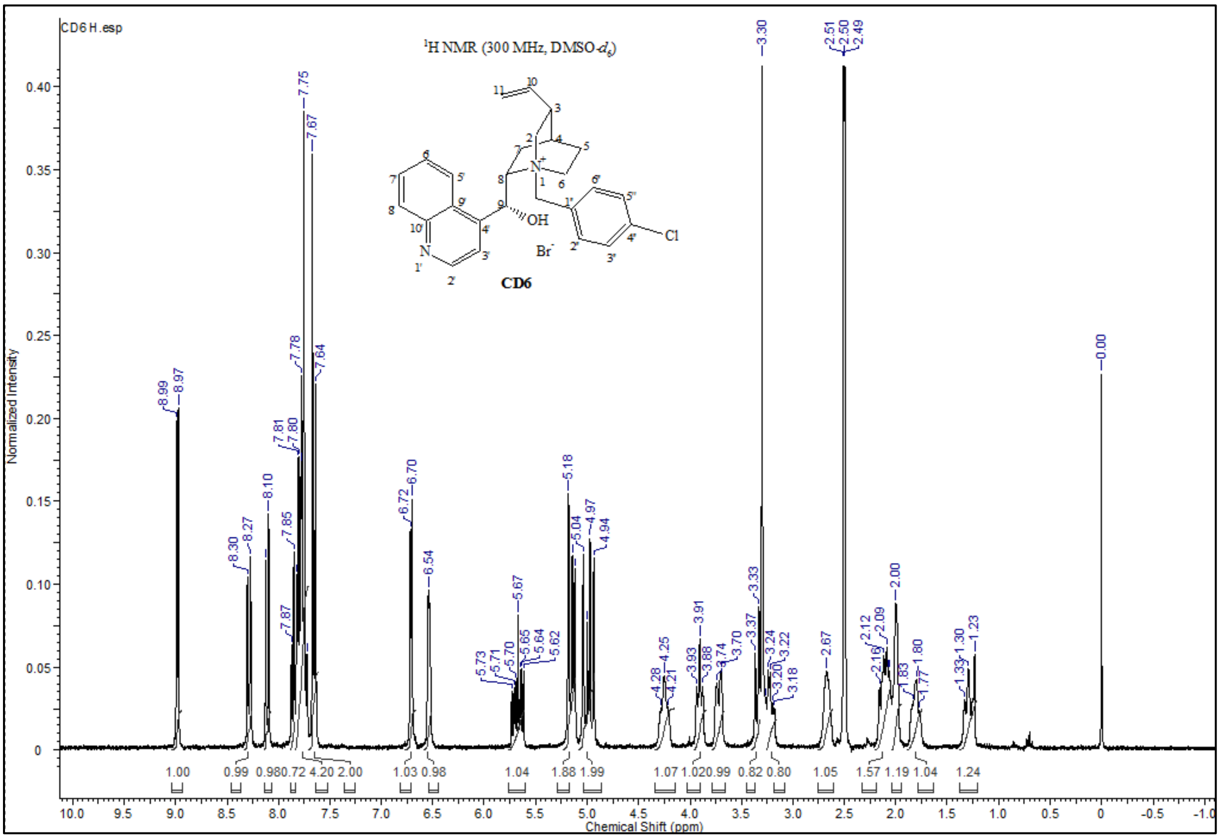

Figure 11. ^{1}H NMR spectra of **CD-(pCl)**

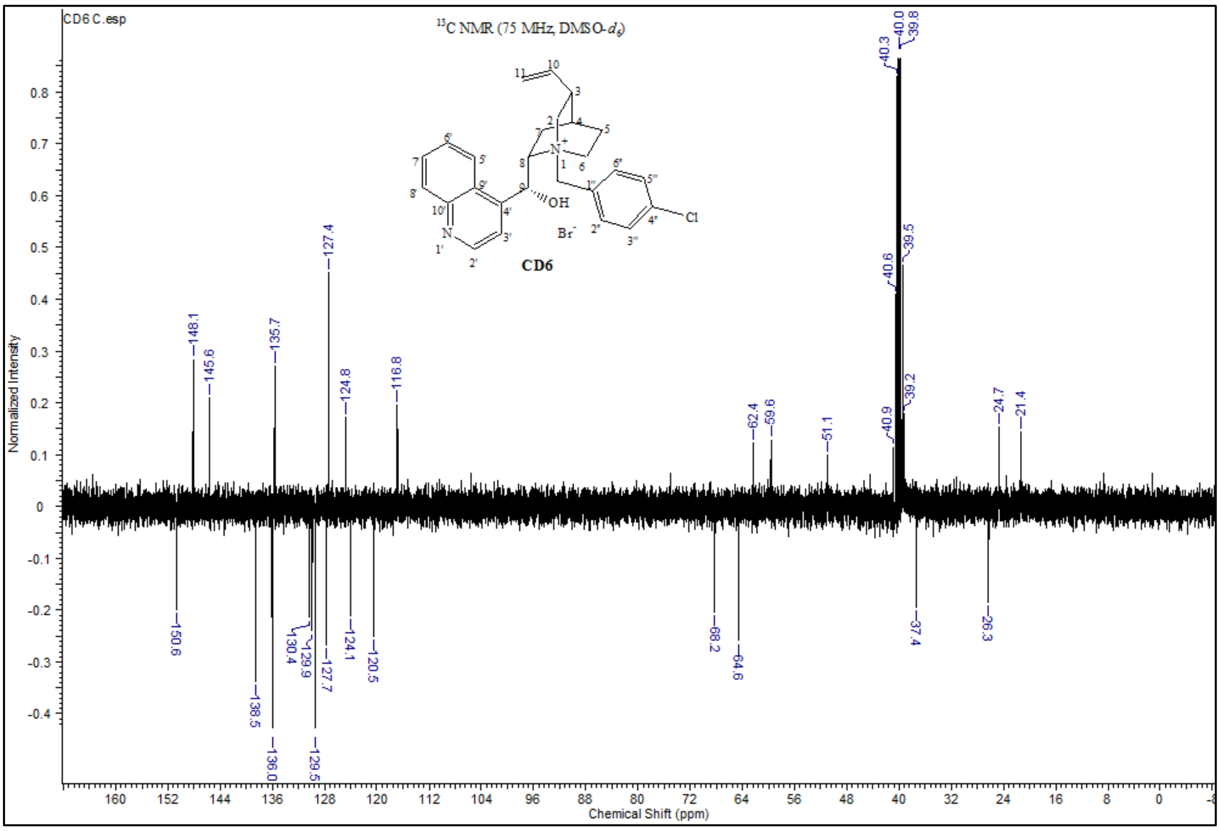

Figure 12. ^{13}C NMR spectra of **CD-(pCl)**

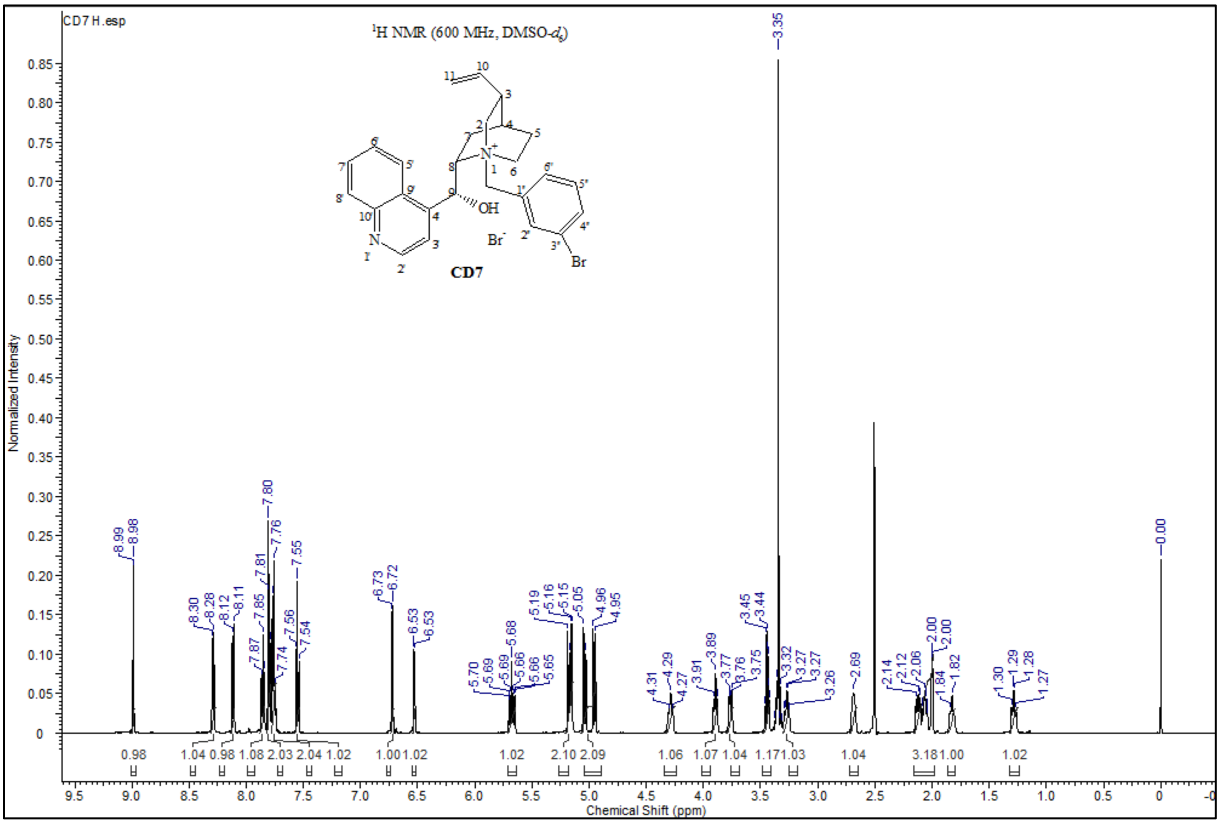

Figure 13. ^{1}H NMR spectra of **CD-(mBr)**

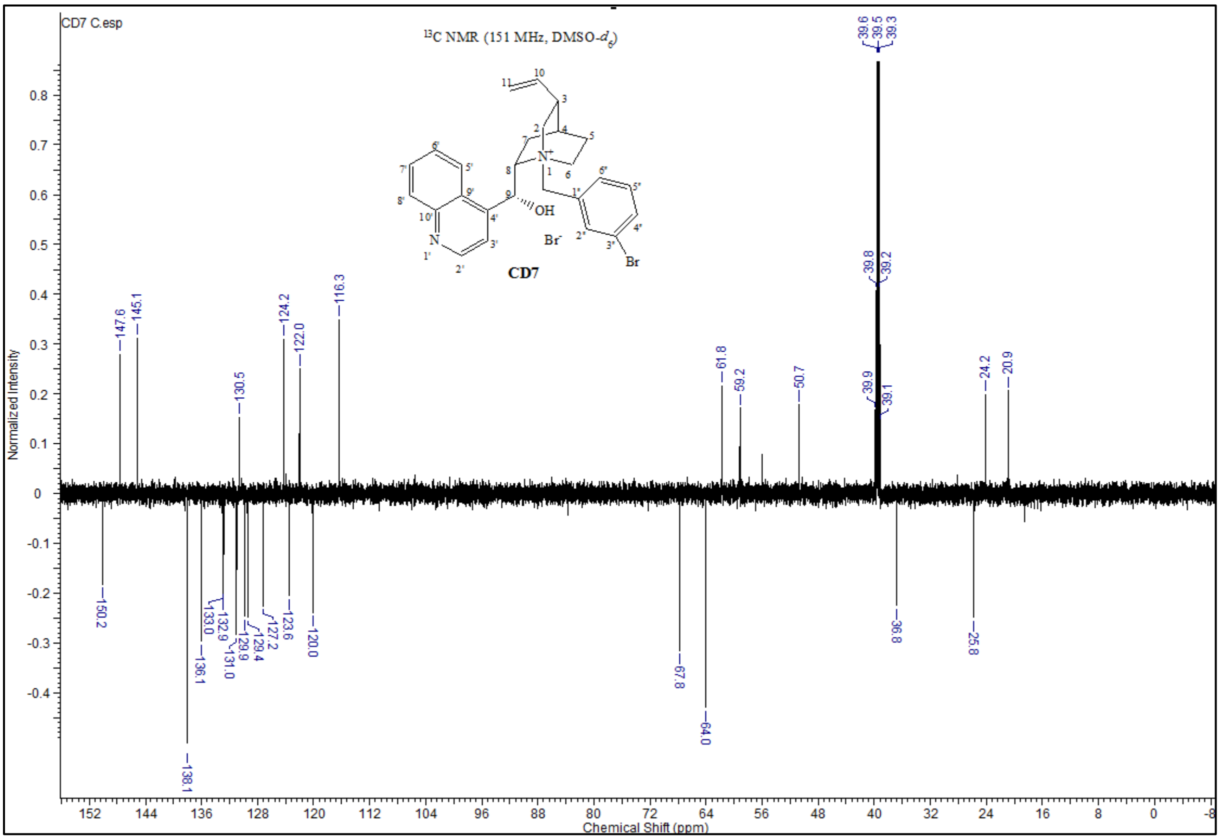

Figure 14. ^{13}C NMR spectra of **CD-(mBr)**

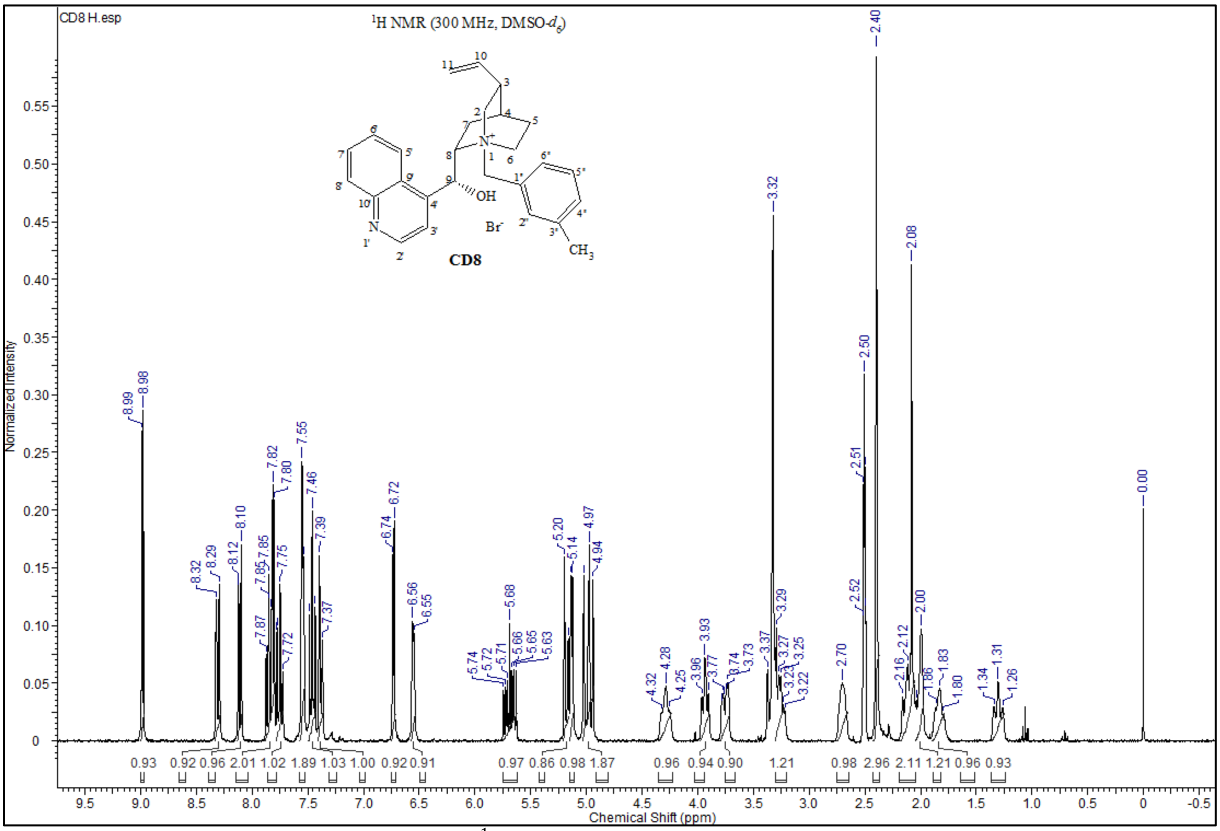

Figure 15. ^{1}H NMR spectra of **CD-(mMet)**

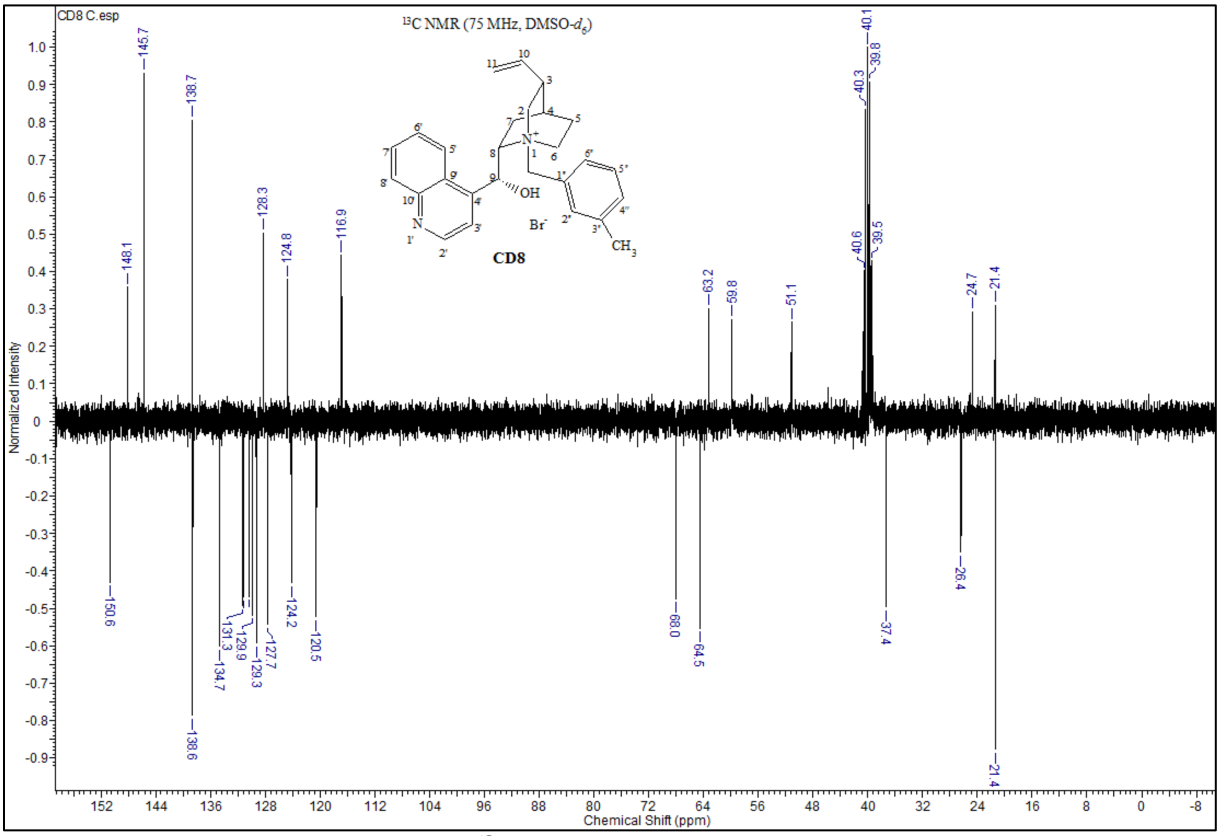

Figure 16. ^{13}C NMR spectra of **CD-(mMet)**

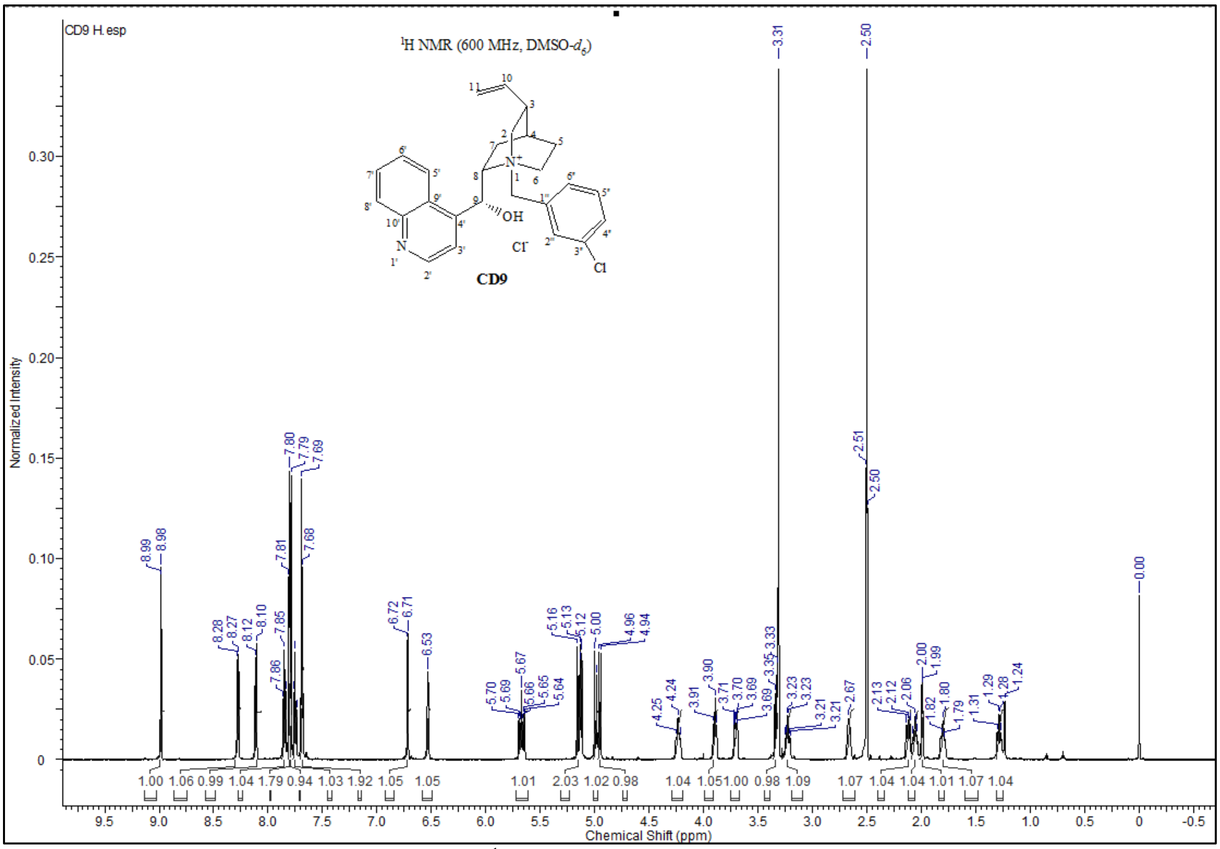

Figure 17. ^{1}H NMR spectra of **CD-(mCl)**

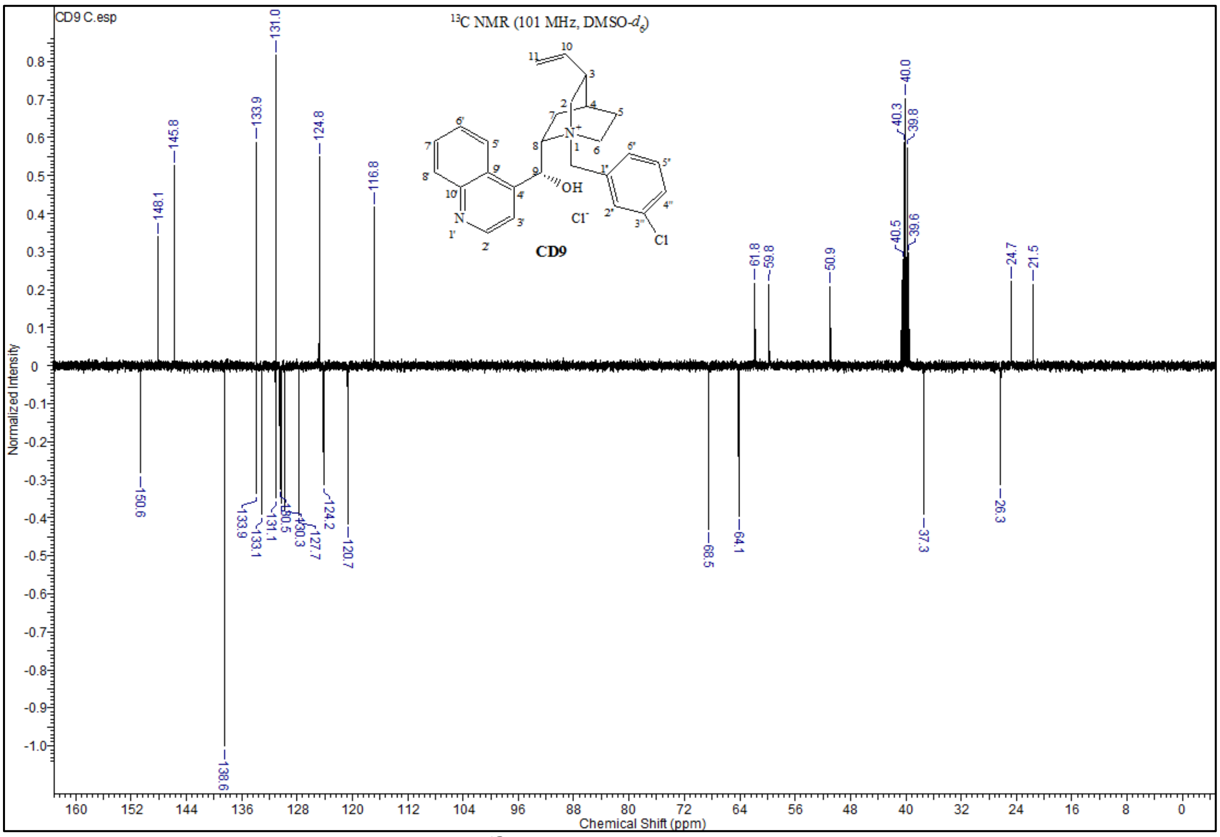

Figure 18. ^{13}C NMR spectra of **CD-(mCl)**

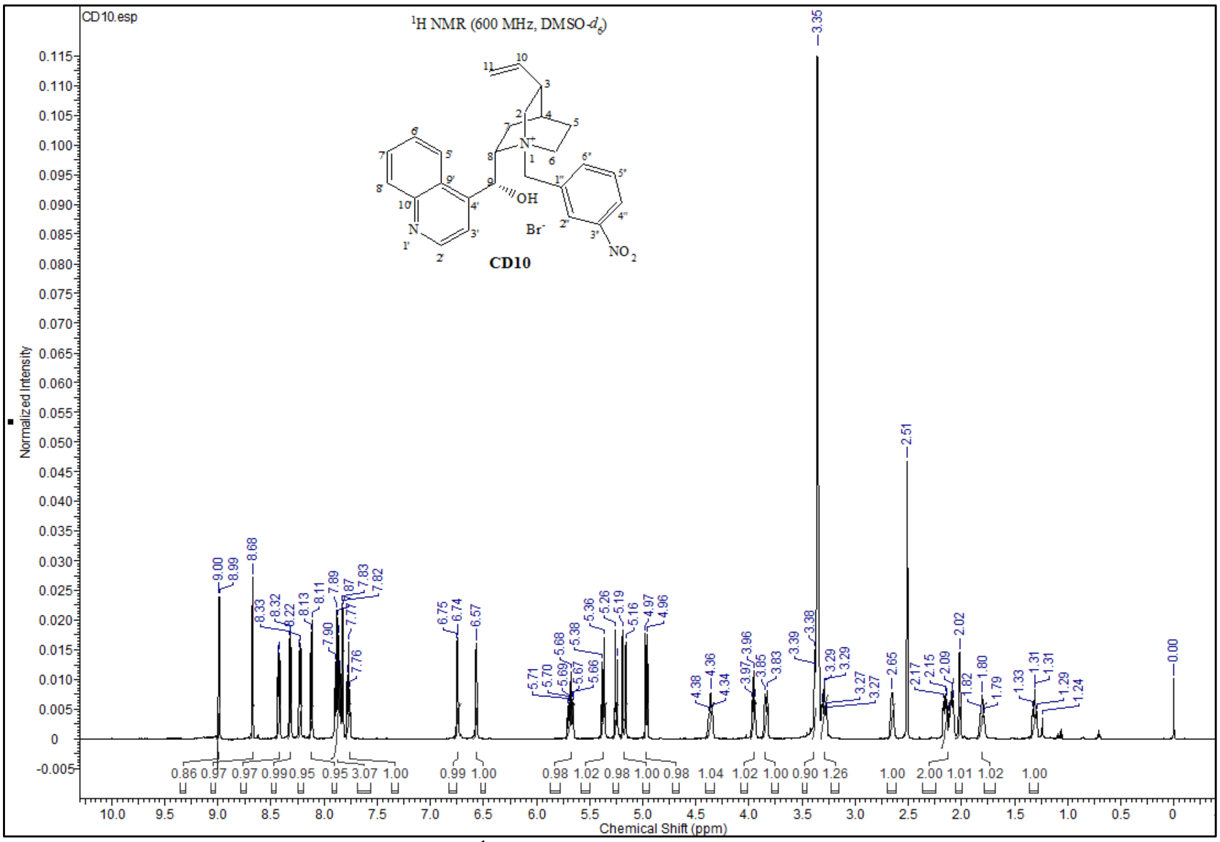

Figure 19. ^{1}H NMR spectra of **CD-(mNO$_2$)**

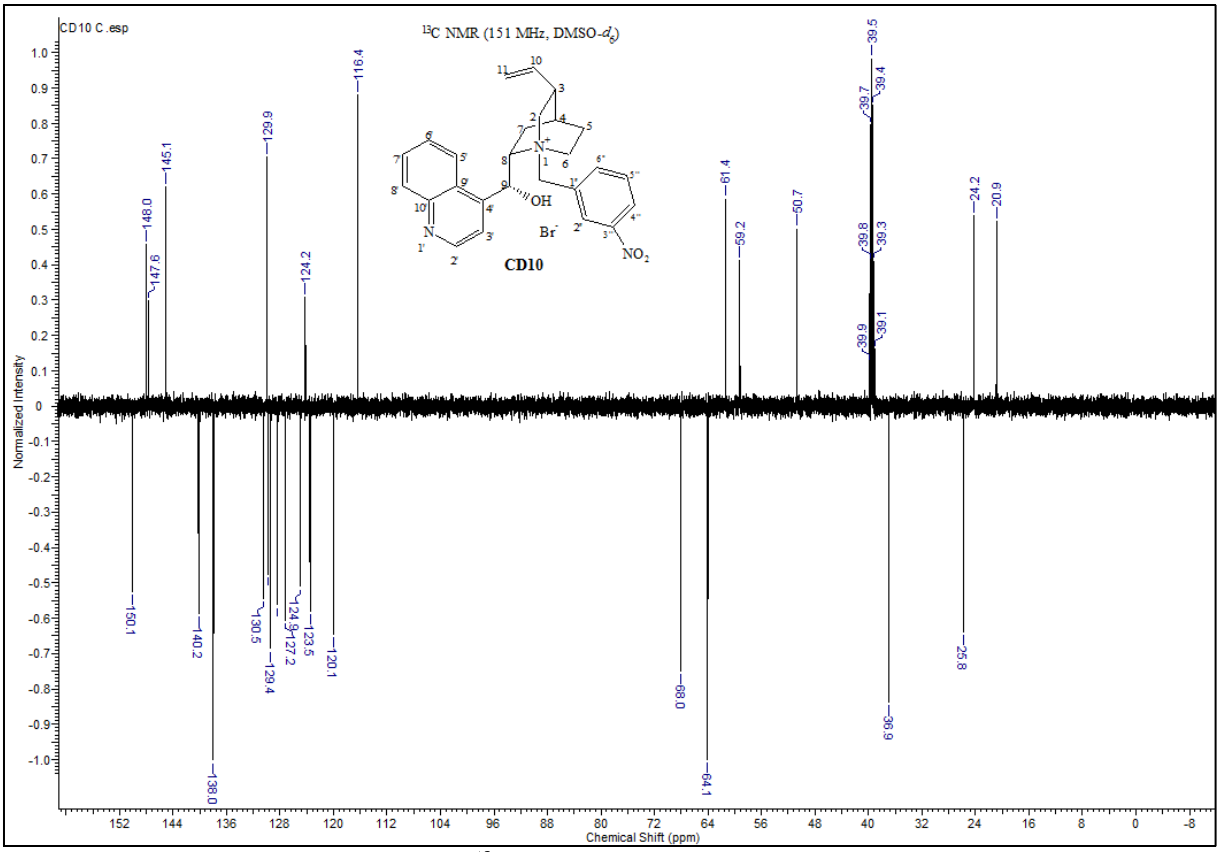

Figure 20. ^{13}C NMR spectra of **CD-(mNO$_2$)**

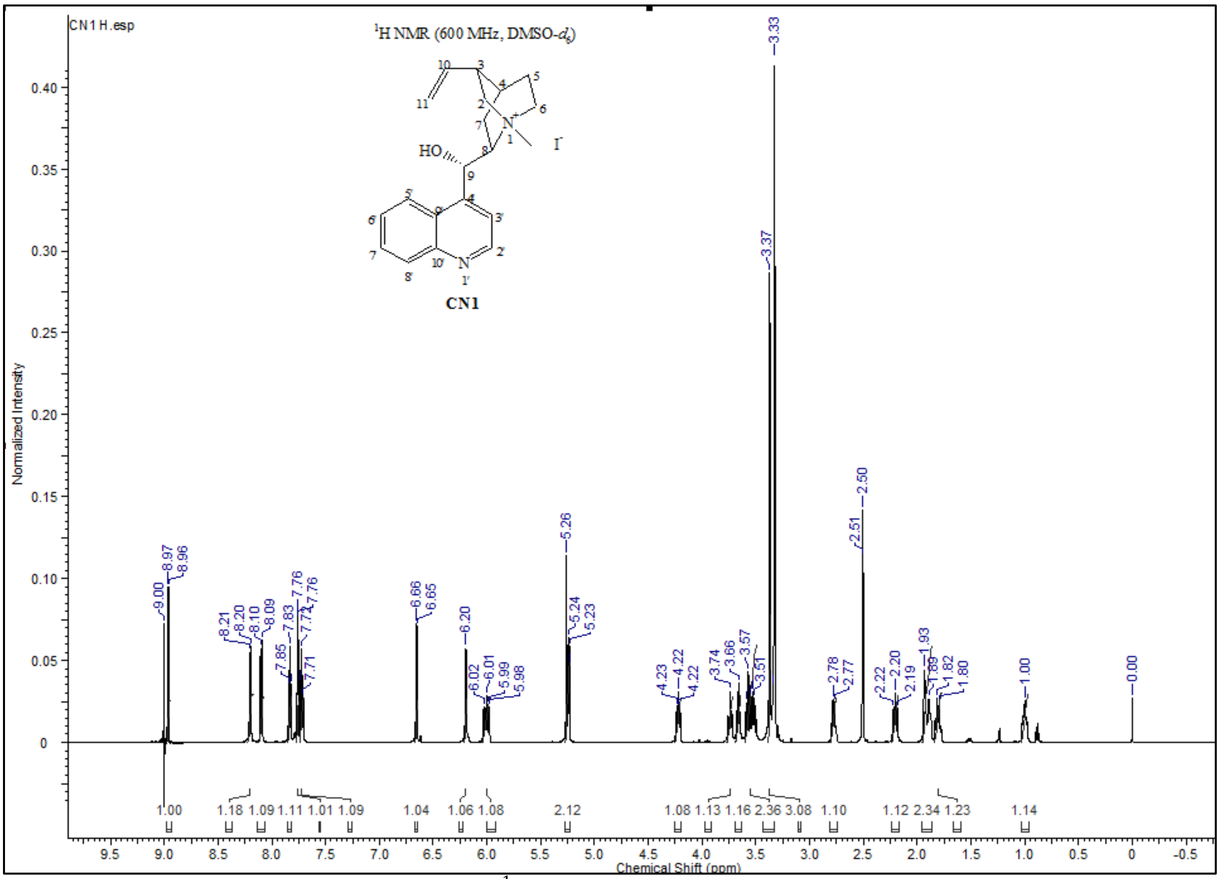

Figure 21. ^{1}H NMR spectra of **CN-Met**

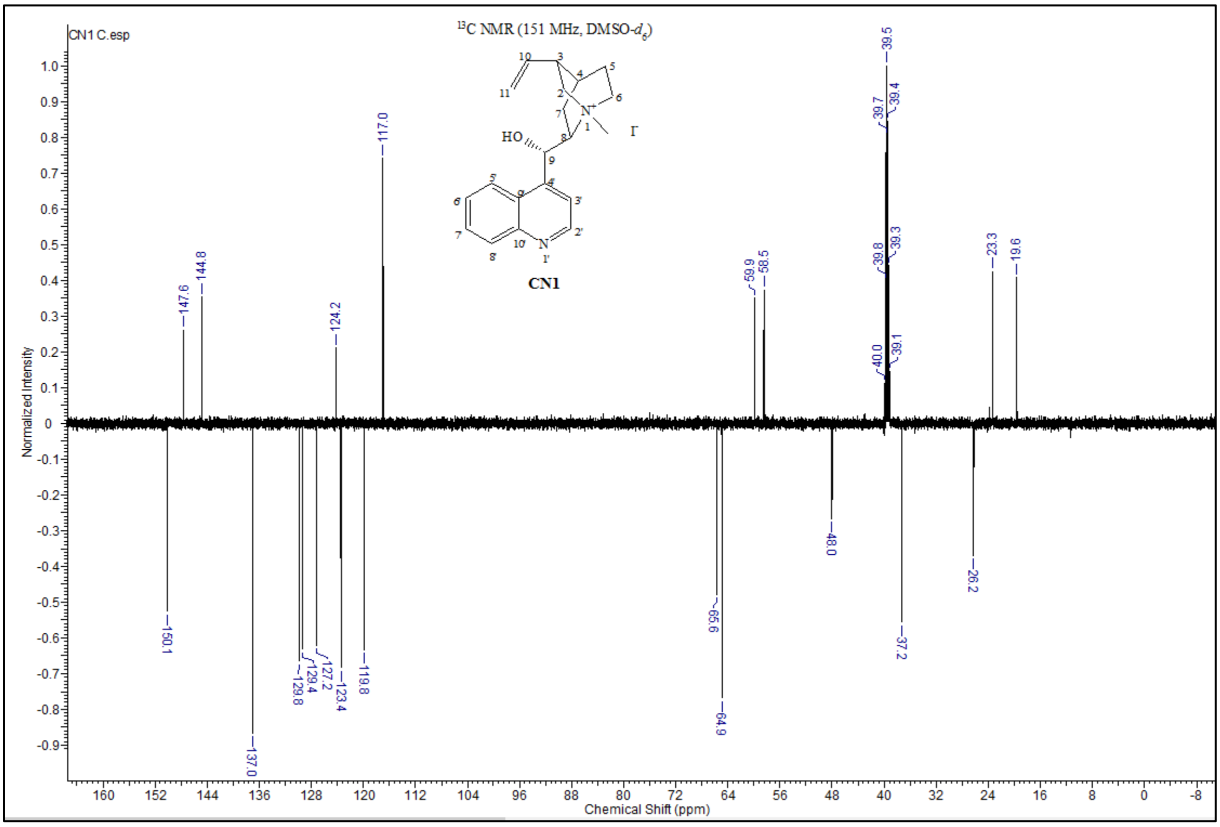

Figure 22. ^{13}C NMR spectra of **CN-Met**

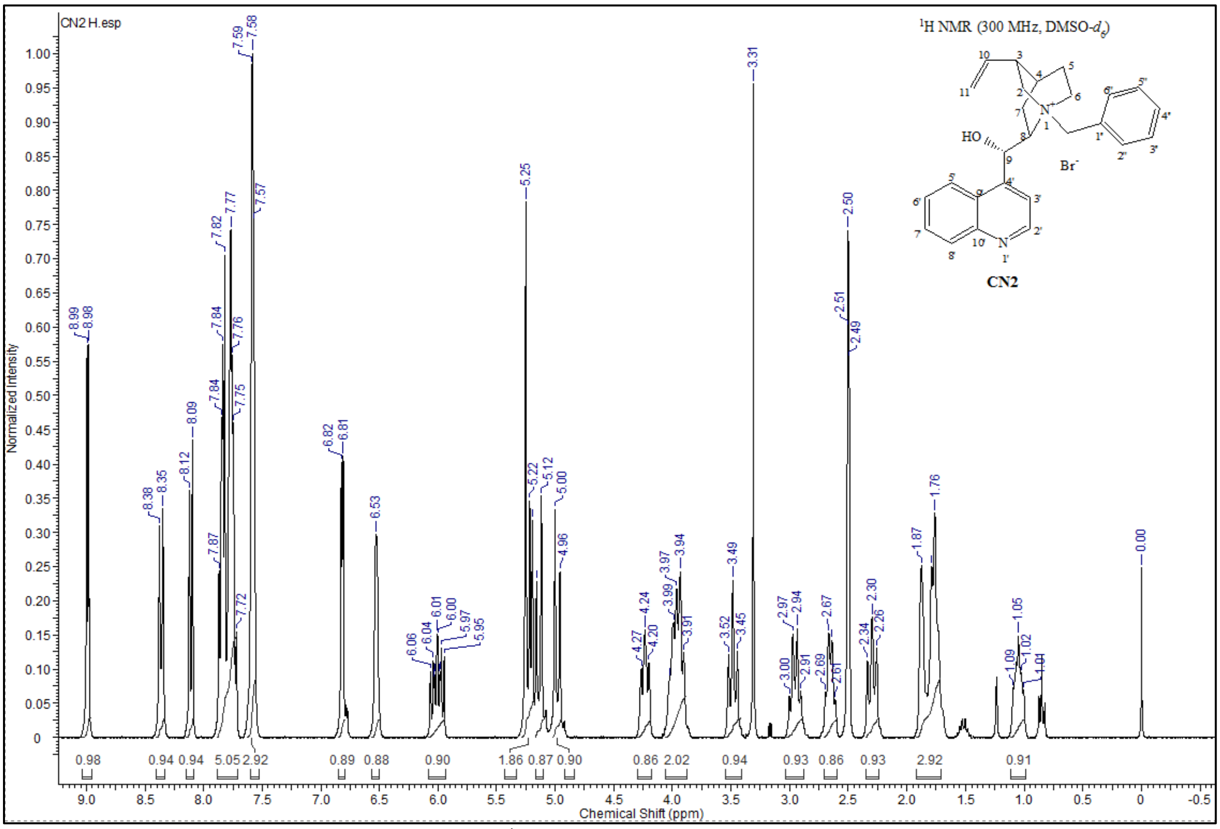

Figure 23. ¹H NMR spectra of **CN-Bzl**

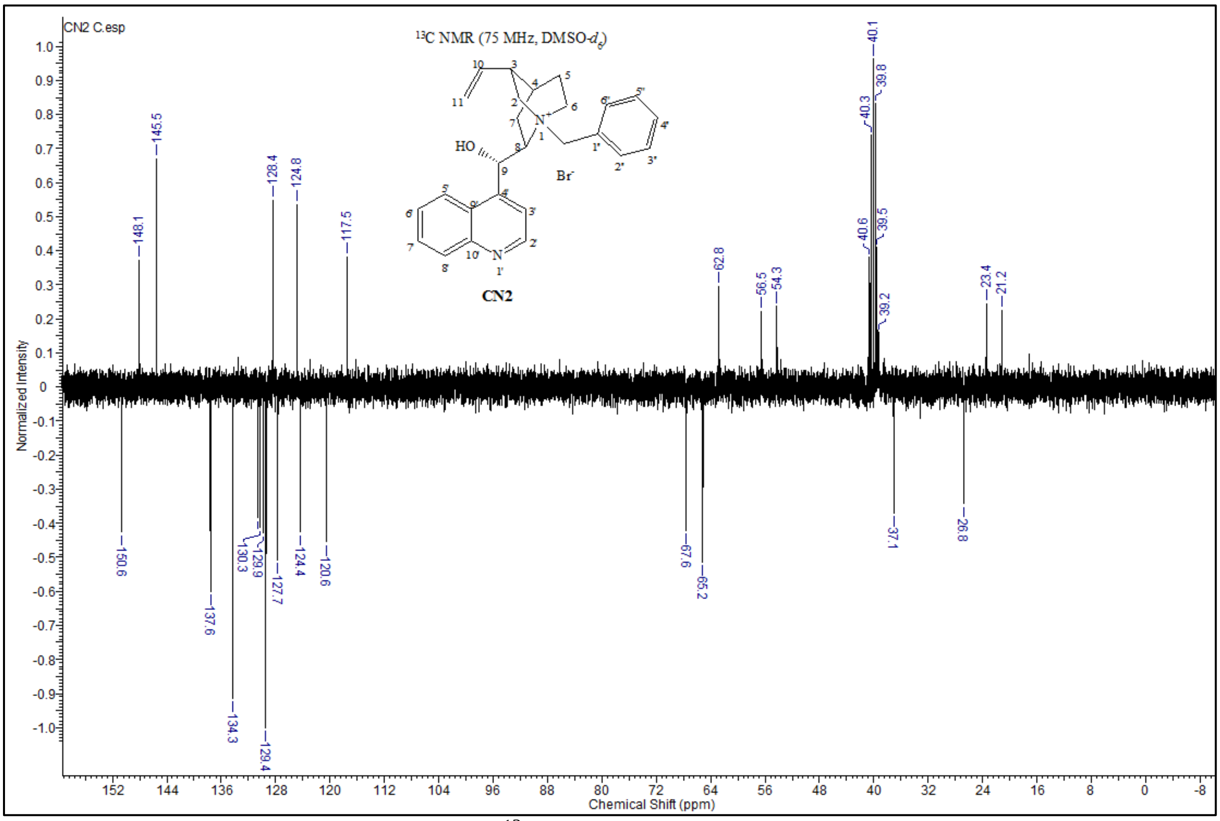

Figure 24. ¹³C NMR spectra of **CN-Bzl**

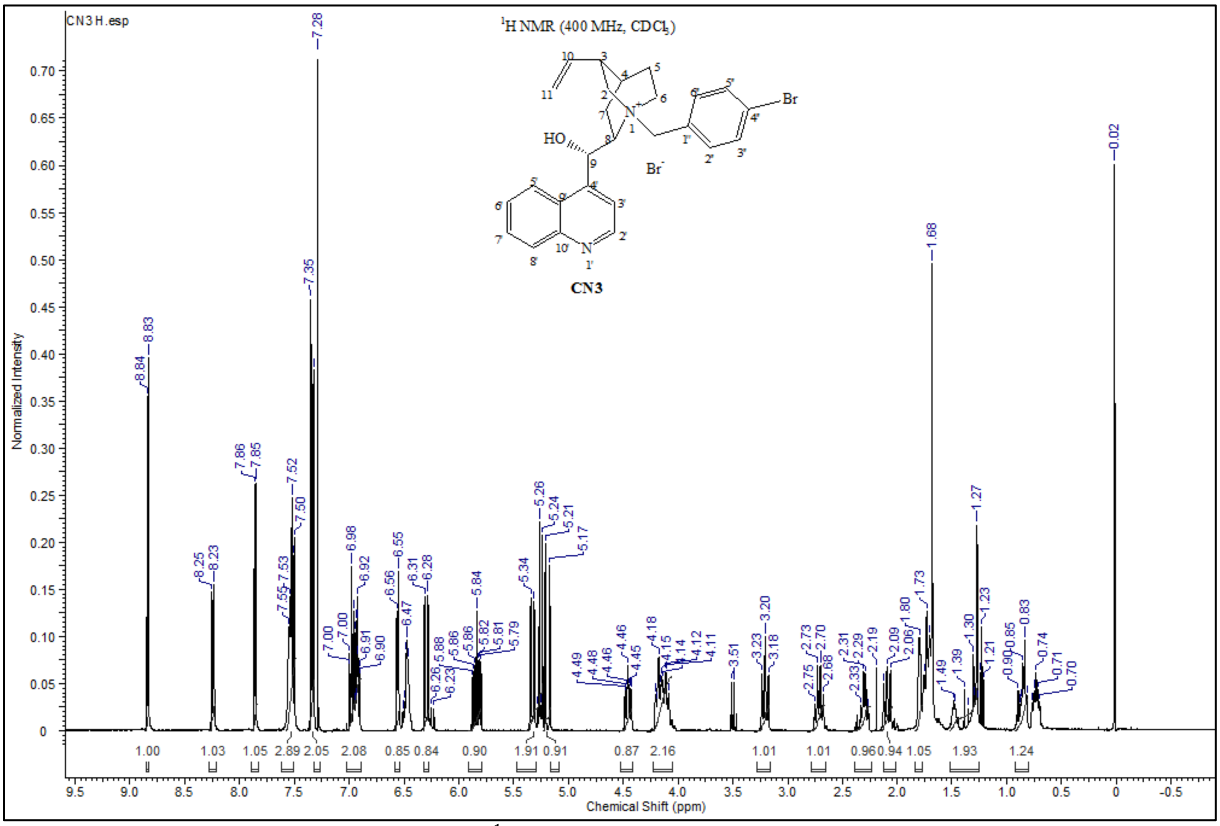

Figure 25. ^{1}H NMR spectra of **CN-(pBr)**

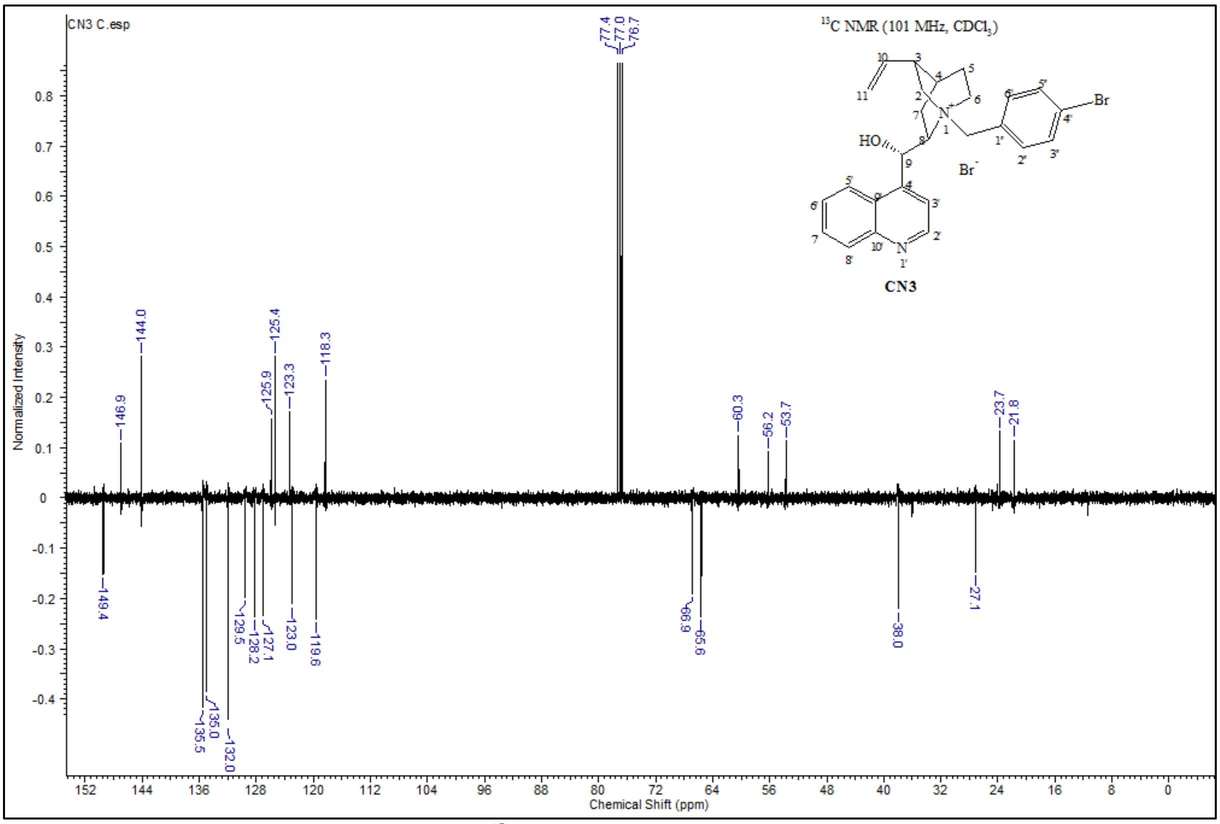

Figure 26. ^{13}C NMR spectra of **CN-(pBr)**

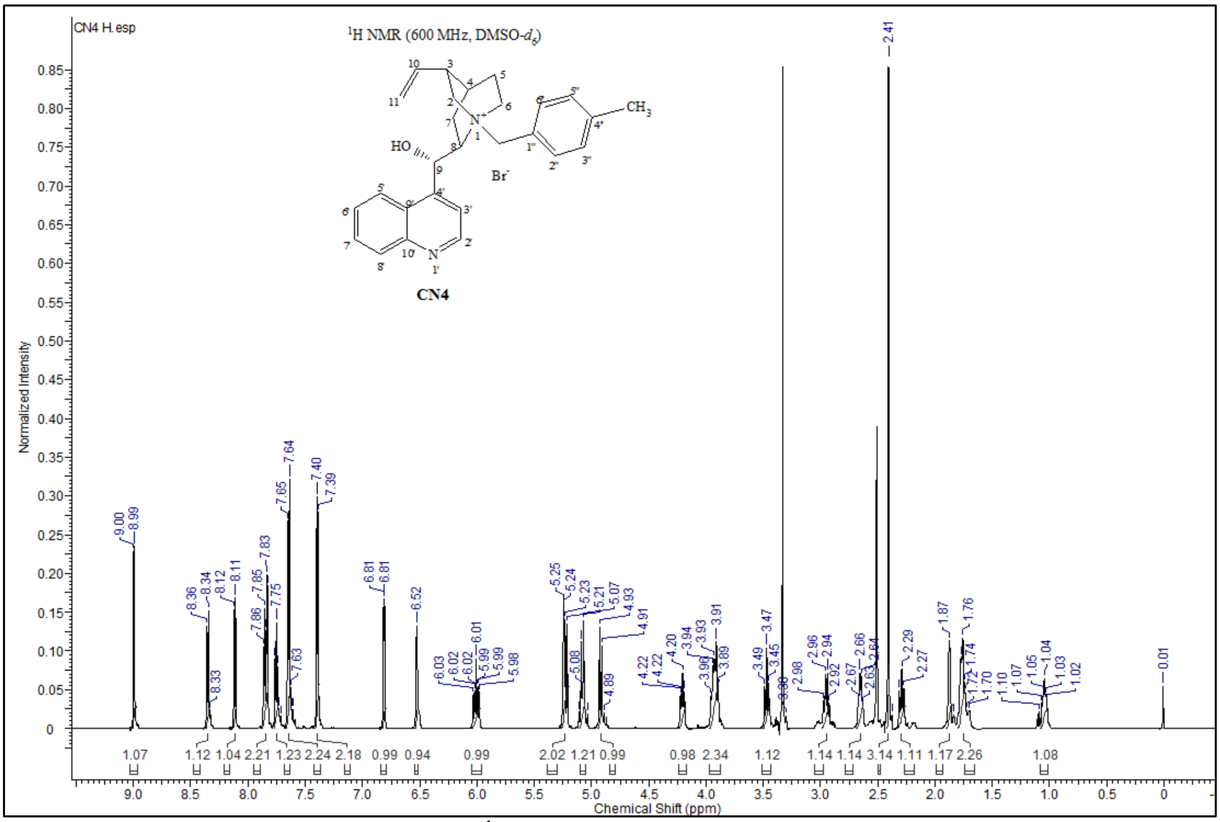

Figure 27. ^{1}H NMR spectra of **CN-(pMet)**

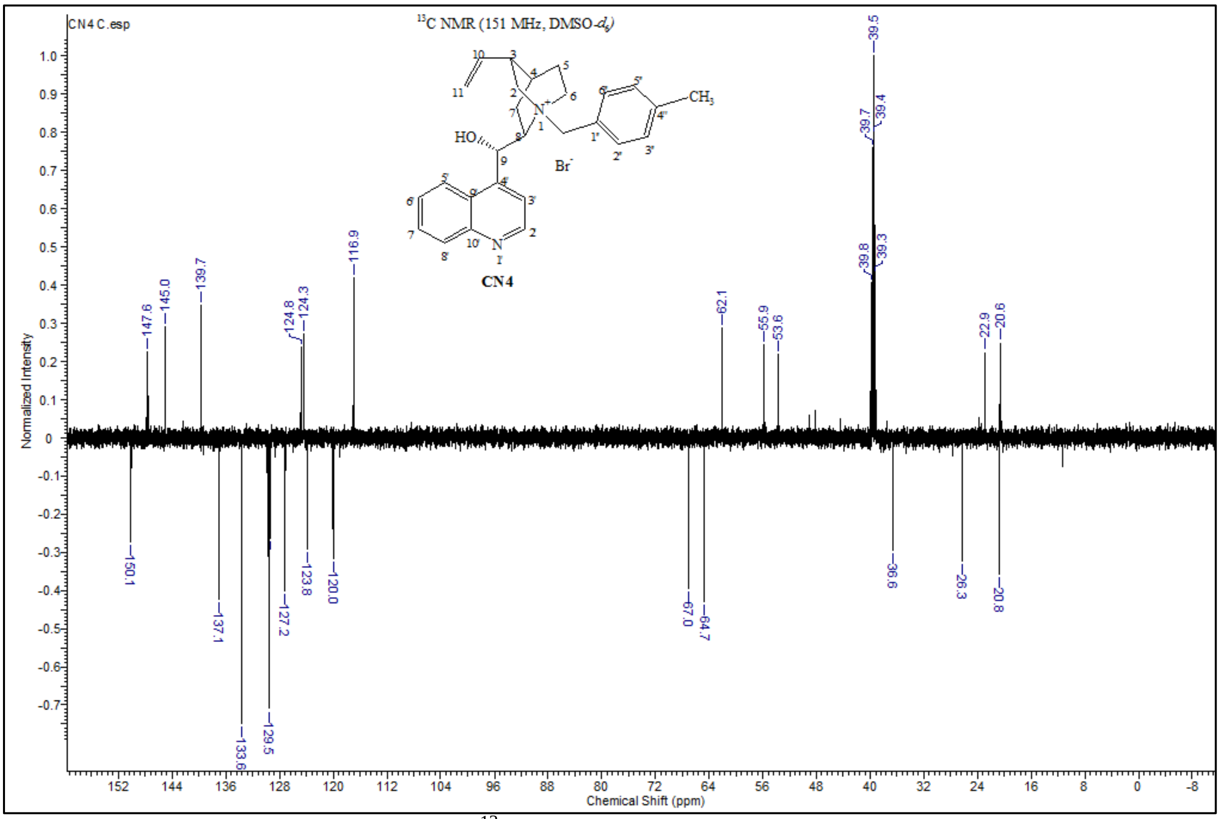

Figure 28. ^{13}C NMR spectra of **CN-(pMet)**

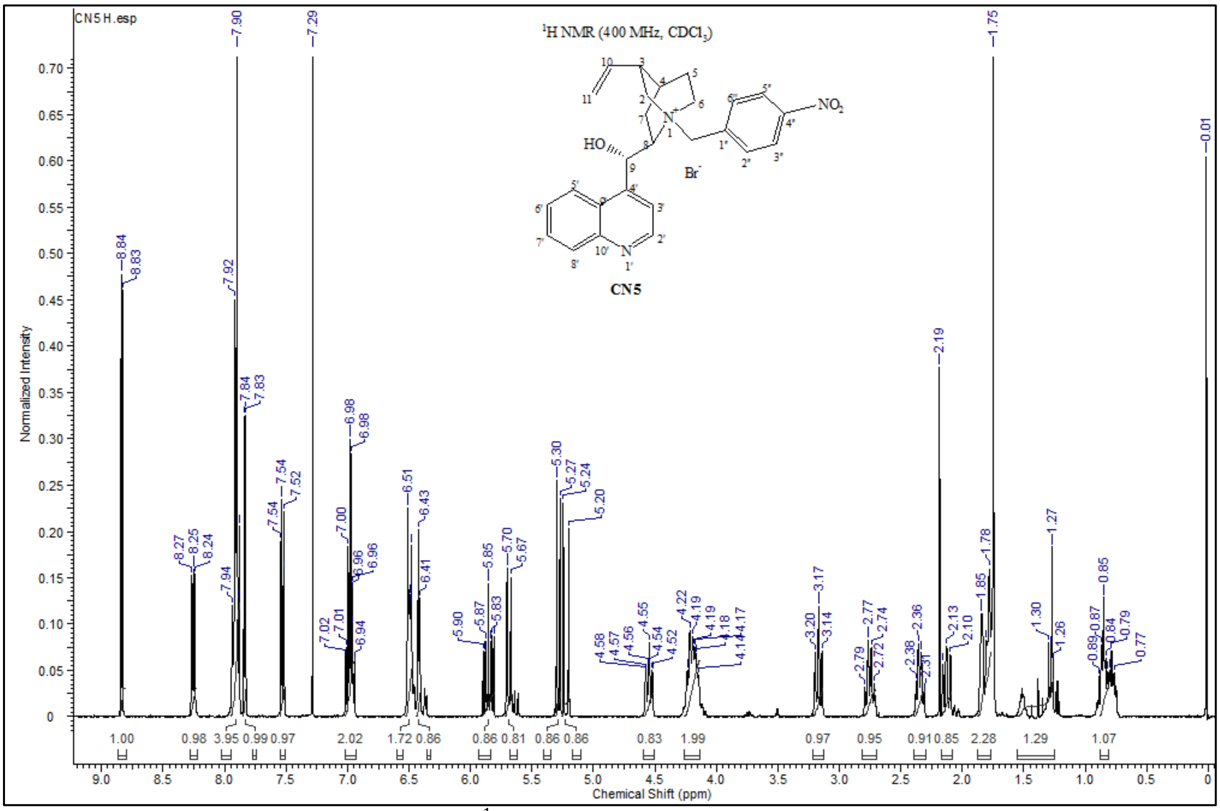

Figure 29. ^{1}H NMR spectra of **CN-(pNO$_2$)**

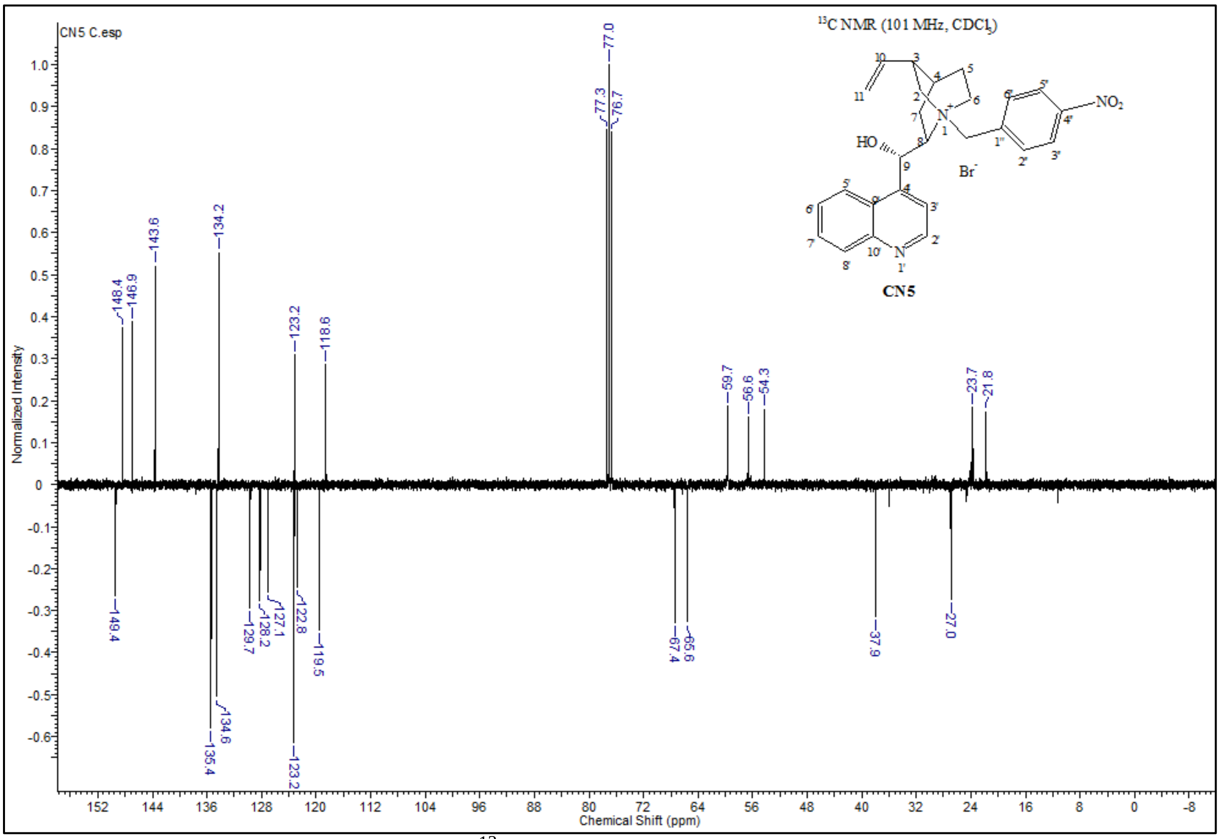

Figure 30. ^{13}C NMR spectra of **CN-(pNO$_2$)**

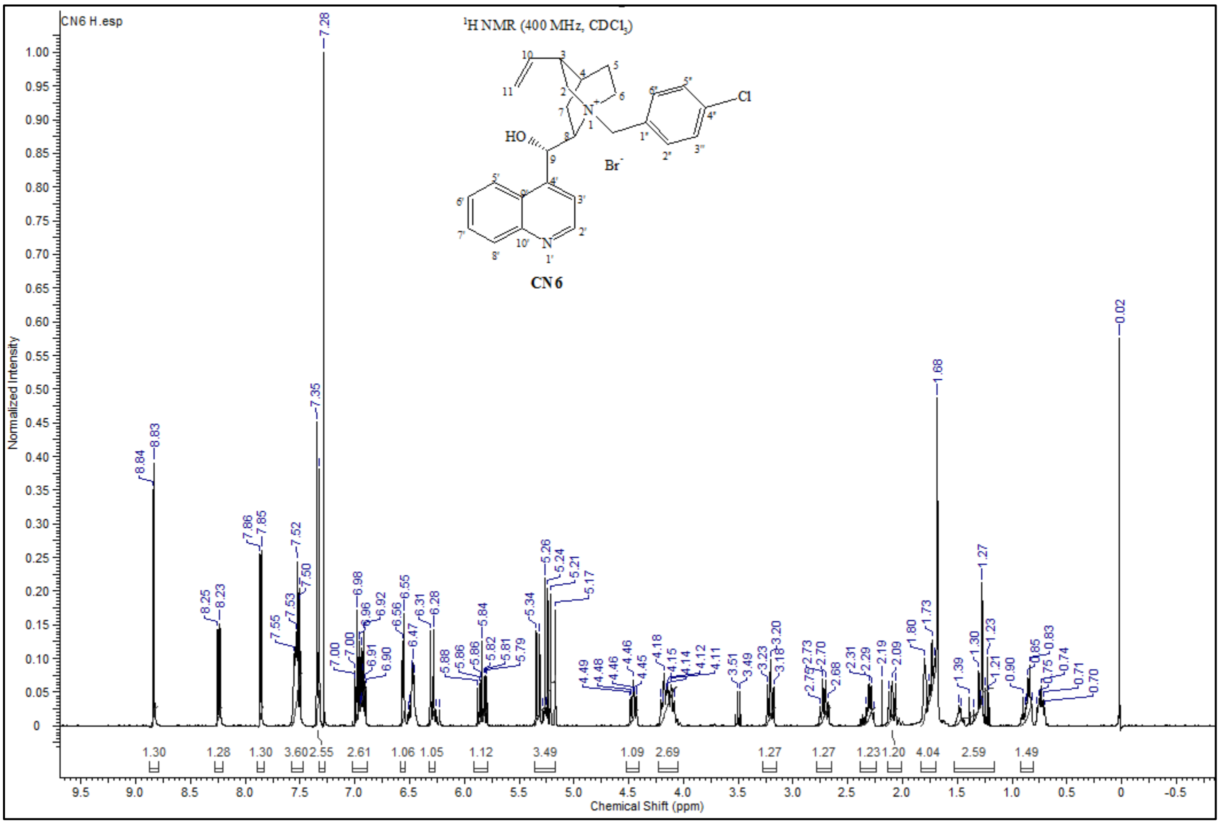

Figure 31. ^{1}H NMR spectra of **CN-(pCl)**

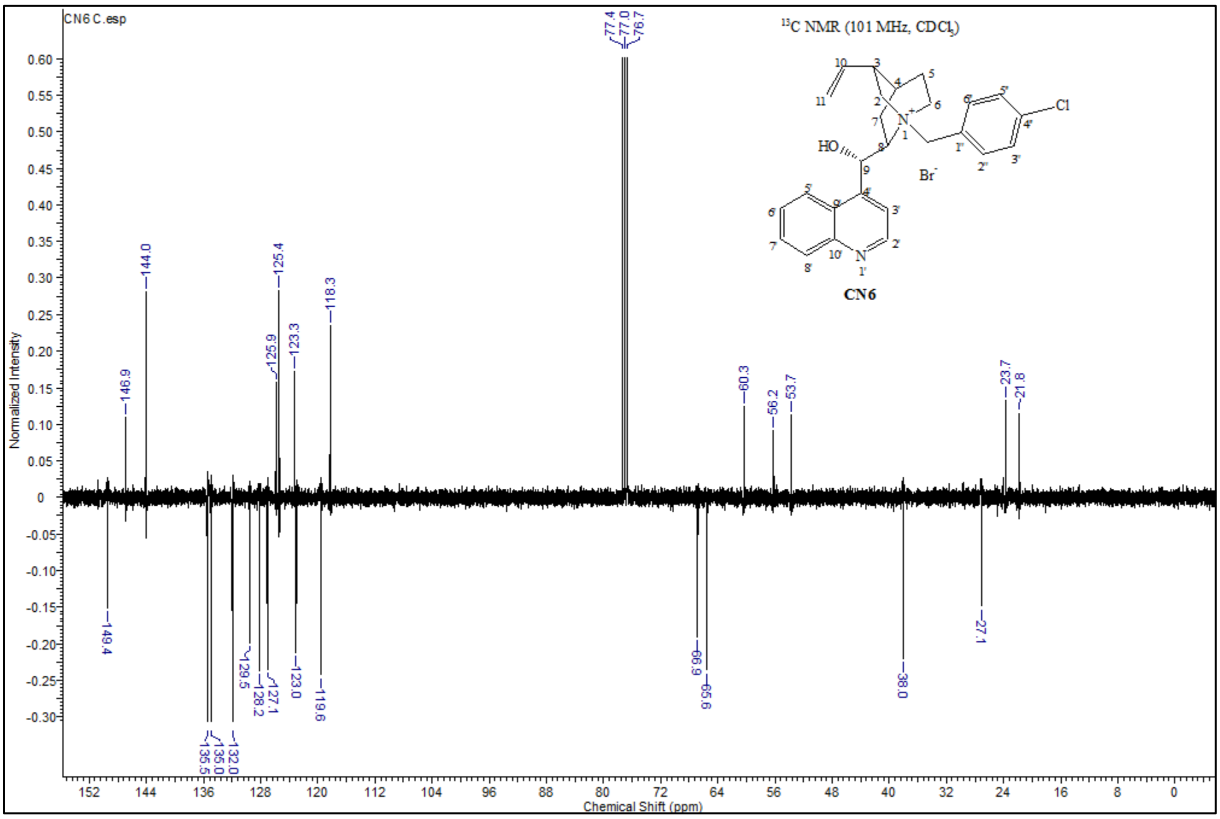

Figure 32. ^{13}C NMR spectra of **CN-(pCl)**

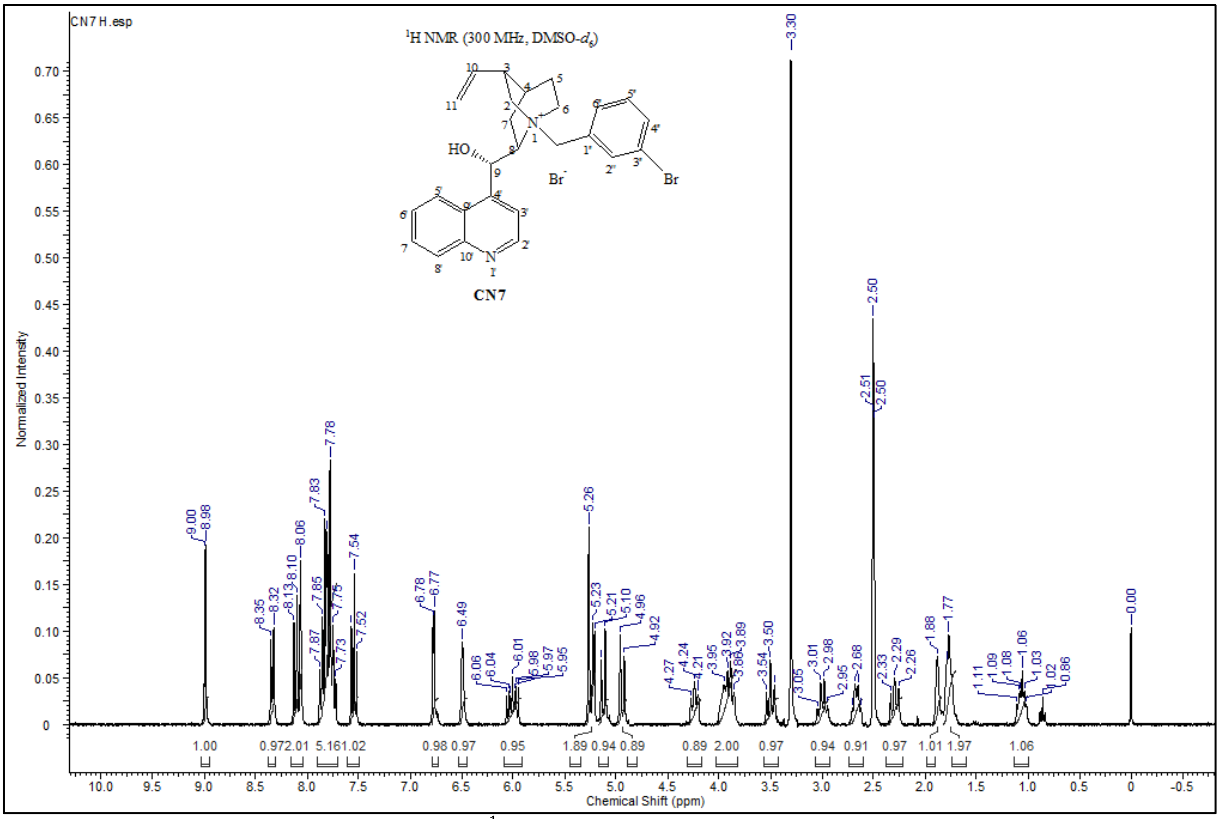

Figure 33. ^{1}H NMR spectra of **CN-(mBr)**

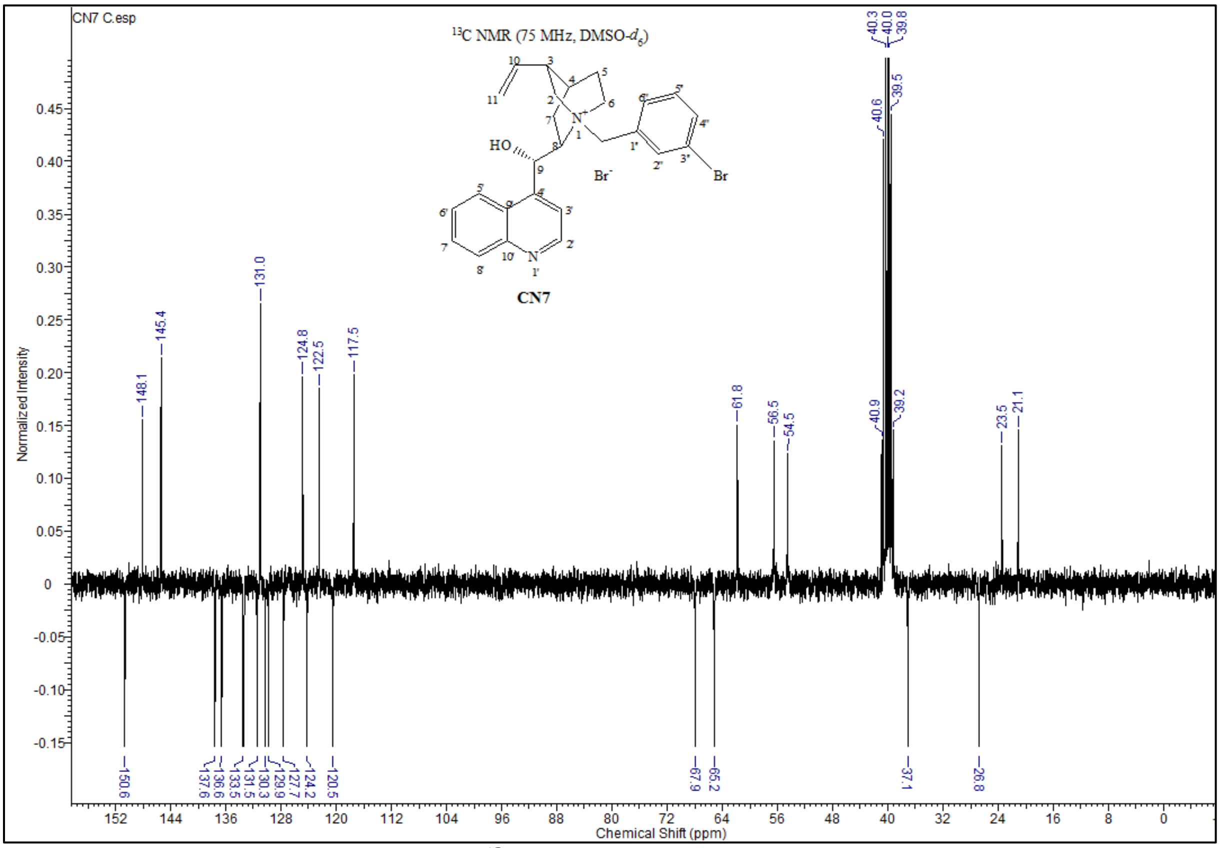

Figure 34. ^{13}C NMR spectra of **CN-(mBr)**

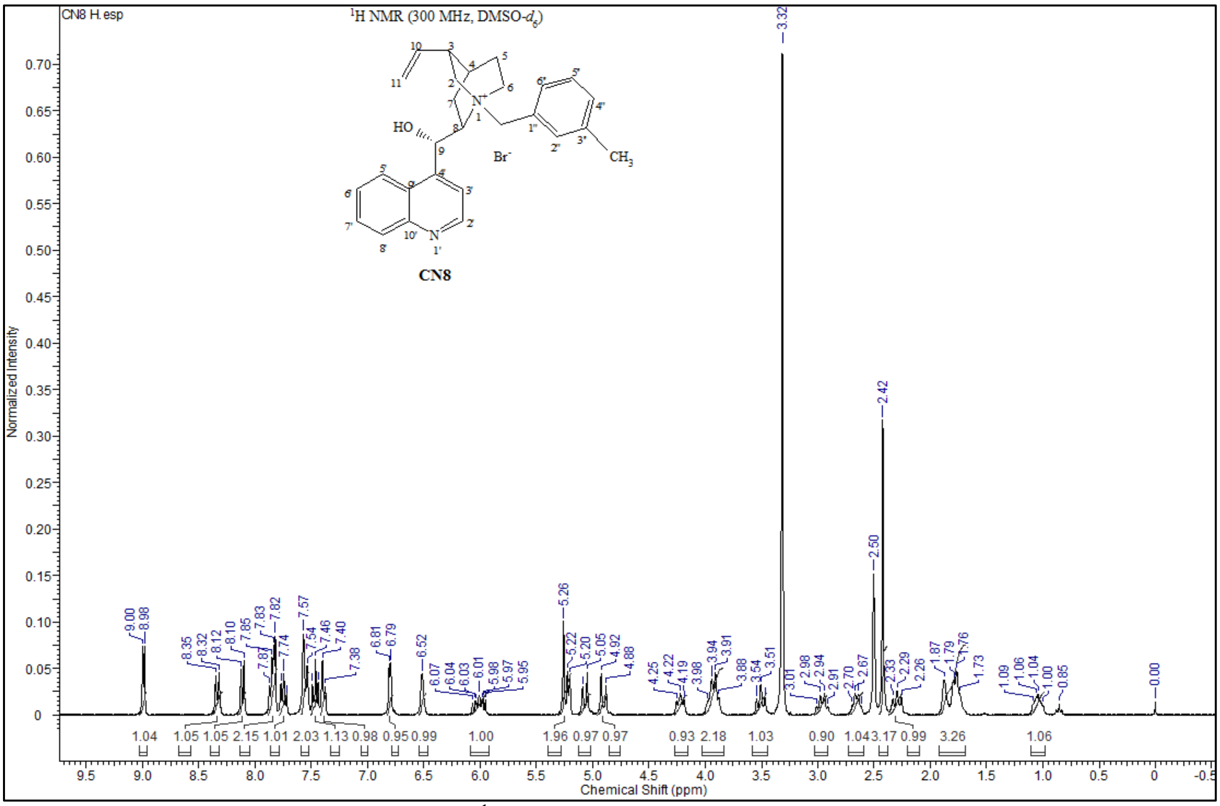

Figure 35. ^{1}H NMR spectra of **CN-(mMet)**

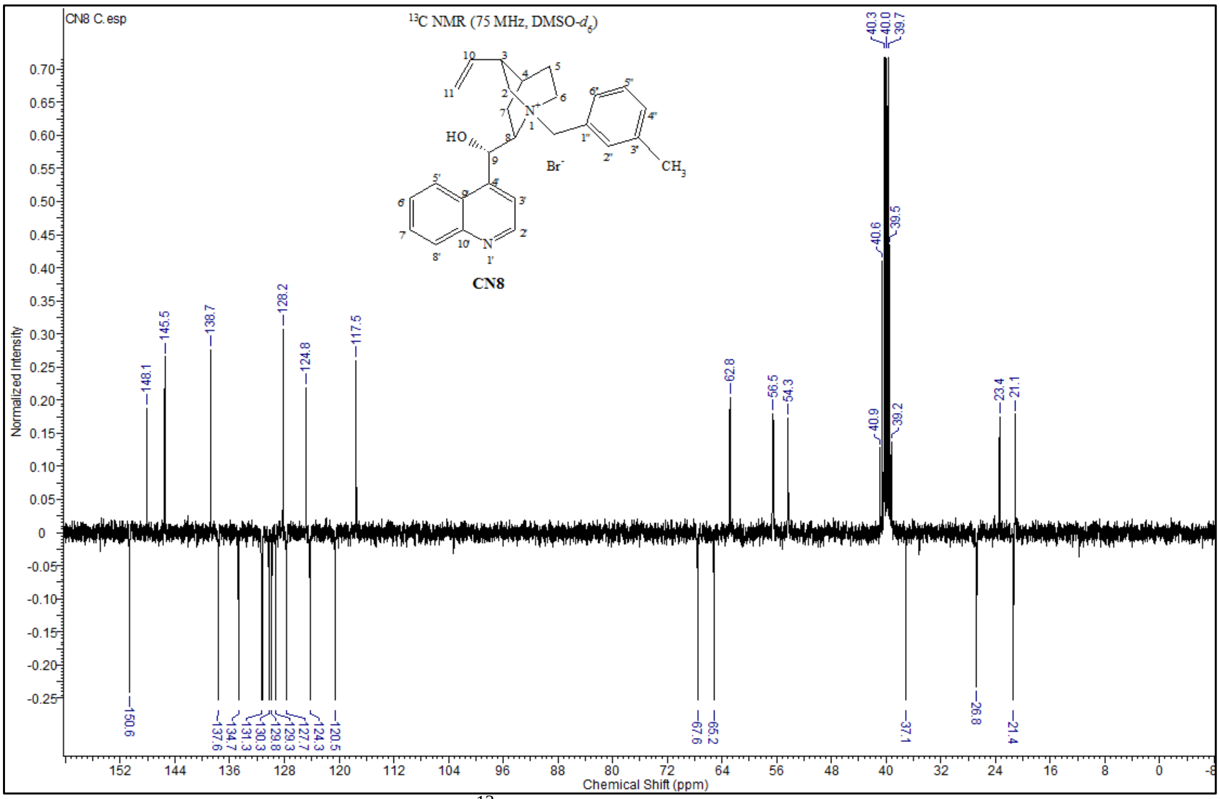

Figure 36. ^{13}C NMR spectra of **CN-(mMet)**

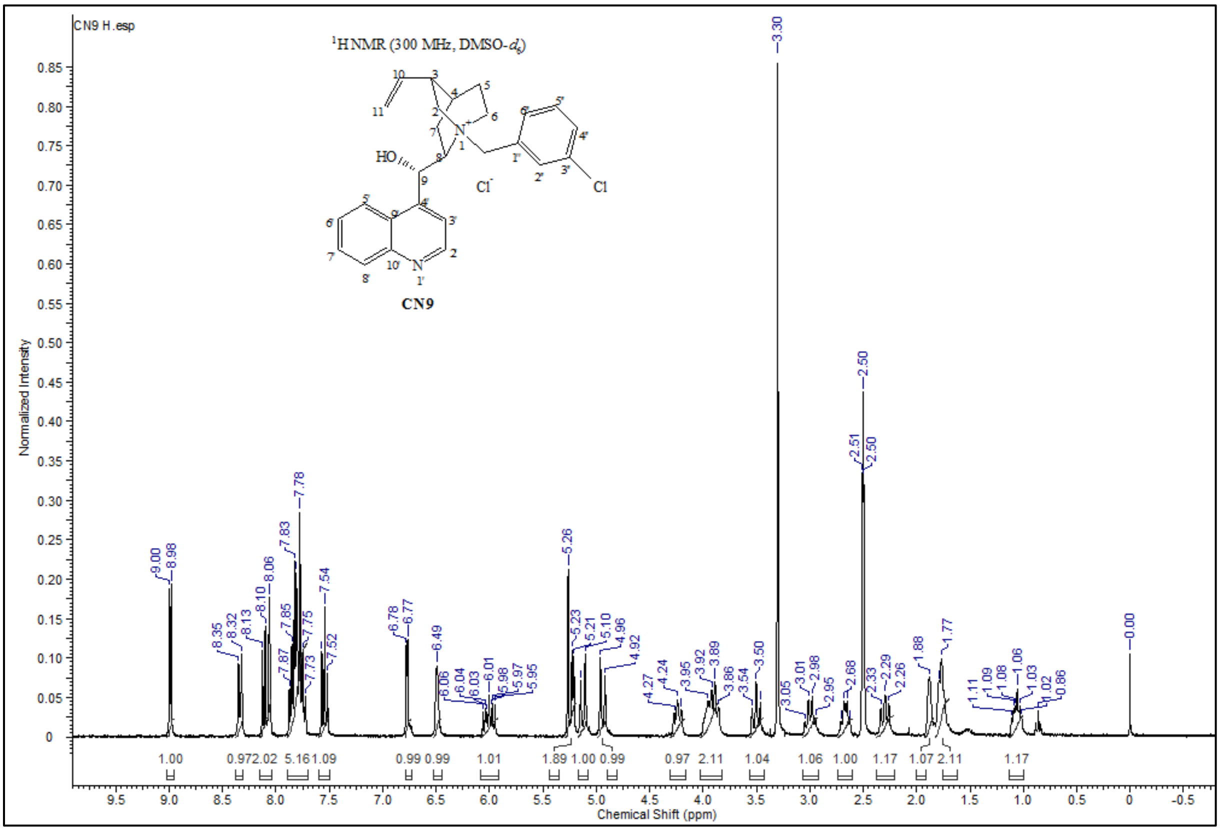

Figure 37. ^{1}H NMR spectra of **CN-(mCl)**

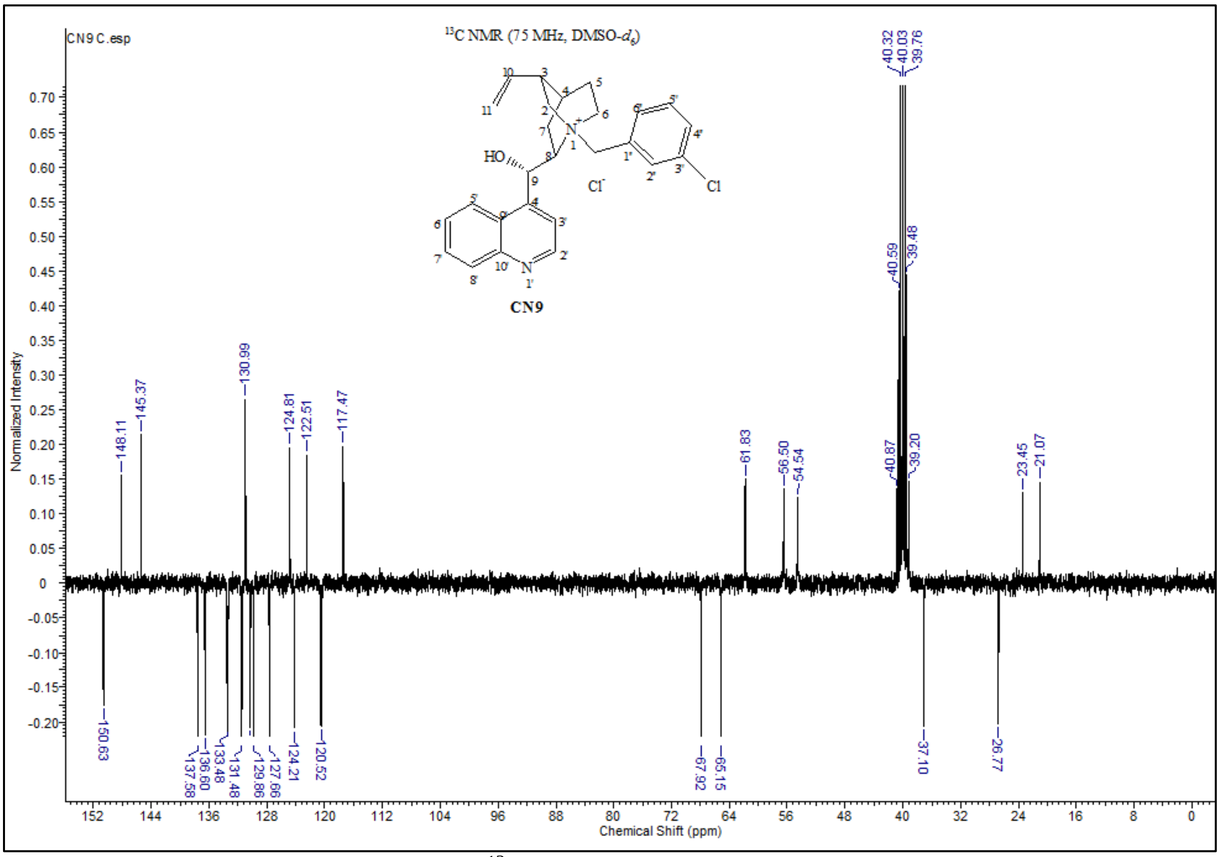

Figure 38. ^{13}C NMR spectra of **CN-(mCl)**

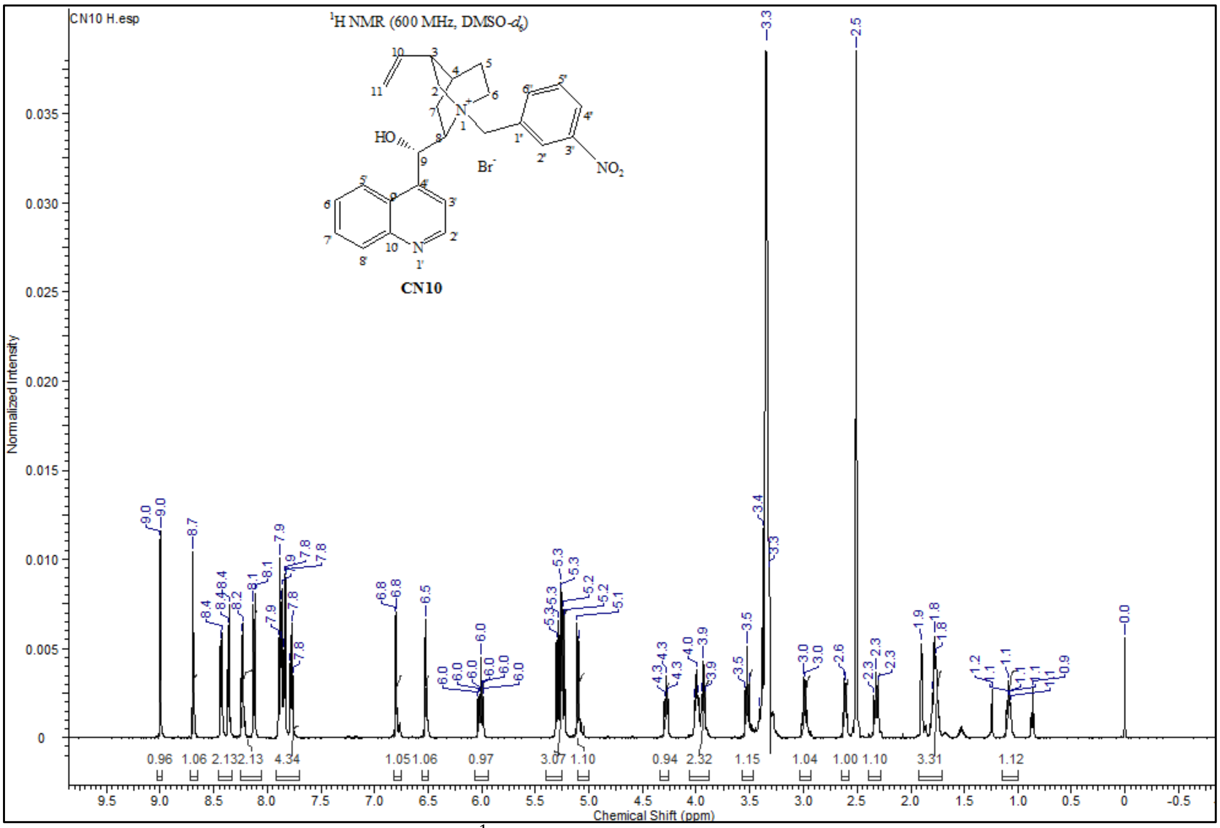

Figure 39. ^{1}H NMR spectra of **CN-(mNO$_2$)**

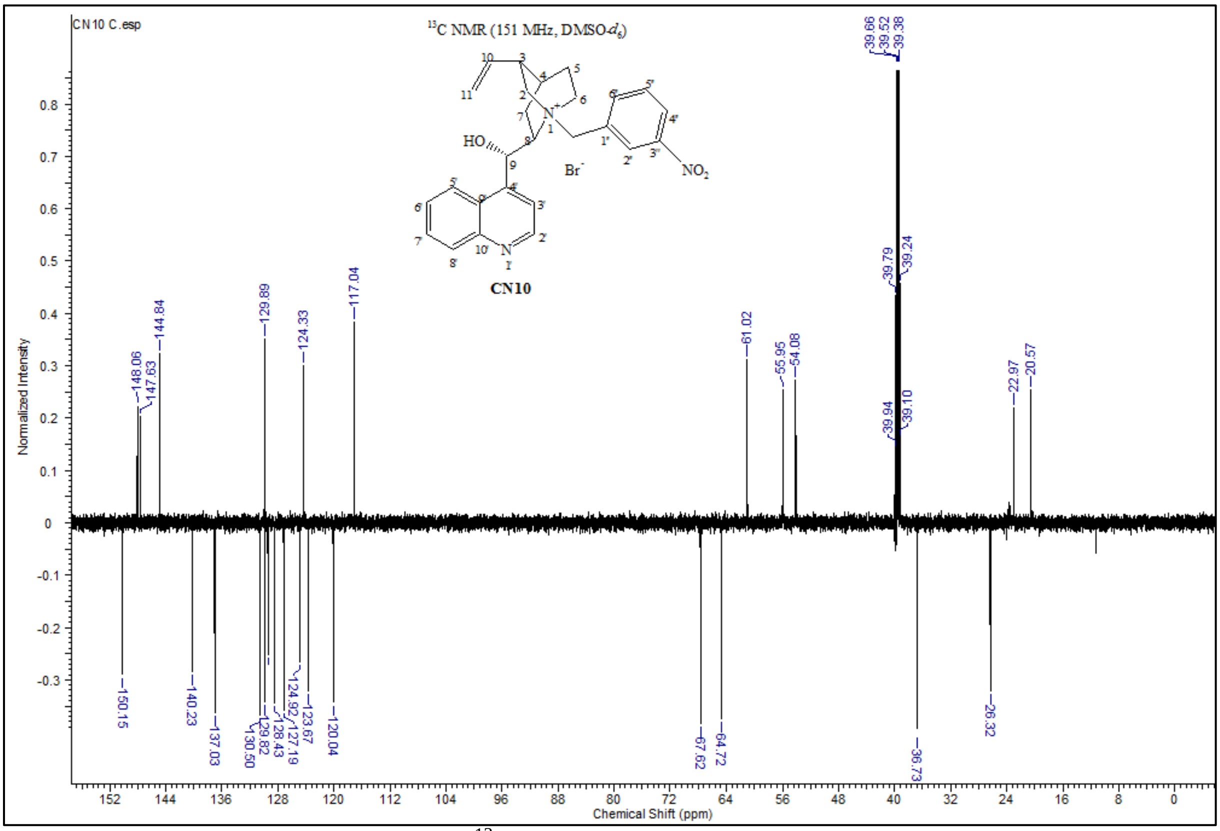

Figure 40. ^{13}C NMR spectra of **CN-(mNO$_2$)**
